# Cross-Cancer Pleiotropic Analysis Reveals Novel Susceptibility Loci for Lung Cancer

**DOI:** 10.3389/fonc.2019.01492

**Published:** 2020-01-15

**Authors:** Lijuan Wang, Meng Zhu, Yuzhuo Wang, Jingyi Fan, Qi Sun, Mengmeng Ji, Xikang Fan, Junxing Xie, Juncheng Dai, Guangfu Jin, Zhibin Hu, Hongxia Ma, Hongbing Shen

**Affiliations:** ^1^Department of Epidemiology and Biostatistics, Center for Global Health, School of Public Health, Nanjing Medical University, Nanjing, China; ^2^Jiangsu Key Lab of Cancer Biomarkers, Prevention and Treatment, Collaborative Innovation Center for Cancer Medicine, Nanjing Medical University, Nanjing, China

**Keywords:** lung cancer, genome-wide association studies, pleiotropy, susceptibility loci, gene expression

## Abstract

Genome-wide association studies (GWASs) have identified hundreds of single nucleotide polymorphisms (SNPs) associated with cancer risk, several of which have shown pleiotropic effects across cancers. Therefore, we performed a systematic cross-cancer pleiotropic analysis to detect the effects of GWAS-identified variants from non-lung cancers on lung cancer risk in 12,843 cases and 12,639 controls from four lung cancer GWASs. The overall association between variants in each cancer and risk of lung cancer was explored using sequential kernel association test (SKAT) analysis. For single variant analysis, we combined the result of specific study using fixed-effect meta-analysis. We performed functional exploration of significant associations based on features from public databases. To further detect the biological mechanism underlying identified observations, pathway enrichment analysis were conducted with R package “clusterProfiler.” SNP-set analysis revealed the overall associations between variants of 8 cancer types and lung cancer risk. Single variant analysis identified 6 novel SNPs related to lung cancer risk after multiple correction (*P*_fdr_ < 0.10), including rs1707302 (1p34.1, OR = 0.93, 95% CI: 0.90–0.97, *P* = 7.60 × 10^−4^), rs2516448 (6p21.33, OR = 1.07, 95% CI: 1.03–1.11, *P* = 1.00 × 10^−3^), rs3869062 (6p22.1, OR = 0.91, 95% CI: 0.86–0.96, *P* = 7.10 × 10^−4^), rs174549 (11q12.2, OR = 0.90, 95% CI: 0.87–0.94, *P* = 1.00 × 10^−7^), rs7193541 (16q23.1, OR = 0.93, 95% CI: 0.90–0.96, *P* = 1.20 × 10^−4^), and rs8064454 (17q12, OR = 1.07, 95% CI: 1.03–1.11, *P* = 4.30 × 10^−4^). The eQTL analysis and functional annotation suggested that these variants might modify lung cancer susceptibility through regulating the expression of related genes. Pathway enrichment analysis showed that genes modulated by these variants play important roles in cancer carcinogenesis. Our findings demonstrate the pleiotropic associations between non-lung cancer susceptibility loci and lung cancer risk, providing important insights into the shared mechanisms of carcinogenesis across cancers.

## Introduction

With rapidly increasing incidence and mortality rates, lung cancer has become the most frequently diagnosed cancer and the leading cause of cancer-related death in recent years. Based on GLOBOCAN 2012, there were 1.82 million new lung cancer cases (12.9% of the total cancer cases) and 1.59 million deaths (19.4% of the total cancer deaths) around the world (1). Although tobacco smoking has been confirmed as the main cause of lung cancer, genetic factors also determine lung cancer susceptibility (2). To identify genetic variants that contribute to lung cancer development, several GWASs have been performed and dozens of SNPs were identified over the past few years (3). However, these identified loci could explain only a small fraction of susceptibility. Thus, the challenge remains to detect additional risk loci with small effects, which may partially account for the missing heritability (4, 5).

Recent studies have identified that some genetic loci represent pleiotropic associations with multiple cancers (6–8). For example, genetic variants in the TERT-CLPTM1L region at 5p15.33 are associated with risk of lung, bladder, prostate, and cervical cancers (8). The discovery of pleiotropic effects may allow for the identification of shared genes and pathways that influence carcinogenesis across different cancers (9). In 2014, Park et al. evaluated the effects of 165 genetic variants associated with non-lung cancers on lung cancer susceptibility, which demonstrated novel susceptibility loci for lung cancer and indicated the commonality between lung cancer and other cancer types (10). However, the number of SNPs in the study by Park et al. was limited and subsequent GWASs have identified more susceptibility loci for cancers in recent years.

Thus, in this study, we comprehensively collected 1,915 GWAS loci associated with non-lung cancers from 5,876 publications and GWAS catalog database, and performed a systematic evaluation of possible pleiotropic associations with lung cancer risk. Our study could provide important insights into pleiotropic associations across cancers and better clarify the mechanism involved in lung cancer susceptibility.

## Methods

### Study Participants

Data from four existing lung cancer GWASs including 12,843 lung cancer cases and 12,639 controls was used in this study: (i) Nanjing Medical University (**NJMU**) GWAS including 2,331 lung cancer cases and 3,077 controls (11), (ii) Female Lung Cancer Consortium in Asia (**FLCCA**) GWAS with 4,796 lung cancer cases and 3,741 controls (12), (iii) Environment and Genetics in Lung Cancer Etiology (**EAGLE**) GWAS consists of 1,937 lung cancer cases and 1,984 controls and (iv) Division of Cancer Epidemiology and Genetics (**DCEG**) Lung Cancer GWAS encompasses 3,779 cases and 3,837 controls (13). Briefly, two Asian GWASs and two European GWASs were included in our study to explore the overall genetic effects of variants. The basic demographic information of participants involved are shown in Table 1. Informed consent was obtained from each subject, and this study was approved by the institutional review boards of each participating institution.

**Table 1:** Basic characteristics and clinical features of participants in each dataset.

	**NJMU**		**FLCCA**		**EAGLE**		**DCEG**	
	**Cases**	**Controls**	**Cases**	**Controls**	**Cases**	**Controls**	**Cases**	**Controls**
**Sample size**	2,331	3,077	4,796	3,741	1,937	1,984	3,779	3,837
**Gender**								
Male	1,711	2,086	n/a	n/a	1,532	1,519	2,926	3,375
Female	620	991	4,796	3,741	405	465	853	462
**Smoking**								
Never	825	1,768	4,796	3,741	138	636	n/a	n/a
Former	254	226	n/a	n/a	821	855	n/a	n/a
Current	1,252	1,083	n/a	n/a	966	488	n/a	n/a
Missing information	n/a	n/a	n/a	n/a	12	5	n/a	n/a
**Age**								
<60	1,111	1,429	2,164	1,745	423	502	1,402	1,315
≥60	1,220	1,648	2,632	1,996	1,514	1,482	2,377	2,522
**Histology**								
Squamous cell carcinoma	822	n/a	660	n/a	492	n/a	n/a	n/a
Adenocarcinoma	1,304	n/a	3,469	n/a	795	n/a	n/a	n/a
Other^a^	205	n/a	667	n/a	616	n/a	n/a	n/a
Missing information	n/a	n/a	n/a	n/a	34	n/a	n/a	n/a

*n/a, not available or non-existent*.

a *Other histological types include small cell lung cancer, large cell lung cancer and mixed cell lung cancer*.

### Quality Control and Imputation of GWAS Data

The detail about quality control and imputation has been described in our previous study (11). Briefly, individuals with call rates <95%, familial relationships or extreme heterozygosity rates were excluded. We selected SNPs based on the following criteria: (i) call rates >95%, (ii) minor allele frequencies (MAFs) >0.05, (iii) *P* > 1 × 10^−6^ for Hardy-Weinberg equilibrium (HWE). We then phased the haplotypes with Shapeit (14) and performed imputations with IMPUTE2 (15) taken the 1,000 Genomes Project Phase III data as reference. We ruled out SNPs with imputation quality score (INFO) <0.4, MAF <0.01, and HWE *P* < 1 × 10^−6^. Quality control procedure was performed using PLINK1.9 software.

### SNP Selection

**Figure 1:**
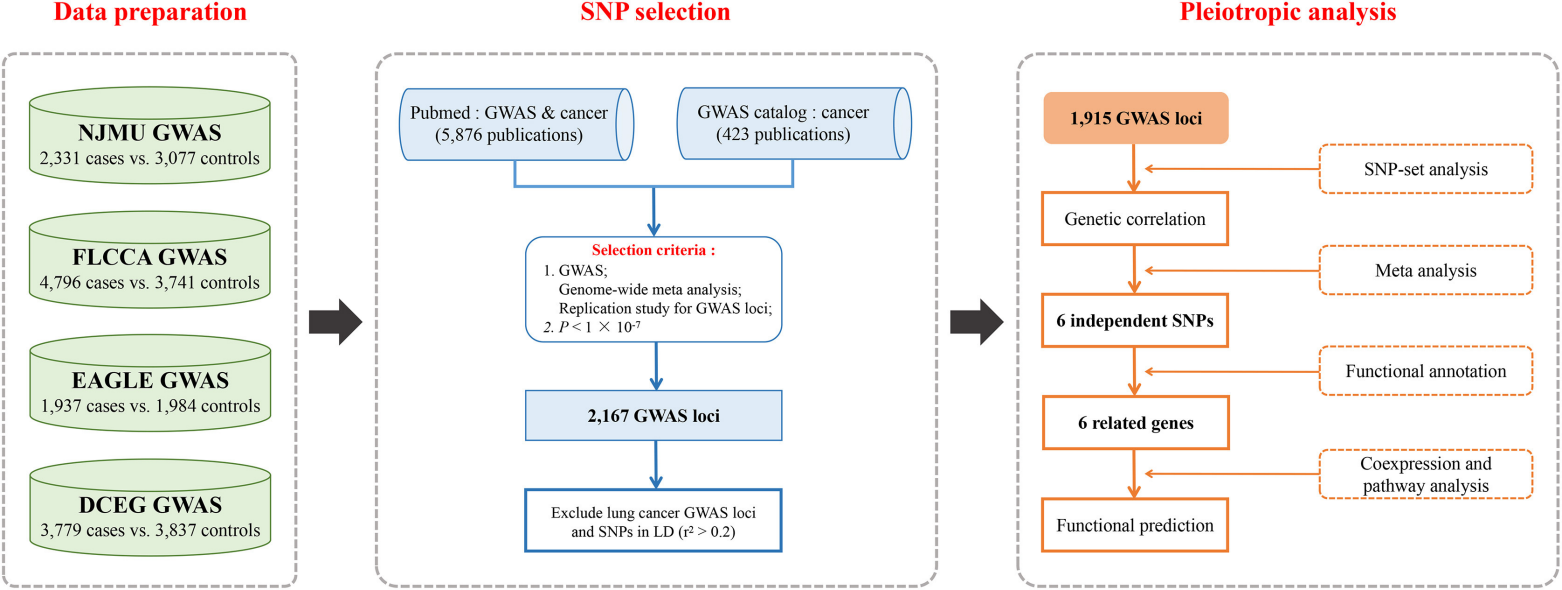
Flowchart for (1) Four existing lung cancer GWASs were included in the study, and standard quality control and imputation were performed for eligibility; (2) SNP selection strategy based on publications as of July 2018 on GWASs and cancers in PubMed and GWAS Catalog was used as a supplement; (3) Pleiotropic analysis of the effects of GWAS-identified risk variants from non-lung cancers on lung cancer risk both in general and in single variant: correlations between non-lung cancers and lung cancer by SNP-set analysis, and associations of non-lung cancer susceptibility loci with lung cancer risk, and functional exploration for these variants and related genes.

We undertook a comprehensive systematic review of publications on GWASs and cancers in PubMed using the Mesh Term “Genome-wide association study” or “GWAS” and “cancer.” A total of 5,876 abstracts and if necessary the full texts were screened for eligibility. Among them, GWASs, genome-wide meta analyses and replication studies for GWAS loci were evaluated. Additionally, SNPs associated with cancers as of July 2018 from the NHGRI GWAS catalog were also included. Finally, a total of 2,167 SNPs beyond the threshold of significance (*P* < 1 × 10^−7^) remained. After that, we excluded lung cancer GWAS loci as well as those in the same linkage disequilibrium (LD) blocks (*r*^2^ > 0.2), and 1,915 SNPs within 15 cancer types were remained. The search strategy is shown in Figure 1.

### SNP-Set Analysis by Cancer Types

The aim of SNP-set analysis was to explore the overall associations of variants identified from other cancer types with the risk of lung cancer. SNP-set analysis was conducted using SKAT-C package, which tests for association between groups of SNPs and a phenotype by aggregating the weighted variance-component score statistics for each SNP within a group using kernel function (16). In this case, SNPs in LD (*r*^2^ > 0.2) with lung cancer GWAS loci were excluded. We divided the remained variants into different groups by cancer types and tested the correlations with lung cancer. Age, gender, smoking status and principal components (PCA) were adjusted in the SNP-set analysis. We used Benjamini-Hochberg method for multiple correction and cancers with FDR less than 0.05 in the combined dataset were considered to be significantly correlated with lung cancer.

### Single Variant Analysis

We used an additive effect model to test the associations of variants with lung cancer in each dataset. Then, meta-analysis was performed using a fixed-effect model. We calculated the index of heterogeneity (*I*^2^) and SNPs with high heterogeneity (*I*^2^ > 75%) were excluded. Then, Benjamini-Hochberg method was used to correct multiple testing and FDR less than 0.10 in the combined dataset was considered as a cutoff threshold. In addition, subgroup analyses by age (≤ 60 and >60), gender (male and female), smoking status (never and ever), and tumor histology (adenocarcinoma, squamous cell carcinoma and other types of lung cancer) and following gene-environment interaction analysis were performed to further explore the interaction between variants and smoking. Association analyses were conducted with PLINK1.9 while general statistical analyses were carried out using R (R 3.5.0).

### Functional Explorations of Significant Associations

We performed annotations for variants within promising genes using ANNOVAR software (17). We applied the SIFT (18) and PolyPhen databases (19) to predict the function of exonic variants. To investigate the potential function of association at non-coding regions, we utilized data from the Genotype-Tissue Expression (GTEx, version 7) to perform the expression quantitative trait loci (eQTL) analysis in 383 lung tissue samples. Then, we annotated SNPs to regulatory elements including the histone Chip-seq (H3K27AC, H3K4ME1, H3K4ME3) peaks, DNase I hypersensitivity sites (DHS) and transcription factor binding sites (TFBS) from ENCODE Project Consortium. All these features estimated in A549 cell lines were downloaded from the UCSC website. In addition, we used RegulomeDB database to further evaluate regulatory potential for identified variants.

### Co-expression and Pathway Enrichment Analysis

Based on results from functional annotation, we defined SNP-related genes with the following criteria: (i) locate within LD blocks where the identified variants as well as their related SNPs (*r*^2^ > 0.6) reside in, (ii) show most significant cis-eQTL associations with identified variants or bear exonic mutations that affect the function of proteins. In order to test whether these identified related genes were associated with lung cancer susceptibility, we performed gene-based analysis with MAGMA software, which is a powerful tool using multiple regression approach to detect multi-marker effects for a genome-wide gene association analysis (20). To explore biological function and alternative pathways of these related genes, which help to explain their pathogenic mechanism involved in the development of lung cancer, we conducted co-expression and pathway enrichment analysis based on GTEx V7 database and Kyoto Encyclopedia of Genes and Genomes (KEGG, 186 pathways) were used as reference. We used linear regression model to detect co-expressed genes and Bonferroni method was utilized for multiple correction. Significant co-expressed genes with adjusted *P*-value < 0.05 were included in pathway analysis performed by “clusterProfiler” package (21).

## Results

### Correlations Between Non-lung Cancers and Lung Cancer

**Figure 2:**
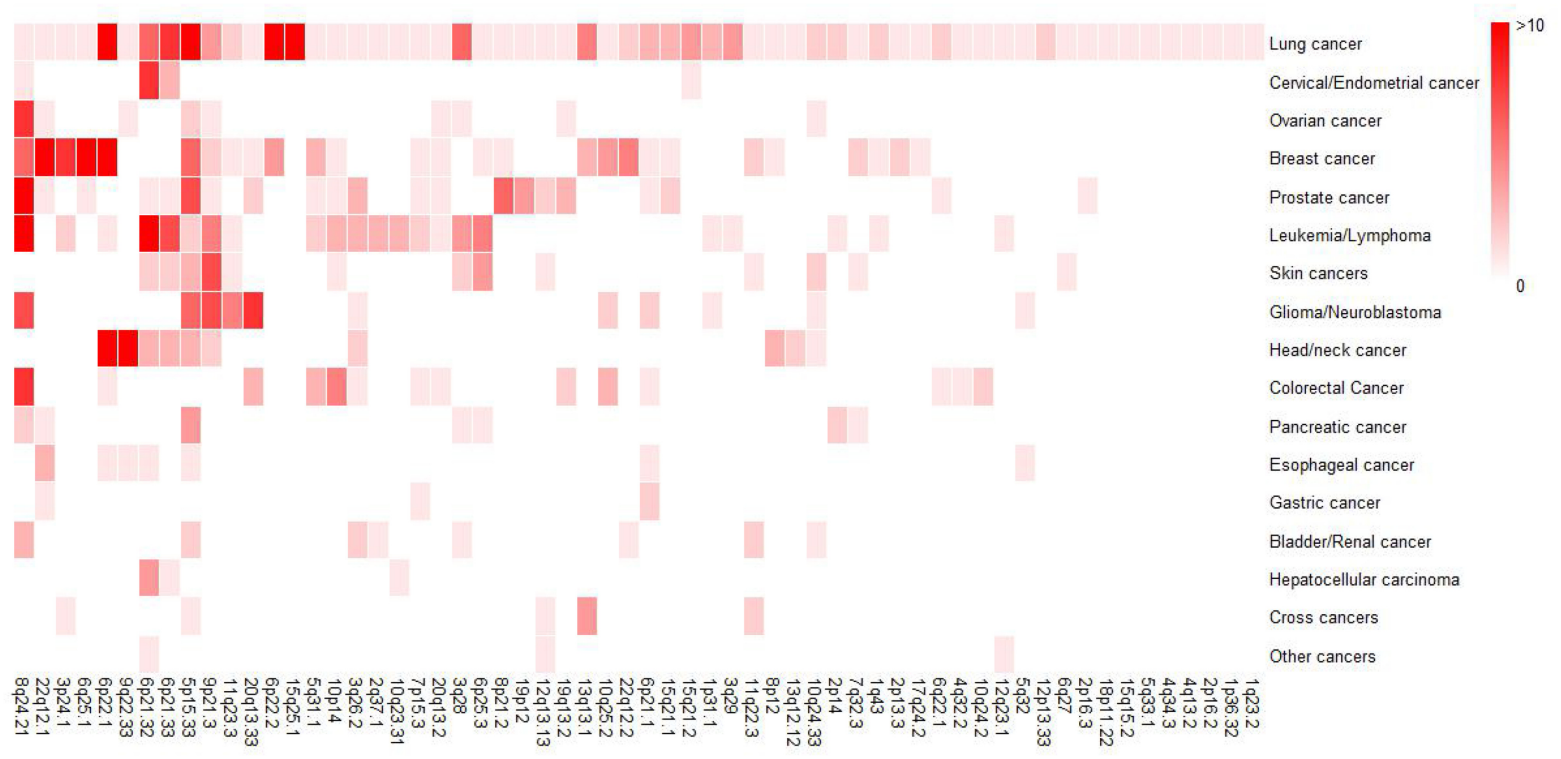
Heatmap for a general overview of susceptibility bands for each cancer type. For non-lung cancers, we included susceptibility bands overlapped with that of lung cancer. The intensity of color represented the number of GWAS susceptibility loci in the band. Therefore, darker colors indicates more susceptibility loci.

In order to obtain a general overview of susceptibility regions for each cancer, we collected the reported GWAS loci and mapped them to particular band of chromosome according to hg19, which was the susceptibility band we mentioned below. To date, 60 susceptibility bands have been identified for lung cancer and 50 of them were overlapped with the bands of non-lung cancer (Figure 2), further suggesting that some genomic bands are associated with multiple cancers. Here, we defined susceptibility bands shared by more than half of cancers (i.e., shared by at least 8 cancer types) as cancer enriched bands, and 4 (5p15.33, 6p21.32, 8q24.21, 9p21.3) met the criterion and showed pleiotropic associations with multiple cancers. Supplementary Table 1 displayed an overview of GWAS loci located in cancer enriched bands identified in different cancers, some of which showed significant associations with lung cancer risk, indicating the correlations between non-lung cancers and lung cancer.

In the SNP-set analysis, we found that the GWAS SNPs of 8 cancer types, including, cervical/endometrial, bladder/renal, prostate, pancreatic, ovarian, leukemia/lymphoma, esophageal, and colorectal cancer, were significantly associated with lung cancer risk in the combined dataset (FDR <0.05) (Supplementary Table 2). To rule out the impact of shared bands among different cancers (i.e., MHC), we excluded variants within MHC as well as the 4 cancer enriched bands mentioned above and re-conducted a SNP-set analysis. Similar associations were observed in cervical/endometrial, bladder/renal, prostate, ovarian, leukemia/lymphoma, esophageal, and colorectal cancer, which were still correlated with lung cancer (Supplementary Table 3).

### Single Variant Analysis and Subgroup Analysis

After combining results from four GWAS datasets of lung cancer, we identified 17 SNPs that were significantly related to lung cancer susceptibility (*P*_fdr_ < 0.10) (Supplementary Table 4). For variants in the same LD block (*r*^2^ > 0.2), the most significant one remained and 6 independent SNPs were showed in Table 2. Supplementary Table 5 displays the association results of these 6 variants in each separate cohort. The regional plots of these 6 variants were presented in Supplementary Figure 1. Among these 6 identified novel susceptibility loci, rs2516448 (6p21.33, OR = 1.07, 95% CI: 1.03–1.11, *P* = 1.00 × 10^−3^) and rs3869062 (6p22.1, OR = 0.91, 95% CI: 0.86–0.96, *P* = 7.10 × 10^−4^) reside in known lung cancer susceptibility regions, but were independent from previously reported SNPs of lung cancer (Supplementary Table 6); while rs1707302 (1p34.1, OR = 0.93, 95% CI: 0.90–0.97, *P* = 7.60 × 10^−4^), rs174549 (11q12.2, OR = 0.90, 95% CI: 0.87–0.94, *P* = 1.00 × 10^−7^), rs7193541 (16q23.1, OR = 0.93, 95% CI: 0.90–0.96, *P* = 1.20 × 10^−4^) and rs8064454 (17q12, OR = 1.07, 95% CI: 1.03–1.11, *P* = 4.30 × 10^−4^) were located in novel susceptibility bands for lung cancer and were firstly identified to be correlated with lung cancer risk in this study.

**Table 2:** Independent associations of significant locus with lung cancer risk.

**Band**	**Related gene**	**SNP**	**EA/NEA**	**OR (95%CI)^**a**^**	***P*^**a**^**	**FDR^**a**^**	**Source of Cancer**
1p34.1	MAST2	rs1707302	A/G	0.93(0.90-0.97)	7.60E-04	6.60E-02	Breast cancer
6p21.33	MICA	rs2516448	T/C	1.07(1.03-1.11)	1.00E-03	8.10E-02	Cervical cancer
6p22.1	HLA-G	rs3869062	G/A	0.91(0.86-0.96)	7.10E-04	6.60E-02	Nasopharyngeal carcinoma
11q12.2	FADS1	rs174549	A/G	0.90(0.87-0.94)	1.00E-07	1.40E-04	Laryngeal squamous cell carcinoma
16q23.1	RFWD3	rs7193541	C/T	0.93(0.90-0.96)	1.20E-04	1.90E-02	Multiple myeloma
17q12	HNF1B	rs8064454	C/A	1.07(1.03-1.11)	4.30E-04	5.70E-02	Prostate cancer

*EA, effect allele; NEA, non-effect allele*.

a *Based on meta-analysis of logistic regression results from 4 lung cancer GWASs*.

**Figure 3:**
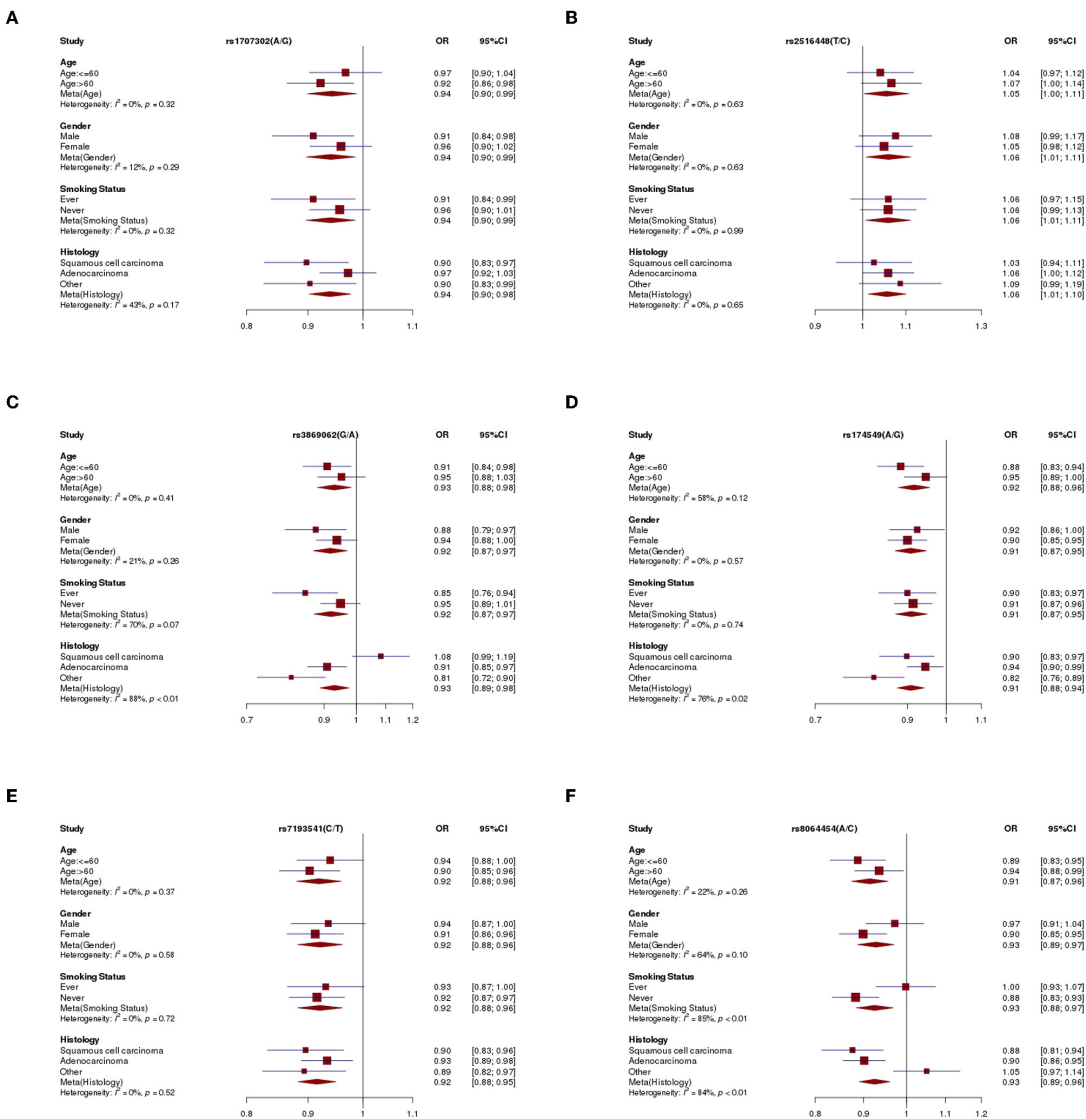
The forest plot of 6 significant SNPs. **(A)** The forest plot of rs1707302. **(B)** The forest plot of rs2516448. **(C)** The forest plot of rs3869062. **(D)** The forest plot of rs174549. **(E)** The forest plot of rs7193541. **(F)** The forest plot of rs8064454.

Subgroup analyses were conducted according to age, gender, smoking status, and tumor histology to investigate potential large differences among subgroups of population. As shown in Figure 3, the association for rs3869062 showed heterogeneous among different tumor histology (*P*_heterogeneity_ < 0.01), where the association appeared to be significant to adenocarcinoma (OR = 0.91; 95% CI: 0.85–0.97) but not squamous cell carcinoma (OR = 1.08; 95% CI: 0.99–1.19). We also identify significant heterogeneity among different tumor histology in the association for rs174549 (*P*_heterogeneity_ = 0.02), where other histological types include small cell lung cancer and large cell lung cancer show more significant association with lung cancer susceptibility (OR = 0.82; 95% CI: 0.76–0.89). Besides, rs8064454 showed a significant association with lung cancer risk in non-smokers (*P*_heterogeneity_ < 0.01, OR = 0.88, 95% CI: 0.83–0.93), while not in smokers (OR = 1.00; 95% CI: 0.93–1.07). Then, a significant interaction (*P*-interaction = 0.01, Supplementary Table 7) was observed between rs8064454 and smoking.

### Functional Annotation and Pathway Enrichment Analysis

To further unearth the underlying biological function of identified SNPs, we carried out a systematic functional annotation analysis *in silico*. The identified variants as well as their high LD (*r*^2^ > 0.6) variants were evaluated in our study. Among these variants, four were missense but none was predicted as deleterious (Supplementary Table 8). Based on results from GTEx, we found that the G allele of rs1707302 was significantly associated with increased expression of *MAST2* (β = 0.16, *P* = 4.30 × 10^−7^, Supplementary Figure 2A). Besides, rs3869062 and rs8064454 showed significant associations with up-regulated *HLA-G* (β = 0.77, *P* = 9.60 × 10^−8^, Supplementary Figure 2B) and *HNF1B* (β = 0.065, *P* = 0.028, Supplementary Figure 2C), respectively. While the protective allele of rs2516448 and rs7193541 were related to decreased expression of *MICA* (β = −0.34, *P* = 1.50 × 10^−14^, Supplementary Figure 2D) and *RFWD3* (β = −0.23, *P* = 2.20 × 10^−7^, Supplementary Figure 2E). The rs174549 was in high LD with rs174548 (*r*^2^ = 0.98), which was proven to be correlated with lung cancer risk by regulating *FADS1* gene expression in liver tissues (β = −0.23, *P* = 2.20 × 10^−7^, Supplementary Figure 2F) and plasma levels of polyunsaturated fatty acids (PUFAs) according to our recent study (22).

Gene-based analysis revealed that the associations between identified related genes and lung cancer risk were statistical significant (*P* < 0.05, Supplementary Table 9). To further explore the biological process of these identified related genes, we performed co-expressed analysis using data from GTEx V7 database and implemented pathway analysis with KEGG database. At the level of statistical significance (*P*_fdr_ < 0.05), related genes along with their co-expressed genes were significantly aggregated in cancer associated pathways, such as p53 signaling pathway (23), Ras signaling pathway (24), MAPK signaling pathway (Supplementary Table 10) (25).

## Discussion

In our study, we performed a pleiotropic analysis to explore the shared susceptibility mechanisms between non-lung cancers and lung cancer and found genetic variants identified from other cancer types were also significantly associated with the risk of lung cancer. In the single variant analysis, 6 novel susceptibility loci were identified related to lung cancer susceptibility after multiple corrections. Functional exploration revealed that these variants may modulate lung cancer risk by regulating the expression of related genes, which play important role in cancer development. Our findings demonstrate shared genetic components across cancers and provide important insight for future investigation of biological mechanisms involved in cancer progression.

We conduct a systematic scan on the susceptibility bands for each cancer and found that the majority (50/60) of lung cancer susceptibility bands are in common with that of non-lung cancers. Besides, pleiotropic analysis showed that previously reported GWAS SNPs for non-lung cancer were also associated with the risk of lung cancer, indicating shared heritability across cancers. However, for the lack of GWAS summary data of each cancer, which is necessary for estimating the correlation explained by the effects of common SNPs, we performed a SNP-set analysis based on our existing lung cancer GWAS data, and identified 8 cancer types correlated with lung cancer. Some previous studies also reported similar findings (26–29). Lindström et al. found significant genetic correlation between colorectal and lung cancer (*r*_g_ = 0.31) (26); Sampson et al. identified that bladder cancer was statistically correlated with lung cancer (*r*_g_ = 0.35) (27); Recently, Xia et al. revealed significant genetic correlation between ovarian and lung cancer (*r*_g_ = 0.18) (28). Additionally, the genetic associations based on UK Biobank demonstrated that lung cancer was genetically correlated with blood cell tumors (*r*_g_ = 0.18) and malignant neoplasms of digestive organs (*r*_g_ = 0.10) (29). Our data supported these findings, and identify another 2 cancer types (cervical and prostate cancer) correlated with lung cancer. The mechanism underlying these observations has not been well-characterized. It is likely that the correlations are due to the shared genetic factors such as penetrant mutations in *BRCA2*, which predisposes to breast, ovarian, lung, and prostate cancers (30, 31). In addition, the correlations between ovarian, colorectal and lung cancer might be driven in part by genetic variants in the inflammation pathway (32). Interestingly, bladder cancer was identified to be genetic correlated with both lung cancer and smoking (27), suggesting the overlapped smoking-related SNPs among these cancers.

Rs1707302 (1p34.1), a known risk locus of breast cancer, was associated with the risk of lung cancer in our study. SNPs in LD (*r*^2^ > 0.6) with rs1707302 mainly locate in the functional regions of *MAST2* (Supplementary Table 7), which encodes a microtubule-associated serine/threonine kinase. *MAST2* has been identified to be involved in PI3K-AKT signaling pathway (33, 34), which plays crucial role in regulating many cellular processes including cell proliferation, survival, growth and motility (35). Consistent with these findings, we found that genes co-expressed with *MAST2* were significantly enriched in cell metabolism, DNA replication and RNA polymerase pathways.

For 6p21.33, we identified that the cervical cancer susceptibility locus, rs2516448, was associated with lung cancer susceptibility. This variant was related to the expression of *MICA*, a stress-induced gene expressed by cancer cells, function as ligands for NKG2D receptors (36). Proteolytic shedding of *MICA* from tumor cells, might promote immunosubversion by reducing the expression of NKG2D (37). Besides, soluble *MICA* released by tumor cells contributes to tumor immune evasion through down-regulating NKG2D and inactiving tumor-antigen-specific effector T cells (38). Chen et al. reported that rs2516448 is in perfect LD with a frameshift mutation (A5.1) in *MICA* exon 5, which results in less membrane-bound *MICA*, causing immune inactivation and tumor progression (39). Furthermore, pathway analysis revealed an enrichment of genes co-expressed with *MICA* in Non-small cell lung cancer, Ras signaling pathway, MAPK signaling pathway, etc, indicating the important role of *MICA* in carcinogenesis process.

Two head/neck cancer GWAS SNP, rs3869062 (6p22.1) and rs174549 (11q12.2), were correlated with lung cancer susceptibility. Rs3869062 showed significant association with increased expression of *HLA-G*, which has been reported to be involved in immune recognition and might manipulate tumor specific immune responses through cytokine production (40). For rs174549, a recent metabolome-wide association study identified its high LD (*r*^2^ = 0.98) variant, rs174548, associated with both plasma levels of polyunsaturated fatty acids (PUFAs) and lung cancer risk, proposing that plasma PUFAs might function as risk indicator of lung cancer (22). However, challenge remains to unravel the molecular mechanisms underlying observations and further investigations are needed.

The multiple myeloma susceptibility locus, rs7193541, was located in the exon of *RFWD3* in 16q23.1. Functional annotation revealed that rs7193541 is a missense variant of *RFWD3* and its related SNPs (*r*^2^ > 0.6) mainly locate in the functional regions of *RFWD3*. *RFWD3* is an E3 ubiquitin ligase that involves in replication protein A (RPA) mediated DNA damage and repair (41). In addition, it has been suggested that *RFWD3* mediates the ubiquitination of p53/TP53 by forming a RFWD3-MDM2-p53 complex in the late response to DNA damage (42). Consistent with previous observations (41, 42), genes co-expressed with *RFWD3* were enriched in Cell cycle, DNA replication and p53 signaling pathway, suggesting that *RFWD3* contributes to lung cancer susceptibility possibly by manipulating DNA repair process.

The rs8064454, a previously reported susceptibility loci for prostate cancer, is located in the intron of *HNF1B* at chromosome 17q12. Rs8064454 was associated with the expression of *HNF1B* and variants in LD (*r*^2^ > 0.6) with rs8064454 all reside in a region that has a chromatin state indicative of promoter and enhancer elements. *HNF1B*, a member of the homeodomain-containing superfamily of transcription factors (TFs), has been demonstrated to act as a bookmarking factor and bind to mitotic chromatin with efficient DNA binding ability, playing crucial roles in the epigenetic transmission of information through the cell cycle (43). Thus, abnormal mitotic chromatin binding induced by mutations of *HNF1B* may be responsible for human pathological conditions. For instance, SNPs of *HNF1B* are associated with risk of multiple cancers, including prostate (44), ovarian (45), endometrial (46), and renal cell carcinomas (47). Our study found that genes co-expressed with *HNF1B* were correlated with Ras signaling pathway, EGFR tyrosine kinase inhibitor resistance, mTOR signaling pathway, etc.

In conclusion, we systematically evaluated the association of non-lung cancer susceptibility loci with lung cancer risk. We also identified 6 independent SNPs associated with the susceptibility to lung cancer. Functional exploration revealed that the related genes may be involved in cancer-associated pathways across multiple cancers. However, one limitation of our study is that we lack detailed biological mechanisms of how these SNPs are associated with different pathways and thus are involved in different cancers. In this case, molecular experiments are warranted to better characterize the effects of identified variants as well as related genes on lung cancer development.

## Data Availability Statement

SNP data is available in the FigShare database (https://figshare.com/), doi: https://dx.doi.org/10.6084/m9.figshare.11346668.

## Ethics Statement

The studies involving human participants were reviewed and approved by Ethics Committee of Nanjing Medical University. The patients/participants provided their written informed consent to participate in this study.

## Author Contributions

HS, HM, ZH, GJ, and JD: study conception and design and manuscript review. YW, JF, QS, and MJ: literature review and data extraction and quality control. LW, XF, and JX: statistical analysis. LW and MZ: manuscript preparation.

### Conflict of Interest

The authors declare that the research was conducted in the absence of any commercial or financial relationships that could be construed as a potential conflict of interest.
