# Supplementary material for: Cross-Cancer Pleiotropic Analysis Reveals Novel Susceptibility Loci for Lung Cancer
Source: Front Oncol. 2020 Jan 15;9:1492. doi: 10.3389/fonc.2019.01492 (PMC6974684; doi:10.3389/fonc.2019.01492)
Supplement: Supplementary file 1 [file Table_1.doc]

**Cross-cancer pleiotropic analysis reveals novel susceptibility loci for lung cancer**

**Supplementary Tables**

**Supplementary Table 1. 4 cancer enriched bands and GWAS loci within bands identified in non-lung cancers.**

| **Band** | **Cancer type** | **SNP** | **Report gene** | ***P***^a^ | ***P***^b^ | **SNP (R2**)**^c^** |
| --- | --- | --- | --- | --- | --- | --- |
| 5p15.33 | Esophageal cancer | **rs401681** | TERT, CLPTM1L | <1.00E-07 | 1.40E-06 | rs401681(1.000) |
|  | Pancreatic cancer | **rs31490** | CLPTM1L | 2.00E-11 | 6.20E-08 | rs467095(0.979) |
|  |  | **rs401681** | TERT, CLPTM1L | 9.00E-17 | 1.40E-06 | rs401681(1.000) |
|  |  | rs2736098 | ZDHHC11, BRD9, TRIP13, LOC100506688, NKD2, SLC12A7, MIR4635, SLC6A19, SLC6A18, MIR4457, TERT, SLC6A3, LPCAT1, CLPTM1L, SDHAP3, LOC728613, MIR4277 | 7.00E-15 | 3.60E-05 | rs2853677(0.190) |
|  |  | rs35226131 | TERT, CLPTM1L | 2.00E-08 | - | rs2736100(0.017) |
|  | Ovarian cancer | **rs7705526** | TERT | 2.00E-20 | 7.40E-23 | rs7705526(1.000) |
|  |  | **rs10069690** | TERT | 2.00E-12 | 5.80E-06 | rs2736100(0.276) |
|  | Breast cancer | **rs7726159** | TERT | 3.00E-08 | 3.60E-21 | rs7705526(0.868) |
|  |  | **rs10069690** | TERT | 2.00E-35 | 5.80E-06 | rs2736100(0.276) |
|  |  | **rs2853669** | intergenic | 4.00E-21 | 5.90E-06 | rs2853677(0.261) |
|  |  | **rs2242652** | TERT | 8.00E-28 | 7.60E-06 | rs7705526(0.251) |
|  |  | **rs3215401** | TERT | 6.00E-21 | - | rs2853677(0.254) |
|  |  | rs2736108 | TERT | 3.00E-14 | 3.30E-05 | rs2853677(0.198) |
|  | Prostate cancer | **rs2736100** | TERT | 9.00E-25 | 5.00E-25 | rs2736100(1.000) |
|  |  | **rs7725218** | TERT, hTERT | 3.00E-11 | 7.10E-21 | rs7705526(0.822) |
|  |  | **rs4635969** | TERT, CLPTM1L | 3.00E-27 | 8.60E-10 | rs4975616(0.411) |
|  |  | **rs401681** | TERT, CLPTM1L | 1.00E-10 | 1.40E-06 | rs401681(1.000) |
|  |  | **rs2242652** | TERT | 3.00E-24 | 7.60E-06 | rs7705526(0.251) |
|  |  | rs2736098 | TERT | <1.00E-07 | 3.60E-05 | rs2853677(0.190) |
|  |  | rs12653946 | intergenic | 4.00E-18 | 9.40E-02 | rs2736100(0.007) |
|  | Leukemia/Lymphoma | **rs7705526** | TERT | 6.00E-10 | 7.40E-23 | rs7705526(1.000) |
|  |  | rs924607 | CEP72 | 6.00E-09 | 5.20E-01 | rs190996050(0.011) |
|  | Glioma/Neuroblastoma | **rs2736100** | TERT | 2.00E-17 | 5.00E-25 | rs2736100(1.000) |
|  |  | **rs2735940** | TERT | <1.00E-07 | 1.60E-10 | rs2853677(0.591) |
|  |  | **rs10069690** | TERT | 8.00E-74 | 5.80E-06 | rs2736100(0.276) |
|  |  | **rs2853676** | TERT | 4.00E-14 | 8.80E-06 | rs2853677(0.311) |
|  |  | **rs72709458** | intergenic | 6.00E-24 | 5.30E-05 | rs7705526(0.322) |
|  |  | rs2736118 | TERT | <1.00E-07 | 9.90E-01 | rs380286(0.008) |
|  | Skin cancers | **rs421284** | CLPTM1L | 1.00E-18 | 1.40E-08 | rs465498(0.991) |
|  |  | **rs452932** | TERT, CLPTM1L | 2.00E-09 | 2.20E-08 | rs465498(0.985) |
|  |  | **rs401681** | TERT | 9.00E-13 | 1.40E-06 | rs401681(1.000) |
|  | Bladder/Renal cancer | **rs401681** | TERT, CLPTML | 4.00E-11 | 1.40E-06 | rs401681(1.000) |
|  |  | rs2736098 | TERT | <1.00E-07 | 3.60E-05 | rs2853677(0.190) |
|  | Head/neck cancer | **rs31489** | TERT, CLPTM1L | 6.00E-13 | 2.90E-08 | rs31489(1.000) |
|  |  | **rs401681** | TERT, CLPTM1L | 3.00E-14 | 1.40E-06 | rs401681(1.000) |
|  |  | rs10462706 | CLPTM1L | 6.00E-10 | 2.60E-01 | rs467095(0.199) |
|  | Cross cancers | **rs7725218** | TERT | 2.00E-10 | 7.10E-21 | rs7705526(0.822) |
| 6p21.32 | Esophageal cancer | rs35597309 | HLA | 1.00E-07 | 8.10E-02 | rs9272346(0.085) |
|  | Hepatocellular carcinoma | **rs9272105** | HLA-DRB1, HLA-DQA1 | 5.00E-22 | 1.30E-02 | rs28366298(0.422) |
|  |  | rs9275319 | HLA-DQ | 3.00E-17 | 4.60E-02 | rs2395185(0.158) |
|  |  | rs9275572 | HLA-DQ, HLA-DR | 6.00E-09 | 1.90E-01 | rs2395185(0.100) |
|  | Cervical/Endometrial cancer | **rs3130196** | HLA-DRB1, HLA-DQA1 | 2.30E-09 | 1.60E-02 | rs2179920(0.451) |
|  |  | **rs9272143** | HLA-DRB1, HLA-DQA1 | 9.30E-24 | 2.60E-02 | rs28366298(0.276) |
|  |  | **rs9271898** | HLA-DRB1, HLA-DQA1 | 1.20E-24 | 8.80E-02 | rs28366298(0.292) |
|  |  | rs4282438 | HLA-DPB2 | 5.00E-27 | 5.00E-07 | rs2179920(0.067) |
|  |  | rs3117027 | HLA-DPB2 | 1.00E-07 | 4.40E-03 | rs2179920(0.182) |
|  |  | rs115625939 | HLA-DRB1, HLA-DQA1 | 1.40E-15 | 4.90E-03 | rs9272346(0.162) |
|  |  | rs73730372 | intergenic | 3.00E-19 | 8.30E-03 | rs9272346(0.176) |
|  |  | rs9277952 | intergenic | 2.00E-09 | 1.40E-01 | rs2179920(0.016) |
|  | Prostate cancer | rs3096702 | CYP21A2, DOM3Z, FKBPL, HLA-DRB6, HCG23, LOC10029, ATF6B, LOC100293534, AGER, LOC100507547, MIR1236, PPT2, PPT2-EGF, HLA-DRA, PPT2-EGFL8, RNF5, RNF5P1, CFB, RDBP, TNXA, TNXB, HLA-DQB1, NOTCH4, SKIV2L, STK19, GPSM3, PBX2, LOC10050, EGFL8, AGPAT1, C2, C4A, PRRT1, BTNL2, C4B, HLA-DRB5, C6orf10, HLA-DRB1, CYP21A1P, HLA-DQA1 | 5.00E-09 | 2.00E-01 | rs3817963(0.023) |
|  | Leukemia/Lymphoma | **rs2395185** | HLA-DRA | 4.00E-31 | 2.10E-02 | rs2395185(1.000) |
|  |  | **rs9268853** | BTLN2, HLA-DRA, HLA-DRB5, HLA-DRB1 | 2.00E-10 | 3.20E-02 | rs2395185(0.996) |
|  |  | **rs6903608** | HLA-DRA | 3.00E-50 | 5.20E-02 | rs2395185(0.251) |
|  |  | **rs9269081** | HLA-DRA | 2.00E-39 | 5.30E-02 | rs2395185(0.224) |
|  |  | **rs9273363** | HLA | 2.00E-10 | 2.80E-01 | rs28366298(0.341) |
|  |  | **rs12195582** | HLA | 5.00E-100 | 3.20E-01 | rs2395185(0.543) |
|  |  | **rs2858870** | HLA-DQB1, HLA-DRB1 | 8.00E-18 | 3.50E-01 | rs28366298(0.236) |
|  |  | **rs9271176** | HLA | 3.00E-20 | 8.30E-01 | rs9272346(0.445) |
|  |  | rs9272535 | HLA-DQA1 | 9.00E-08 | 6.70E-02 | rs9272346(0.133) |
|  |  | rs9275517 | intergenic | 4.00E-11 | 1.90E-01 | rs2395185(0.100) |
|  |  | rs2647045 | HLA-DQA2, HLA-DQB1 | 4.00E-10 | 2.10E-01 | rs3817963(0.180) |
|  |  | rs674313 | HLA-DRB5 | 7.00E-09 | 3.30E-01 | rs9272346(0.140) |
|  |  | rs204999 | HLA-DQB1, HLA-DRB1 | 8.00E-18 | 3.80E-01 | rs2233956(0.192) |
|  |  | rs2621416 | HLA-DQB2, TAP2 | 2.00E-09 | 4.40E-01 | rs7383287(0.072) |
|  |  | rs4530903 | HLA-DRB5, HLA-DQA1 | 3.00E-12 | 5.10E-01 | rs9272346(0.082) |
|  |  | rs926070 | HLA | 4.00E-08 | 6.20E-01 | rs9272346(0.025) |
|  |  | rs9268528 | HLA-DQB1, HLA-DRB1 | 8.00E-18 | 6.20E-01 | rs9272346(0.170) |
|  |  | rs13196329 | C6orf10 | 3.00E-14 | 6.70E-01 | rs9272346(0.015) |
|  |  | rs9268542 | HLA-DQB1, HLA-DRB1 | 8.00E-18 | 7.50E-01 | rs9272346(0.162) |
|  |  | rs10484561 | HLA-DQB1 | 1.00E-29 | 9.20E-01 | rs9272346(0.122) |
|  |  | rs2647012 | HLA-DQB1 | 2.00E-21 | 9.60E-01 | rs2395185(0.145) |
|  |  | rs9461741 | BTNL2 | 4.00E-15 | - | rs2395185(0.018) |
|  | Skin cancers | rs4455710 | HLA-DQA1 | 2.00E-18 | 1.70E-01 | rs28366298(0.103) |
|  |  | rs9275642 | HLA-DQA2 | 2.00E-12 | 5.40E-01 | rs3817963(0.121) |
|  | Head/neck cancer | **rs3828805** | HLA-DQB1 | 3.00E-13 | 5.00E-01 | rs9272346(0.202) |
|  |  | rs28421666 | HLA-DQ, HLA-DR | 2.00E-18 | 2.50E-01 | rs2395185(0.038) |
|  |  | rs3129780 | intergenic | 6.00E-08 | 4.30E-01 | rs9272346(0.157) |
|  | Other cancers | **rs1800625** | AGER | 8.00E-09 | 6.80E-03 | rs3131378(0.243) |
| 8q24.21 | Pancreatic cancer | **rs1561927** | LINC00977, MIR1208, PVT1, MYC | 7.00E-08 | 4.00E-02 | rs67824503(0.684) |
|  |  | rs10094872 | MYC | 1.00E-09 | 1.00E-01 | rs67824503(0.05) |
|  | Colorectal Cancer | rs10505477 | LOC101930033, CCAT2 | 3.00E-22 | 3.80E-01 | rs67824503(0.003) |
|  |  | rs2128382 | intergenic | 4.00E-08 | 3.90E-01 | rs67824503(0.004) |
|  |  | rs10808555 | CASC8 | <1.00E-07 | 4.10E-01 | rs67824503(0.001) |
|  |  | rs12682374 | CASC8 | 7.00E-29 | 4.10E-01 | rs67824503(0.003) |
|  |  | rs6983267 | POU5F1B, CCAT2, LOC101930033,MYC | 8.00E-28 | 5.00E-01 | rs67824503(0.005) |
|  |  | rs10808556 | CASC8 | 5.10E-08 | 5.60E-01 | rs67824503(<0.001) |
|  |  | rs7837328 | CASC8 | <1.00E-07 | 6.10E-01 | rs67824503(<0.001) |
|  |  | rs7014346 | POU5FIP1, HsG57825, DQ515897 | 9.00E-26 | 6.30E-01 | rs67824503(0.001) |
|  | Ovarian cancer | **rs10088218** | CMYC | 1.00E-20 | 1.70E-02 | rs67824503(0.273) |
|  |  | **rs1400482** | intergenic | 7.00E-26 | 1.80E-02 | rs67824503(0.273) |
|  |  | **rs7814937** | LINC00824 | <1.00E-07 | 2.00E-02 | rs67824503(0.273) |
|  |  | **rs10098821** | LINC00824 | <1.00E-07 | - | rs67824503(0.344) |
|  |  | rs10808556 | POU5F1P1 | <1.00E-07 | 5.60E-01 | rs67824503(<0.001) |
|  |  | rs1516982 | LINC00824 | <1.00E-07 | 6.20E-01 | rs67824503(0.159) |
|  |  | rs9886651 | PVT1 | 2.00E-09 | 9.30E-01 | rs67824503(0.034) |
|  | Breast cancer | rs13281615 | POU5F1B, FAM84B, MYC | 2.00E-57 | 6.80E-01 | rs67824503(0.002) |
|  |  | rs7017073 | intergenic | 2.00E-14 | 1.20E-01 | rs67824503(0.012) |
|  |  | rs11780156 | MYC, MIR1208 | 1.00E-13 | 1.80E-01 | rs67824503(<0.001) |
|  |  | rs1562430 | POU5F1B, MYC | 5.00E-12 | 4.70E-01 | rs67824503(0.027) |
|  |  | rs10096351 | intergenic | 2.00E-64 | 4.90E-01 | rs67824503(0.026) |
|  |  | rs2392780 | intergenic | 1.00E-08 | 5.30E-01 | rs67824503(0.012) |
|  | Prostate cancer | rs12543663 | PCAT1 | <1.00E-07 | 6.30E-01 | rs67824503(0.056) |
|  |  | rs7000448 | POU5F1B | <1.00E-07 | 5.40E-02 | rs67824503(0.012) |
|  |  | rs4506170 | CASC8, CASC21 | 2.00E-09 | 6.60E-02 | rs67824503(0.008) |
|  |  | rs16902094 | intergenic | 1.00E-21 | 7.60E-02 | rs67824503(0.010) |
|  |  | rs4871008 | PCAT2 | 6.20E-10 | 2.00E-01 | rs67824503(0.001) |
|  |  | rs13252298 | PCAT1 | 4.10E-10 | 2.30E-01 | rs67824503(0.003) |
|  |  | rs1551510 | intergenic | 3.00E-15 | 2.80E-01 | rs67824503(0.002) |
|  |  | rs1456306 | intergenic | 3.00E-15 | 3.00E-01 | rs67824503(0.001) |
|  |  | rs72725879 | intergenic | 3.00E-08 | 3.20E-01 | rs67824503(0.084) |
|  |  | rs7463708 | PRNCR1 | 6.90E-10 | 3.70E-01 | rs67824503(0.054) |
|  |  | rs10505477 | MYC | 7.00E-21 | 3.80E-01 | rs67824503(<0.001) |
|  |  | rs6994316 | intergenic | 3.00E-15 | 3.90E-01 | rs67824503(0.031) |
|  |  | rs7816535 | intergenic | 3.00E-15 | 3.90E-01 | rs67824503(0.031) |
|  |  | rs7825414 | intergenic | 3.00E-15 | 4.00E-01 | rs67824503(0.093) |
|  |  | rs1456315 | intergenic | 2.00E-29 | 4.30E-01 | rs67824503(0.042) |
|  |  | rs17831626 | intergenic | 3.00E-15 | 4.70E-01 | rs67824503(0.080) |
|  |  | rs13254738 | intergenic | 4.00E-10 | 4.70E-01 | rs67824503(0.040) |
|  |  | rs7013255 | intergenic | 4.20E-48 | 5.00E-01 | rs67824503(0.037) |
|  |  | rs6983267 | SRRM1P1, POU5F1B | 3.00E-27 | 5.00E-01 | rs67824503(<0.001) |
|  |  | rs1016342 | intergenic | 3.00E-15 | 5.30E-01 | rs67824503(0.006) |
|  |  | rs16901979 | POU5F1B | 1.00E-20 | 5.30E-01 | rs67824503(0.037) |
|  |  | rs10505483 | intergenic | 7.00E-15 | 5.50E-01 | rs67824503(0.037) |
|  |  | rs10808556 | CASC8 | 4.20E-08 | 5.60E-01 | rs67824503(<0.001) |
|  |  | rs1378897 | intergenic | 3.00E-15 | 5.70E-01 | rs67824503(0.018) |
|  |  | rs7837328 | CASC8 | <1.00E-07 | 6.10E-01 | rs67824503(<0.001) |
|  |  | rs1456305 | intergenic | 3.00E-15 | 6.30E-01 | rs67824503(0.027) |
|  |  | rs7841060 | intergenic | 1.50E-13 | 6.60E-01 | rs67824503(0.003) |
|  |  | rs12682344 | SRRM1P1, POU5F1B | 5.00E-12 | 6.80E-01 | rs67824503(0.037) |
|  |  | rs1016343 | intergenic | 5.00E-21 | 6.90E-01 | rs67824503(0.003) |
|  |  | rs6983561 | PCAT1 | 6.00E-22 | 7.10E-01 | rs67824503(0.037) |
|  |  | rs10099413 | intergenic | 1.20E-08 | 7.10E-01 | rs67824503(0.007) |
|  |  | rs56005245 | PRNCR1 | 1.00E-08 | 7.20E-01 | rs67824503(0.002) |
|  |  | rs7017300 | intergenic | <1.00E-07 | 7.50E-01 | rs67824503(0.007) |
|  |  | rs7812894 | intergenic | 1.90E-10 | 8.00E-01 | rs67824503(<0.001) |
|  |  | rs4242384 | POU5F1P1 | 2.00E-24 | 8.20E-01 | rs67824503(<0.001) |
|  |  | rs7812429 | intergenic | 1.90E-10 | 8.30E-01 | rs67824503(<0.001) |
|  |  | rs7814837 | intergenic | 1.90E-10 | 8.60E-01 | rs67824503(<0.001) |
|  |  | rs1456314 | intergenic | 3.00E-15 | 9.00E-01 | rs67824503(<0.001) |
|  |  | rs7837688 | intergenic | 1.00E-25 | 9.00E-01 | rs67824503(0.001) |
|  |  | rs11986220 | LOC727677 | 4.00E-45 | 9.10E-01 | rs67824503(0.001) |
|  |  | rs13255059 | intergenic | 5.20E-69 | 9.10E-01 | rs67824503(0.001) |
|  |  | rs10086908 | PCAT1 | 1.00E-10 | 9.20E-01 | rs67824503(0.010) |
|  |  | rs10090154 | intergenic | 6.80E-24 | 9.20E-01 | rs67824503(0.001) |
|  |  | rs445114 | POU5F1B | 5.00E-10 | 9.30E-01 | rs67824503(0.003) |
|  |  | rs6993569 | intergenic | 3.00E-15 | 9.40E-01 | rs67824503(0.032) |
|  |  | rs6470494 | intergenic | 3.00E-15 | 9.60E-01 | rs67824503(0.001) |
|  |  | rs1031588 | intergenic | 3.00E-15 | 9.60E-01 | rs67824503(0.055) |
|  |  | rs620861 | intergenic | 1.00E-22 | 9.60E-01 | rs67824503(0.002) |
|  |  | rs1447295 | MYC, LOC727677, POU5F1B | 1.00E-24 | 9.80E-01 | rs67824503(0.001) |
|  |  | rs4242382 | LOC727677, CASC8, POU5F1P1 | 1.00E-34 | 9.80E-01 | rs67824503(0.001) |
|  |  | rs979200 | LOC105375751 | <1.00E-07 | 9.90E-01 | rs67824503(0.009) |
|  |  | rs77541621 | intergenic | 2.00E-26 | - | rs67824503(0.004) |
|  |  | rs116041037 | PCAT1 | 6.00E-10 | - | - |
|  |  | rs188140481 | HOXB13 | 6.00E-34 | - | rs67824503(0.006) |
|  |  | rs138042437 | CASC19 | 2.00E-08 | - | rs67824503(0.004) |
|  | Leukemia/Lymphoma | rs2456449 | POU5F1P1 | 7.80E-10 | 5.80E-01 | rs67824503(0.014) |
|  |  | rs13254990 | PVT1 | 1.00E-08 | 1.80E-02 | rs67824503(0.014) |
|  |  | rs13255292 | MYC, PVT1 | 1.00E-12 | 3.20E-02 | rs67824503(0.016) |
|  |  | rs4617118 | intergenic | 2.00E-12 | 3.20E-02 | rs67824503(0.012) |
|  |  | rs4733601 | MYC, PVT1 | 4.00E-11 | 6.40E-02 | rs67824503(0.114) |
|  |  | rs2720680 | PVT1 | 7.00E-08 | 1.20E-01 | rs67824503(0.001) |
|  |  | rs1948915 | CCAT1 | 4.00E-11 | 1.80E-01 | rs67824503(0.029) |
|  |  | rs2466035 | MYC | 2.00E-08 | 4.00E-01 | rs67824503(0.013) |
|  |  | rs2019960 | PVT1 | 1.00E-13 | 6.10E-01 | rs67824503(0.014) |
|  |  | rs2466029 | POU5F1B | 7.00E-16 | 6.70E-01 | rs67824503(0.010) |
|  |  | rs2608053 | PVT1 | 1.00E-07 | 7.80E-01 | rs67824503(0.020) |
|  | Glioma/Neuroblastoma | rs10464870 | CCDC26 | <1.00E-07 | 1.30E-02 | rs67824503(0.003) |
|  |  | rs891835 | CCDC26 | 8.00E-11 | 6.00E-02 | rs67824503(0.013) |
|  |  | rs4295627 | CCDC26 | 5.00E-21 | 1.20E-01 | rs67824503(0.001) |
|  |  | rs16904140 | CCDC26 | <1.00E-07 | 1.50E-01 | rs67824503(<0.001) |
|  |  | rs6470745 | CCDC26 | <1.00E-07 | 1.70E-01 | rs67824503(0.004) |
|  |  | rs9656979 | intergenic | <1.00E-07 | 4.90E-01 | rs67824503(0.010) |
|  |  | rs55705857 | CCDC26 | 7.00E-149 | - | rs67824503(0.030) |
|  | Bladder/Renal cancer | rs6470589 | MYC, PVT1 | 5.00E-11 | 2.40E-02 | rs67824503(<0.001) |
|  |  | rs6470588 | MYC, PVT1 | 5.00E-11 | 2.60E-02 | rs67824503(<0.001) |
|  |  | rs9642880 | MYC, BC042052 | 4.00E-38 | 4.30E-01 | rs67824503(0.020) |
|  | Cervical/Endometrial cancer | **rs4733613** | MYC | 3.00E-09 | - | rs67824503(0.239) |
| 9p21.3 | Ovarian cancer | rs3731257 | CDKN2A | <1.00E-07 | 6.70E-01 | rs1333040(0.059) |
|  | Breast cancer | **rs10811650** | intergenic | 2.00E-13 | 6.50E-01 | rs1333040(0.268) |
|  |  | **rs1011970** | CDKN2A, CDKN2B | 1.00E-15 | 6.70E-01 | rs62560775(0.590) |
|  | Prostate cancer | rs17694493 | CDKN2B-AS1 | 4.00E-08 | 4.30E-01 | rs1333040(0.017) |
|  | Leukemia/Lymphoma | rs3731217 | CDKN2A, CDKN2B | 2.00E-08 | 2.00E-01 | rs1333040(0.067) |
|  |  | rs2811710 | CDKN2A | 2.00E-13 | 1.60E-01 | rs62560775(0.151) |
|  |  | rs2069426 | CDKN2A | 4.00E-09 | 4.50E-01 | rs885518(0.011) |
|  |  | rs1359742 | DMRTA1, CDKN2B-AS1 | 7.00E-09 | 6.80E-01 | rs885518(0.022) |
|  |  | rs1679013 | AS1, CDKN2B | 1.00E-08 | 9.00E-01 | rs1333040(0.020) |
|  | Glioma/Neuroblastoma | **rs1063192** | CDKN2B | <1.00E-07 | 5.00E-01 | rs1333040(0.312) |
|  |  | **rs4977756** | CDKN2A, CDKN2B | 7.00E-15 | 2.00E-01 | rs1333040(0.416) |
|  |  | **rs2157719** | CDKN2A, CDKN2B | 3.00E-17 | 5.60E-01 | rs1333040(0.244) |
|  |  | **rs1412829** | CDKN2A, CDKN2B | 2.00E-10 | 6.50E-01 | rs1333040(0.236) |
|  |  | **rs634537** | CDKN2A, CDKN2B | 1.00E-45 | 7.50E-01 | rs1333040(0.238) |
|  |  | **rs145929329** | intergenic | 5.00E-12 | - | rs1333040(0.222) |
|  |  | rs545226 | CDKN2B | <1.00E-07 | 5.00E-01 | rs1333040(0.091) |
|  | Skin cancers | rs1335510 | MTAP | 5.90E-09 | 4.30E-02 | rs885518(0.128) |
|  |  | rs10757257 | MTAP | 3.00E-11 | 8.00E-02 | rs885518(0.133) |
|  |  | rs7023329 | MTAP | 1.10E-25 | 4.60E-01 | rs885518(0.037) |
|  |  | rs7874604 | CDKN2B | 5.00E-13 | 7.50E-01 | rs1333040(0.122) |
|  |  | rs2218220 | MTAP | 5.50E-11 | 7.90E-01 | rs885518(0.154) |
|  |  | rs2151280 | CDKN2A, CDKN2B | 9.00E-11 | 8.10E-01 | rs1333040(0.106) |
|  |  | rs4636294 | MTAP | <1.00E-07 | 9.30E-01 | rs885518(0.153) |
|  | Head/neck cancer | **rs1412829** | CDKN2A, CDKN2B | 3.00E-08 | 6.50E-01 | rs1333040(0.236) |
|  |  | **rs8181047** | CDKN2B-AS1 | 4.00E-09 | 8.20E-01 | rs1333040(0.440) |

Note: Bold fonts represent lung cancer GWAS loci as well as those in the same LD blocks (r^2^ > 0.2).

^a^ P value in the genome-wide association studies of specific cancers, the most significant one is shown.

^b^ P value in the meta-analysis of logistic regression results from 4 lung cancer GWASs.

^c^ Lung cancer GWAS loci and their LD relationships with non-lung cancer GWAS SNPs.

**Supplementary Table 2. Correlations between non-lung cancers and lung cancer by SNP-set analysis.**

| **Cancer type** | **Number of tested SNPs** | ***P_NJMU_*^a^** | ***P_FLCCA_*^b^** | ***P_EAGLE_*^c^** | ***P_DCEG_*^d^** | ***P_combined_*^e^** | **FDR^e^** |
| --- | --- | --- | --- | --- | --- | --- | --- |
| **Cervical/Endometrial cancer** | 21 | 1.80E-07 | 1.80E-02 | 8.30E-01 | 6.10E-03 | 1.30E-07 | 2.10E-06 |
| **Bladder/Renal cancer** | 45 | 8.50E-06 | 1.40E-02 | 9.20E-01 | 2.60E-02 | 2.20E-05 | 1.70E-04 |
| **Prostate cancer** | 266 | 2.30E-02 | 4.50E-01 | 4.40E-01 | 8.20E-02 | 2.50E-03 | 1.20E-02 |
| **Pancreatic cancer** | 34 | 1.60E-01 | 7.10E-02 | 6.90E-01 | 3.50E-02 | 3.00E-03 | 1.20E-02 |
| **Ovarian cancer** | 57 | 8.30E-08 | 5.70E-01 | 6.40E-01 | 2.00E-02 | 3.90E-03 | 1.30E-02 |
| **Leukemia/Lymphoma** | 193 | 5.80E-04 | 3.90E-01 | 3.00E-02 | 5.80E-01 | 5.40E-03 | 1.50E-02 |
| **Esophageal cancer** | 41 | 1.20E-05 | 5.40E-01 | 7.70E-01 | 1.60E-01 | 1.10E-02 | 2.50E-02 |
| **Colorectal Cancer** | 126 | 2.60E-04 | 6.60E-01 | 5.40E-01 | 1.50E-01 | 1.80E-02 | 3.60E-02 |
| Breast cancer | 388 | 1.40E-05 | 3.60E-02 | 2.30E-01 | 3.50E-01 | 4.60E-02 | 8.20E-02 |
| Glioma/Neuroblastoma | 99 | 1.90E-01 | 2.30E-01 | 6.00E-01 | 1.30E-01 | 7.10E-02 | 1.00E-01 |
| Head/neck cancer | 64 | 2.10E-02 | 3.20E-01 | 4.90E-01 | 2.90E-01 | 1.10E-01 | 1.50E-01 |
| Gastric cancer | 25 | 1.60E-01 | 2.80E-01 | 7.10E-01 | 1.10E-01 | 1.60E-01 | 1.80E-01 |
| Skin cancers | 61 | 1.30E-01 | 5.70E-02 | 8.30E-01 | 8.20E-01 | 1.40E-01 | 1.80E-01 |
| Hepatocellular carcinoma | 10 | 6.70E-01 | 2.10E-01 | 7.70E-01 | 5.70E-01 | 2.80E-01 | 3.00E-01 |
| Cross cancers^f^ | 20 | 4.90E-01 | 5.70E-01 | 3.80E-01 | 2.60E-02 | 5.60E-02 | 9.00E-02 |
| Other cancers | 8 | 2.10E-01 | 7.50E-01 | 9.80E-01 | 1.50E-01 | 5.40E-01 | 5.40E-01 |

Note: Bold fonts represent significant cancer types in the combined dataset (FDR<0.05).

^a^ Adjusted by age, gender, smoking pack years and the first PCA.

^b^ Adjusted by age and 3 significant PCAs.

^c^ Adjusted by age, gender, smoking status and the first PCA.

^d^ Adjusted by age, gender and the first PCA.

^e^ Adjusted by age, gender, study and the first 10 PCAs, P value and false discovery rate in the combined dataset.

^f^ Cross-cancer GWAS analysis of multiple cancers including lung, ovarian, breast, prostate and digestive system cancers using a subset-based meta-analysis.

**Supplementary Table 3. Correlations between non-lung cancers and lung cancer after excluding variants within MHC and cancer enriched bands.**

| **Cancer type** | **Number of tested SNPs** | ***P_NJMU_*^a^** | ***P_FLCCA_*^b^** | ***P***EAGLE^c^ | ***P***DCEG^d^ | ***P***combined^e^ | **FDR**^e^ |
| --- | --- | --- | --- | --- | --- | --- | --- |
| **Cervical/Endometrial cancer** | 13 | 5.20E-01 | 4.90E-02 | 6.70E-01 | 8.00E-03 | 8.50E-04 | 6.80E-03 |
| **Bladder/Renal cancer** | 41 | 5.10E-06 | 4.90E-01 | 9.50E-01 | 2.30E-02 | 2.20E-03 | 1.20E-02 |
| **Prostate cancer** | 210 | 3.10E-03 | 3.00E-01 | 4.60E-01 | 7.30E-02 | 3.30E-04 | 5.20E-03 |
| Pancreatic cancer | 32 | 1.40E-01 | 7.00E-01 | 7.60E-01 | 3.00E-02 | 1.10E-01 | 1.70E-01 |
| **Ovarian cancer** | 52 | 2.30E-08 | 6.60E-01 | 6.40E-01 | 2.00E-02 | 3.20E-03 | 1.30E-02 |
| **Leukemia/Lymphoma** | 158 | 7.80E-03 | 6.20E-01 | 2.40E-02 | 4.80E-01 | 6.60E-03 | 2.10E-02 |
| **Esophageal cancer** | 40 | 1.00E-05 | 5.40E-01 | 8.90E-01 | 1.50E-01 | 1.30E-02 | 3.10E-02 |
| **Colorectal Cancer** | 118 | 3.80E-05 | 6.20E-01 | 5.40E-01 | 1.50E-01 | 1.40E-02 | 3.10E-02 |
| Breast cancer | 381 | 1.20E-05 | 6.30E-02 | 2.40E-01 | 3.60E-01 | 8.70E-02 | 1.50E-01 |
| Glioma/Neuroblastoma | 91 | 1.40E-01 | 2.50E-01 | 6.20E-01 | 1.20E-01 | 1.30E-01 | 1.70E-01 |
| Head/neck cancer | 59 | 6.60E-02 | 4.40E-01 | 5.60E-01 | 3.80E-01 | 1.30E-01 | 1.70E-01 |
| Gastric cancer | 25 | 1.60E-01 | 2.80E-01 | 7.10E-01 | 1.10E-01 | 1.60E-01 | 1.90E-01 |
| Skin cancers | 50 | 2.10E-01 | 2.20E-01 | 9.30E-01 | 9.10E-01 | 2.80E-01 | 3.20E-01 |
| Hepatocellular carcinoma | 8 | 4.80E-01 | 6.40E-01 | 7.60E-01 | 6.10E-01 | 5.70E-01 | 5.70E-01 |
| Cross cancers^f^ | 20 | 4.90E-01 | 5.70E-01 | 3.80E-01 | 2.60E-02 | 5.60E-02 | 1.10E-01 |
| Other cancers | 8 | 2.10E-01 | 7.50E-01 | 9.80E-01 | 1.50E-01 | 5.40E-01 | 5.70E-01 |

Note: Bold fonts represent significant cancer types in the combined dataset (FDR<0.05).

^a^ Adjusted by age, gender, smoking pack years and the first PCA.

^b^ Adjusted by age and 3 significant PCAs.

^c^ Adjusted by age, gender, smoking status and the first PCA.

^d^ Adjusted by age, gender and the first PCA.

^e^ Adjusted by age, gender, study and the first 10 PCAs, P value and false discovery rate in the combined dataset.

^f^ Cross-cancer GWAS analysis of multiple cancers including lung, ovarian, breast, prostate and digestive system cancers using a subset-based meta-analysis.

**Supplementary Table 4. Associations of GWAS loci identified in non-lung cancers with lung cancer risk.**

| **Band** | **Related gene** | **SNP** | **EA/NEA** | **OR (95%CI)^a^** | ***P*^a^** | **FDR^a^** | **Source of Cancer** | **R2^b^** |
| --- | --- | --- | --- | --- | --- | --- | --- | --- |
| 1p34.1 | MAST2 | rs1707302 | A/G | 0.93(0.90-0.97) | 7.60E-04 | 6.60E-02 | Breast cancer | - |
| 6p21.33 | MICA | rs2516448 | T/C | 1.07(1.03-1.11) | 1.00E-03 | 8.10E-02 | Cervical cancer | - |
| 6p22.1 | HLA-G | rs3869062 | G/A | 0.91(0.86-0.96) | 7.10E-04 | 6.60E-02 | Nasopharyngeal  carcinoma | - |
| 11q12.2 | FADS1 | rs174549 | A/G | 0.90(0.87-0.94) | 1.00E-07 | 1.40E-04 | Laryngeal squamous cell carcinoma | - |
|  |  | rs4246215 | T/G | 0.91(0.88-0.94) | 9.10E-07 | 6.30E-04 | Colorectal Cancer | 0.853 |
|  |  | rs174550 | C/T | 0.91(0.88-0.95) | 1.80E-06 | 6.30E-04 | Colorectal Cancer | 0.894 |
|  |  | rs174537 | T/G | 0.91(0.88-0.95) | 1.90E-06 | 6.30E-04 | Colorectal Cancer | 0.890 |
|  |  | rs174594 | C/A | 0.91(0.88-0.95) | 2.40E-06 | 6.30E-04 | Colorectal cancer | 0.807 |
|  |  | rs1535 | G/A | 0.91(0.88-0.95) | 2.70E-06 | 6.30E-04 | Colorectal Cancer | 0.879 |
| 16q23.1 | RFWD3 | rs7193541 | C/T | 0.93(0.90-0.96) | 1.20E-04 | 1.90E-02 | Multiple myeloma | - |
| 17q12 | HNF1B | rs8064454 | C/A | 1.07(1.03-1.11) | 4.30E-04 | 5.70E-02 | Prostate cancer | - |
|  |  | rs11651052 | G/A | 1.07(1.03-1.11) | 4.50E-04 | 5.70E-02 | Prostate cancer,  Endometrial cancer | 0.986 |
|  |  |  |  |  |  |  |  |  |
|  |  | rs7405696 | C/G | 1.07(1.03-1.11) | 5.20E-04 | 5.80E-02 | Prostate cancer | 0.630 |
|  |  | rs11263763 | A/G | 1.07(1.03-1.11) | 5.40E-04 | 5.80E-02 | Prostate cancer,  Endometrial cancer | 0.956 |
|  |  |  |  |  |  |  |  |  |
|  |  | rs7501939 | T/C | 0.93(0.90-0.97) | 6.20E-04 | 6.20E-02 | Prostate cancer,  Testicular germ cell tumor | 0.809 |
|  |  |  |  |  |  |  |  |  |
|  |  | rs12601991 | T/G | 1.07(1.03-1.11) | 8.10E-04 | 6.60E-02 | Prostate cancer,  Ovarian cancer | 0.643 |
|  |  |  |  |  |  |  |  |  |
|  |  | rs11651755 | T/C | 1.07(1.03-1.11) | 1.10E-03 | 8.20E-02 | Ovarian cancer | 0.949 |

BP: base position; EA: effect allele; NEA: non effect allele.

^a^ Based on meta-analysis of logistic regression results from 4 lung cancer GWASs.

^b^ LD relationship with the most significant SNP in the same LD block.

| **SNP** | **EA/ NEA** | **NJMU** | | **FLCCA** | | | **EAGLE** | | **DCEG** | | **Combined** | | **I**^2^ |
| --- | --- | --- | --- | --- | --- | --- | --- | --- | --- | --- | --- | --- | --- |
|  |  | **OR (95%CI)** | **P value** | | **OR (95%CI)** | **P value** | **OR (95%CI)** | **P value** | **OR (95%CI)** | **P value** | **OR (95%CI)** | **P value** |  |
| rs1707302 | A/G | 0.90(0.82-0.99) | 2.40E-02 | | 0.96(0.90-1.03) | 2.50E-01 | 0.96(0.87-1.07) | 5.00E-01 | 0.92(0.85-0.98) | 1.20E-02 | 0.93(0.90-0.97) | 7.60E-04 | 0 |
| rs2516448 | T/C | 1.15(1.04-1.26) | 5.80E-03 | | 1.04(0.96-1.11) | 3.40E-01 | 1.08(0.96-1.20) | 1.90E-01 | 1.06(0.99-1.13) | 7.10E-02 | 1.07(1.03-1.11) | 1.00E-03 | 0 |
| rs3869062 | G/A | 0.83(0.75-0.92) | 4.20E-04 | | 0.90(0.83-0.97) | 6.70E-03 | 1.10(0.91-1.33) | 3.40E-01 | 1.02(0.88-1.18) | 7.60E-01 | 0.91(0.86-0.96) | 7.10E-04 | 67.7% |
| rs174549 | A/G | 0.83(0.76-0.90) | 1.20E-05 | | 0.91(0.85-0.97) | 4.60E-03 | 0.93(0.83-1.04) | 2.00E-01 | 0.93(0.86-0.99) | 3.20E-02 | 0.90(0.87-0.94) | 1.00E-07 | 40.5% |
| rs7193541 | C/T | 0.92(0.85-1.01) | 7.40E-02 | | 0.92(0.86-0.98) | 8.30E-03 | 0.97(0.88-1.06) | 4.90E-01 | 0.93(0.87-0.99) | 2.60E-02 | 0.93(0.90-0.96) | 1.20E-04 | 0 |
| rs8064454 | C/A | 1.03(0.94-1.13) | 5.00E-01 | | 1.07(1.00-1.15) | 4.10E-02 | 1.03(0.94-1.13) | 5.10E-01 | 1.11(1.04-1.19) | 1.80E-03 | 1.07(1.03-1.11) | 4.30E-04 | 0 |

**Supplementary Table 5. Independent associations of significant locus with lung cancer risk in each cohort.**

EA: effect allele; NEA: non effect allele.

**Supplementary Table 6. LD between 2 independent susceptibility loci and lung cancer GWAS loci in known bands.**

| **Band** | **SNP_A^a^** | **BP_A^a^** | **SNP_B^b^** | **BP_B^b^** | **R2** |
| --- | --- | --- | --- | --- | --- |
| 6p21.33 | rs2516448 | 31390410 | rs2233956 | 31081205 | 0.0460 |
|  | rs2516448 | 31390410 | rs3094222 | 31081434 | 0.0930 |
|  | rs2516448 | 31390410 | rs2596500 | 31321267 | 0.1100 |
|  | rs2516448 | 31390410 | rs1800628 | 31546850 | 0.1100 |
|  | rs2516448 | 31390410 | rs3117582 | 31620520 | 0.0860 |
|  | rs2516448 | 31390410 | rs3131379 | 31721033 | 0.0890 |
|  | rs2516448 | 31390410 | rs3131378 | 31725285 | 0.0890 |
|  | rs2516448 | 31390410 | rs501942 | 31840477 | 0.0920 |
|  | rs2516448 | 31390410 | rs3817963 | 32368087 | 0.0061 |
|  | rs2516448 | 31390410 | rs2395185 | 32433167 | 0.0095 |
|  | rs2516448 | 31390410 | rs28366298 | 32560859 | 0.0089 |
|  | rs2516448 | 31390410 | rs9272346 | 32604372 | 0.0091 |
|  | rs2516448 | 31390410 | rs7383287 | 32783086 | 0.0098 |
|  | rs2516448 | 31390410 | rs2179920 | 33058874 | 0.0010 |
| 6p22.1 | rs3869062 | 29934891 | rs149949 | 28011516 | 0.0006 |
|  | rs3869062 | 29934891 | rs34662244 | 28073881 | 0.0120 |
|  | rs3869062 | 29934891 | rs35952432 | 28074901 | 0.0120 |
|  | rs3869062 | 29934891 | rs34878803 | 28250179 | 0.0120 |
|  | rs3869062 | 29934891 | rs34661125 | 28281894 | 0.0120 |
|  | rs3869062 | 29934891 | rs67340775 | 28304384 | 0.0120 |
|  | rs3869062 | 29934891 | rs13213986 | 28358009 | 0.0120 |
|  | rs3869062 | 29934891 | rs13201681 | 28394680 | 0.0120 |
|  | rs3869062 | 29934891 | rs67381177 | 28411941 | 0.0120 |
|  | rs3869062 | 29934891 | rs4324798 | 28776117 | 0.0120 |
|  | rs3869062 | 29934891 | rs148696809 | 28934352 | 0.0120 |

BP: base position.

^a^ Two novel susceptibility loci identified in this study and their base positions.

^b^ SNPs in LD (r^2^>0.2) with SNP_A and the corresponding base positions.

**Supplementary Table 7. Interaction of rs8064454 and smoking.**

|  | **Subgroup** | **Case** | **Control** | **OR(95% CI)** | ***P*^a^** |
| --- | --- | --- | --- | --- | --- |
| **Dominant model** | G^-^ E^-^ | 3065 | 3004 | 1.00 | reference |
|  | G^-^ E^+^ | 1188 | 980 | 2.87(2.51-3.28) | 2.10E-54 |
|  | G^+^ E^-^ | 2694 | 3141 | 0.87(0.81-0.93) | 1.20E-04 |
|  | G^+^ E^+^ | 2117 | 1667 | 1.14(1.00-1.30) | 4.50E-02 |
| **Recessive model** | G^-^ E^-^ | 5318 | 5531 | 1.00 | reference |
|  | G^-^ E^+^ | 2719 | 2193 | 3.01(2.70-3.36) | 6.30E-86 |
|  | G^+^ E^-^ | 441 | 614 | 0.78(0.68-0.89) | 1.80E-04 |
|  | G^+^ E^+^ | 586 | 464 | 1.25(1.04-1.51) | 2.00E-02 |
| **Additive model** | nonsmoker | 5759 | 6145 | 0.88(0.83-0.93) | 4.20E-06 |
|  | smoker | 3305 | 2657 | 2.86(2.51-3.25) | 2.50E-58 |
|  | G×E | 9064 | 8802 | 1.13(1.03-1.24) | 1.00E-02 |

a Adjusted by age, gender.

G: Gene

E: Environment

**Supplementary Table 8. Functional annotations for significant loci and their related SNPs (r^2^>0.6).**

| **SNP** | **CHR** | **BP** | **R2^a^** | **Position** | **Gene** | **H3K27AC^b^** | **H3K4ME1^b^** | **H3K4ME3^b^** | **DHS^b^** | **TFBS^b^** | **Exonic mutation** | **RegulomeDB score** | **SIFT score** | **SIFT pred** | **PolyPhen2 score** | **PolyPhen2 pred** |
| --- | --- | --- | --- | --- | --- | --- | --- | --- | --- | --- | --- | --- | --- | --- | --- | --- |
| rs11211182 | 1 | 46240170 | 0.64 | intergenic | IPP(dist=23685),MAST2(dist=29115) | - | - | - | - | - | - | 6 | - | - | - | - |
| rs10789472 | 1 | 46247886 | 0.64 | intergenic | IPP(dist=31401),MAST2(dist=21399) | - | - | - | - | - | - | 7 | - | - | - | - |
| rs10749857 | 1 | 46248143 | 0.64 | intergenic | IPP(dist=31658),MAST2(dist=21142) | - | - | - | - | - | - | 7 | - | - | - | - |
| rs11211184 | 1 | 46249387 | 0.64 | intergenic | IPP(dist=32902),MAST2(dist=19898) | - | - | - | - | - | - | 7 | - | - | - | - |
| rs4275489 | 1 | 46250646 | 0.64 | intergenic | IPP(dist=34161),MAST2(dist=18639) | - | - | - | - | - | - | 2b | - | - | - | - |
| rs4607936 | 1 | 46250925 | 0.64 | intergenic | IPP(dist=34440),MAST2(dist=18360) | - | - | - | - | - | - | 5 | - | - | - | - |
| rs34835041 | 1 | 46251197 | 0.64 | intergenic | IPP(dist=34712),MAST2(dist=18088) | - | - | - | - | - | - | 6 | - | - | - | - |
| rs6690386 | 1 | 46253011 | 0.67 | intergenic | IPP(dist=36526),MAST2(dist=16274) | - | - | - | - | - | - | 1f | - | - | - | - |
| rs6690775 | 1 | 46253365 | 0.66 | intergenic | IPP(dist=36880),MAST2(dist=15920) | - | - | - | - | - | - | 6 | - | - | - | - |
| rs550791944 | 1 | 46254920 | 0.62 | intergenic | IPP(dist=38435),MAST2(dist=14365) | - | - | - | - | - | - | - | - | - | - | - |
| rs4630155 | 1 | 46257110 | 0.67 | intergenic | IPP(dist=40625),MAST2(dist=12175) | - | - | - | - | - | - | 6 | - | - | - | - |
| rs4508055 | 1 | 46257731 | 0.67 | intergenic | IPP(dist=41246),MAST2(dist=11554) | - | - | - | - | - | - | 5 | - | - | - | - |
| rs12076580 | 1 | 46258159 | 0.67 | intergenic | IPP(dist=41674),MAST2(dist=11126) | - | - | - | - | - | - | 6 | - | - | - | - |
| rs7551025 | 1 | 46261933 | 0.69 | intergenic | IPP(dist=45448),MAST2(dist=7352) | - | - | - | - | - | - | 6 | - | - | - | - |
| rs7539800 | 1 | 46262129 | 0.69 | intergenic | IPP(dist=45644),MAST2(dist=7156) | - | - | - | - | - | - | 1f | - | - | - | - |
| rs10890350 | 1 | 46262378 | 0.69 | intergenic | IPP(dist=45893),MAST2(dist=6907) | - | - | - | - | - | - | 1f | - | - | - | - |
| rs12077916 | 1 | 46262551 | 0.69 | intergenic | IPP(dist=46066),MAST2(dist=6734) | - | - | - | - | - | - | 7 | - | - | - | - |
| rs11211190 | 1 | 46266255 | 0.69 | intergenic | IPP(dist=49770),MAST2(dist=3030) | - | - | - | - | - | - | 7 | - | - | - | - |
| rs4390216 | 1 | 46266456 | 0.69 | intergenic | IPP(dist=49971),MAST2(dist=2829) | - | - | - | - | - | - | 1f | - | - | - | - |
| rs11369189 | 1 | 46271120 | 0.69 | intronic | MAST2 | Y | Y | Y | - | - | - | 5 | - | - | - | - |
| rs6672115 | 1 | 46272879 | 0.69 | intronic | MAST2 | - | - | - | - | - | - | 6 | - | - | - | - |
| rs10437063 | 1 | 46273666 | 0.69 | intronic | MAST2 | - | - | - | - | - | - | 7 | - | - | - | - |
| rs6676982 | 1 | 46275401 | 0.69 | intronic | MAST2 | - | Y | - | - | - | - | 5 | - | - | - | - |
| rs6429580 | 1 | 46275648 | 0.69 | intronic | MAST2 | - | Y | - | - | - | - | 1d | - | - | - | - |
| rs34566447 | 1 | 46279168 | 0.69 | intronic | MAST2 | - | - | - | - | - | - | 6 | - | - | - | - |
| rs6701694 | 1 | 46279391 | 0.69 | intronic | MAST2 | - | - | - | - | - | - | 7 | - | - | - | - |
| rs11211194 | 1 | 46282084 | 0.69 | intronic | MAST2 | - | - | - | - | - | - | 6 | - | - | - | - |
| rs7546237 | 1 | 46285610 | 0.69 | intronic | MAST2 | - | - | - | - | - | - | 1f | - | - | - | - |
| rs4994390 | 1 | 46287371 | 0.69 | intronic | MAST2 | - | - | - | - | - | - | 6 | - | - | - | - |
| rs11211200 | 1 | 46288352 | 0.69 | intronic | MAST2 | - | Y | - | - | - | - | 1b | - | - | - | - |
| rs12094860 | 1 | 46289055 | 0.69 | intronic | MAST2 | - | - | - | - | - | - | 6 | - | - | - | - |
| rs12094901 | 1 | 46289175 | 0.69 | intronic | MAST2 | - | - | - | - | - | - | 7 | - | - | - | - |
| rs10561161 | 1 | 46290311 | 0.69 | intronic | MAST2 | - | - | - | - | - | - | 5 | - | - | - | - |
| rs6697830 | 1 | 46290477 | 0.69 | intronic | MAST2 | - | - | - | - | - | - | 2c | - | - | - | - |
| rs12060274 | 1 | 46291062 | 0.69 | intronic | MAST2 | - | - | - | - | - | - | 6 | - | - | - | - |
| rs12097761 | 1 | 46291162 | 0.69 | intronic | MAST2 | - | - | - | - | - | - | 7 | - | - | - | - |
| rs11211202 | 1 | 46292382 | 0.69 | intronic | MAST2 | - | - | - | - | - | - | 7 | - | - | - | - |
| rs6674794 | 1 | 46296067 | 0.69 | intronic | MAST2 | - | Y | - | - | - | - | 6 | - | - | - | - |
| rs4660889 | 1 | 46297331 | 0.69 | intronic | MAST2 | - | Y | - | - | - | - | 6 | - | - | - | - |
| rs10890353 | 1 | 46298383 | 0.69 | intronic | MAST2 | Y | Y | - | - | - | - | 7 | - | - | - | - |
| rs4298677 | 1 | 46306619 | 0.69 | intronic | MAST2 | - | Y | - | - | - | - | 1d | - | - | - | - |
| rs4606257 | 1 | 46307268 | 0.69 | intronic | MAST2 | - | Y | - | - | - | - | 7 | - | - | - | - |
| rs10749858 | 1 | 46310005 | 0.69 | intronic | MAST2 | - | Y | - | - | - | - | 5 | - | - | - | - |
| rs10890355 | 1 | 46317348 | 0.69 | intronic | MAST2 | - | - | - | - | - | - | 6 | - | - | - | - |
| rs11211204 | 1 | 46317864 | 0.69 | intronic | MAST2 | - | - | - | - | - | - | 1f | - | - | - | - |
| rs34163992 | 1 | 46318991 | 0.69 | intronic | MAST2 | - | - | - | - | - | - | 7 | - | - | - | - |
| rs10890358 | 1 | 46319025 | 0.69 | intronic | MAST2 | - | - | - | - | - | - | 6 | - | - | - | - |
| rs6429581 | 1 | 46319096 | 0.69 | intronic | MAST2 | - | - | - | - | - | - | 6 | - | - | - | - |
| rs7547189 | 1 | 46319514 | 0.69 | intronic | MAST2 | - | - | - | - | - | - | 6 | - | - | - | - |
| rs7547284 | 1 | 46319565 | 0.69 | intronic | MAST2 | - | - | - | - | - | - | 6 | - | - | - | - |
| rs11211205 | 1 | 46321874 | 0.69 | intronic | MAST2 | - | - | - | - | - | - | 7 | - | - | - | - |
| rs12751443 | 1 | 46324191 | 0.68 | intronic | MAST2 | - | Y | - | - | - | - | 7 | - | - | - | - |
| rs12136641 | 1 | 46324314 | 0.67 | intronic | MAST2 | - | Y | - | - | - | - | 6 | - | - | - | - |
| rs12145287 | 1 | 46325002 | 0.69 | intronic | MAST2 | - | Y | - | - | - | - | 7 | - | - | - | - |
| rs34908920 | 1 | 46325990 | 0.69 | intronic | MAST2 | - | Y | - | - | - | - | 7 | - | - | - | - |
| rs6675222 | 1 | 46326576 | 0.69 | intronic | MAST2 | - | Y | - | - | - | - | 1f | - | - | - | - |
| rs34215190 | 1 | 46327412 | 0.69 | intronic | MAST2 | - | Y | - | - | - | - | 7 | - | - | - | - |
| rs4660895 | 1 | 46329739 | 0.69 | intronic | MAST2 | - | Y | Y | - | - | - | 1f | - | - | - | - |
| rs7531911 | 1 | 46330423 | 0.69 | intronic | MAST2 | - | Y | Y | - | - | - | 1f | - | - | - | - |
| rs34694458 | 1 | 46332696 | 0.69 | intronic | MAST2 | Y | Y | - | - | - | - | 7 | - | - | - | - |
| rs10158032 | 1 | 46332701 | 0.69 | intronic | MAST2 | Y | Y | - | - | - | - | 7 | - | - | - | - |
| rs4660318 | 1 | 46334876 | 0.69 | intronic | MAST2 | Y | Y | Y | - | - | - | 1d | - | - | - | - |
| rs11211209 | 1 | 46335652 | 0.69 | intronic | MAST2 | Y | Y | Y | - | - | - | 6 | - | - | - | - |
| rs72001420 | 1 | 46336186 | 0.71 | intronic | MAST2 | Y | Y | Y | - | - | - | 6 | - | - | - | - |
| rs10890359 | 1 | 46336635 | 0.69 | intronic | MAST2 | Y | Y | Y | - | - | - | 6 | - | - | - | - |
| rs10890360 | 1 | 46338444 | 0.69 | intronic | MAST2 | Y | Y | Y | - | - | - | 7 | - | - | - | - |
| rs6682683 | 1 | 46342337 | 0.69 | intronic | MAST2 | - | Y | - | - | Y | - | 1d | - | - | - | - |
| rs11211215 | 1 | 46347813 | 0.70 | intronic | MAST2 | Y | Y | - | - | - | - | 5 | - | - | - | - |
| rs11211217 | 1 | 46348229 | 0.70 | intronic | MAST2 | Y | Y | - | - | - | - | 1f | - | - | - | - |
| rs10789478 | 1 | 46350506 | 0.69 | intronic | MAST2 | Y | Y | Y | Y | Y | - | 1f | - | - | - | - |
| rs10789479 | 1 | 46352500 | 0.69 | intronic | MAST2 | Y | Y | - | - | - | - | 6 | - | - | - | - |
| rs7553924 | 1 | 46352624 | 0.70 | intronic | MAST2 | Y | Y | - | - | - | - | 7 | - | - | - | - |
| rs4641257 | 1 | 46353589 | 0.70 | intronic | MAST2 | Y | Y | - | - | - | - | 7 | - | - | - | - |
| rs6693336 | 1 | 46354595 | 0.70 | intronic | MAST2 | Y | Y | - | - | - | - | 6 | - | - | - | - |
| rs11211218 | 1 | 46355508 | 0.70 | intronic | MAST2 | Y | Y | - | - | - | - | 5 | - | - | - | - |
| rs4539075 | 1 | 46358010 | 0.70 | intronic | MAST2 | Y | Y | Y | Y | - | - | 1d | - | - | - | - |
| rs34444543 | 1 | 46358862 | 0.69 | intronic | MAST2 | Y | Y | Y | - | - | - | 6 | - | - | - | - |
| rs10890365 | 1 | 46359348 | 0.70 | intronic | MAST2 | Y | Y | Y | - | - | - | 6 | - | - | - | - |
| rs11211219 | 1 | 46359478 | 0.70 | intronic | MAST2 | Y | Y | Y | - | - | - | 6 | - | - | - | - |
| rs7512395 | 1 | 46361177 | 0.69 | intronic | MAST2 | Y | Y | - | - | - | - | 1f | - | - | - | - |
| rs11211222 | 1 | 46367757 | 0.70 | intronic | MAST2 | - | - | - | - | - | - | 4 | - | - | - | - |
| rs7527244 | 1 | 46368772 | 0.70 | intronic | MAST2 | - | - | - | - | - | - | 6 | - | - | - | - |
| rs4524994 | 1 | 46370263 | 0.72 | intronic | MAST2 | - | - | - | - | - | - | 6 | - | - | - | - |
| rs28636523 | 1 | 46371050 | 0.72 | intronic | MAST2 | - | - | - | - | - | - | 7 | - | - | - | - |
| rs9787412 | 1 | 46371596 | 0.72 | intronic | MAST2 | - | - | - | - | - | - | 6 | - | - | - | - |
| rs4660896 | 1 | 46372416 | 0.72 | intronic | MAST2 | - | - | - | - | - | - | 6 | - | - | - | - |
| rs10732844 | 1 | 46372688 | 0.72 | intronic | MAST2 | - | - | - | - | - | - | 1f | - | - | - | - |
| rs12133129 | 1 | 46373348 | 0.72 | intronic | MAST2 | - | - | - | - | - | - | 6 | - | - | - | - |
| rs4553121 | 1 | 46375253 | 0.72 | intronic | MAST2 | - | - | - | - | - | - | 6 | - | - | - | - |
| rs10789480 | 1 | 46375799 | 0.62 | intronic | MAST2 | - | - | - | - | - | - | 5 | - | - | - | - |
| rs6690652 | 1 | 46376692 | 0.72 | intronic | MAST2 | - | - | - | - | - | - | 6 | - | - | - | - |
| rs6695421 | 1 | 46380207 | 0.72 | intronic | MAST2 | - | - | - | - | - | - | 7 | - | - | - | - |
| rs11211223 | 1 | 46380605 | 0.72 | intronic | MAST2 | - | - | - | - | - | - | 1f | - | - | - | - |
| rs12124847 | 1 | 46380850 | 0.72 | intronic | MAST2 | - | - | - | - | - | - | 7 | - | - | - | - |
| rs10890368 | 1 | 46381232 | 0.72 | intronic | MAST2 | - | - | - | - | - | - | 6 | - | - | - | - |
| rs6687301 | 1 | 46382852 | 0.72 | intronic | MAST2 | - | - | - | - | - | - | 6 | - | - | - | - |
| rs12077974 | 1 | 46384141 | 0.70 | intronic | MAST2 | - | - | - | - | - | - | 6 | - | - | - | - |
| rs6657720 | 1 | 46386269 | 0.72 | intronic | MAST2 | - | - | - | - | - | - | 6 | - | - | - | - |
| rs7519181 | 1 | 46387829 | 0.72 | intronic | MAST2 | - | - | - | - | - | - | 5 | - | - | - | - |
| rs10890370 | 1 | 46389217 | 0.72 | intronic | MAST2 | - | - | - | - | - | - | 1f | - | - | - | - |
| rs7526369 | 1 | 46390560 | 0.72 | intronic | MAST2 | - | Y | - | - | - | - | 1d | - | - | - | - |
| rs10890371 | 1 | 46390897 | 0.72 | intronic | MAST2 | - | Y | - | - | - | - | 1f | - | - | - | - |
| rs10890372 | 1 | 46391020 | 0.72 | intronic | MAST2 | - | Y | - | - | - | - | 1f | - | - | - | - |
| rs4074225 | 1 | 46392364 | 0.72 | intronic | MAST2 | - | Y | - | - | - | - | 6 | - | - | - | - |
| rs4297233 | 1 | 46393217 | 0.72 | intronic | MAST2 | - | Y | - | - | - | - | 7 | - | - | - | - |
| rs10890373 | 1 | 46393755 | 0.72 | intronic | MAST2 | - | Y | - | - | - | - | 1f | - | - | - | - |
| rs4134387 | 1 | 46395158 | 0.72 | intronic | MAST2 | Y | Y | Y | Y | Y | - | 1b | - | - | - | - |
| rs6681068 | 1 | 46398382 | 0.72 | intronic | MAST2 | - | - | - | - | - | - | 6 | - | - | - | - |
| rs11211228 | 1 | 46399840 | 0.72 | intronic | MAST2 | - | - | - | - | - | - | 6 | - | - | - | - |
| rs11211229 | 1 | 46400517 | 0.72 | intronic | MAST2 | - | - | - | - | - | - | 7 | - | - | - | - |
| rs9803784 | 1 | 46401907 | 0.72 | intronic | MAST2 | - | - | - | - | - | - | 6 | - | - | - | - |
| rs12022335 | 1 | 46402732 | 0.72 | intronic | MAST2 | - | - | - | - | - | - | 6 | - | - | - | - |
| rs10127690 | 1 | 46403457 | 0.72 | intronic | MAST2 | - | - | - | - | - | - | 6 | - | - | - | - |
| rs34907901 | 1 | 46405214 | 0.63 | intronic | MAST2 | - | - | - | - | - | - | 6 | - | - | - | - |
| rs28831083 | 1 | 46405466 | 0.72 | intronic | MAST2 | - | - | - | - | - | - | 6 | - | - | - | - |
| rs10890374 | 1 | 46406417 | 0.72 | intronic | MAST2 | - | - | - | - | - | - | 6 | - | - | - | - |
| rs10890375 | 1 | 46406581 | 0.71 | intronic | MAST2 | - | - | - | - | - | - | 7 | - | - | - | - |
| rs10890376 | 1 | 46406582 | 0.71 | intronic | MAST2 | - | - | - | - | - | - | 7 | - | - | - | - |
| rs10890377 | 1 | 46406594 | 0.71 | intronic | MAST2 | - | - | - | - | - | - | 6 | - | - | - | - |
| rs10789484 | 1 | 46406978 | 0.72 | intronic | MAST2 | - | - | - | - | - | - | 7 | - | - | - | - |
| rs10789485 | 1 | 46407308 | 0.72 | intronic | MAST2 | - | - | - | - | - | - | 6 | - | - | - | - |
| rs10789486 | 1 | 46408667 | 0.72 | intronic | MAST2 | - | Y | - | - | - | - | 6 | - | - | - | - |
| rs10890378 | 1 | 46410657 | 0.72 | intronic | MAST2 | - | Y | - | - | - | - | 6 | - | - | - | - |
| rs11211232 | 1 | 46411146 | 0.72 | intronic | MAST2 | - | Y | - | - | - | - | 1f | - | - | - | - |
| rs7527079 | 1 | 46413450 | 0.72 | intronic | MAST2 | - | Y | - | - | - | - | 7 | - | - | - | - |
| rs7527298 | 1 | 46413698 | 0.72 | intronic | MAST2 | - | Y | - | - | - | - | 7 | - | - | - | - |
| rs61785584 | 1 | 46414107 | 0.72 | intronic | MAST2 | - | Y | - | - | - | - | 7 | - | - | - | - |
| rs34446427 | 1 | 46417655 | 0.72 | intronic | MAST2 | Y | Y | - | - | - | - | 6 | - | - | - | - |
| rs4660900 | 1 | 46419605 | 0.72 | intronic | MAST2 | Y | Y | Y | - | - | - | 1f | - | - | - | - |
| rs4454479 | 1 | 46423555 | 0.72 | intronic | MAST2 | - | Y | - | - | - | - | 3a | - | - | - | - |
| rs7517560 | 1 | 46425943 | 0.71 | intronic | MAST2 | - | Y | - | - | - | - | 7 | - | - | - | - |
| rs143955886 | 1 | 46427838 | 0.71 | intronic | MAST2 | - | Y | - | - | - | - | 7 | - | - | - | - |
| rs7516008 | 1 | 46429427 | 0.72 | intronic | MAST2 | - | Y | - | - | - | - | 5 | - | - | - | - |
| rs7530103 | 1 | 46429431 | 0.72 | intronic | MAST2 | - | Y | - | - | - | - | 6 | - | - | - | - |
| rs12403666 | 1 | 46431621 | 0.72 | intronic | MAST2 | - | Y | - | - | - | - | 5 | - | - | - | - |
| rs12077546 | 1 | 46431806 | 0.72 | intronic | MAST2 | - | Y | - | - | - | - | 7 | - | - | - | - |
| rs12124291 | 1 | 46432132 | 0.72 | intronic | MAST2 | - | Y | - | - | - | - | 6 | - | - | - | - |
| rs6671754 | 1 | 46433990 | 0.72 | intronic | MAST2 | - | Y | - | - | - | - | 6 | - | - | - | - |
| rs12067716 | 1 | 46434428 | 0.72 | intronic | MAST2 | - | Y | - | - | - | - | 5 | - | - | - | - |
| rs6666763 | 1 | 46436451 | 0.72 | intronic | MAST2 | - | Y | - | - | - | - | 6 | - | - | - | - |
| rs4489497 | 1 | 46438698 | 0.72 | intronic | MAST2 | - | Y | - | - | - | - | 5 | - | - | - | - |
| rs35482380 | 1 | 46438922 | 0.72 | intronic | MAST2 | - | Y | Y | - | - | - | 3a | - | - | - | - |
| rs11211235 | 1 | 46440375 | 0.72 | intronic | MAST2 | - | Y | - | - | - | - | 6 | - | - | - | - |
| rs9919275 | 1 | 46441360 | 0.72 | intronic | MAST2 | - | Y | - | - | - | - | 2b | - | - | - | - |
| rs11211236 | 1 | 46443767 | 0.72 | intronic | MAST2 | - | Y | - | - | - | - | 1f | - | - | - | - |
| rs4660328 | 1 | 46444831 | 0.72 | intronic | MAST2 | Y | Y | Y | - | Y | - | 1b | - | - | - | - |
| rs61785613 | 1 | 46445871 | 0.72 | intronic | MAST2 | - | Y | - | - | - | - | 7 | - | - | - | - |
| rs61785614 | 1 | 46446047 | 0.72 | intronic | MAST2 | - | Y | - | - | - | - | 6 | - | - | - | - |
| rs9793167 | 1 | 46446800 | 0.72 | intronic | MAST2 | - | Y | - | - | - | - | 5 | - | - | - | - |
| rs11211237 | 1 | 46447329 | 0.72 | intronic | MAST2 | - | Y | - | - | - | - | 1f | - | - | - | - |
| rs11211238 | 1 | 46449415 | 0.72 | intronic | MAST2 | - | Y | - | - | - | - | 7 | - | - | - | - |
| rs10890380 | 1 | 46449469 | 0.72 | intronic | MAST2 | - | Y | - | - | - | - | 6 | - | - | - | - |
| rs4460583 | 1 | 46449918 | 0.72 | intronic | MAST2 | - | Y | - | - | - | - | 5 | - | - | - | - |
| rs4459051 | 1 | 46451203 | 0.72 | intronic | MAST2 | - | Y | - | - | - | - | 7 | - | - | - | - |
| rs10890381 | 1 | 46451771 | 0.72 | intronic | MAST2 | - | Y | - | - | - | - | 6 | - | - | - | - |
| rs11211239 | 1 | 46451947 | 0.72 | intronic | MAST2 | - | Y | - | - | - | - | 6 | - | - | - | - |
| rs6429588 | 1 | 46454058 | 0.72 | intronic | MAST2 | - | Y | - | - | - | - | 6 | - | - | - | - |
| rs34619960 | 1 | 46454535 | 0.72 | intronic | MAST2 | - | Y | - | - | - | - | 7 | - | - | - | - |
| rs11211243 | 1 | 46455433 | 0.72 | intronic | MAST2 | - | - | - | - | - | - | 6 | - | - | - | - |
| rs11211244 | 1 | 46456673 | 0.72 | intronic | MAST2 | - | - | - | - | - | - | 6 | - | - | - | - |
| rs4660903 | 1 | 46457601 | 0.72 | intronic | MAST2 | - | - | - | - | - | - | 6 | - | - | - | - |
| rs4660331 | 1 | 46458783 | 0.72 | intronic | MAST2 | - | - | - | - | - | - | 7 | - | - | - | - |
| rs12097799 | 1 | 46459886 | 0.72 | intronic | MAST2 | - | - | - | - | - | - | 2c | - | - | - | - |
| rs4660904 | 1 | 46460667 | 0.72 | intronic | MAST2 | - | - | - | - | - | - | 7 | - | - | - | - |
| rs7534331 | 1 | 46460766 | 0.72 | intronic | MAST2 | - | - | - | - | - | - | 6 | - | - | - | - |
| rs4660332 | 1 | 46461418 | 0.72 | intronic | MAST2 | - | - | - | - | - | - | 5 | - | - | - | - |
| rs4660905 | 1 | 46461587 | 0.72 | intronic | MAST2 | - | - | - | - | - | - | 1f | - | - | - | - |
| rs4660334 | 1 | 46462126 | 0.72 | intronic | MAST2 | - | - | - | - | - | - | 1f | - | - | - | - |
| rs4073847 | 1 | 46463012 | 0.72 | intronic | MAST2 | - | - | - | - | - | - | 6 | - | - | - | - |
| rs6661500 | 1 | 46465662 | 0.72 | intronic | MAST2 | - | - | - | - | - | - | 6 | - | - | - | - |
| rs10890382 | 1 | 46469674 | 0.72 | intronic | MAST2 | - | - | - | - | - | - | 6 | - | - | - | - |
| rs4660906 | 1 | 46470860 | 0.72 | intronic | MAST2 | - | - | - | - | - | - | 6 | - | - | - | - |
| rs3922887 | 1 | 46473802 | 0.72 | intronic | MAST2 | - | - | - | - | - | - | 6 | - | - | - | - |
| rs3922886 | 1 | 46473902 | 0.72 | intronic | MAST2 | - | - | - | - | - | - | 7 | - | - | - | - |
| rs4660335 | 1 | 46474718 | 0.72 | intronic | MAST2 | - | - | - | - | - | - | 6 | - | - | - | - |
| rs6677777 | 1 | 46475836 | 0.72 | intronic | MAST2 | - | - | - | - | - | - | 1f | - | - | - | - |
| rs35155395 | 1 | 46476746 | 0.72 | intronic | MAST2 | - | - | - | - | - | - | 5 | - | - | - | - |
| rs11211248 | 1 | 46476844 | 0.72 | intronic | MAST2 | - | - | - | - | - | - | 1f | - | - | - | - |
| rs201377643 | 1 | 46481799 | 0.71 | intronic | MAST2 | - | - | - | - | - | - | 7 | - | - | - | - |
| rs74507939 | 1 | 46481939 | 0.72 | intronic | MAST2 | - | - | - | - | - | - | 7 | - | - | - | - |
| rs9429175 | 1 | 46482691 | 0.72 | intronic | MAST2 | - | - | - | - | - | - | 7 | - | - | - | - |
| rs9429176 | 1 | 46482835 | 0.72 | intronic | MAST2 | - | - | - | - | - | - | 6 | - | - | - | - |
| rs7532204 | 1 | 46485075 | 0.72 | intronic | MAST2 | - | - | - | - | - | - | 7 | - | - | - | - |
| rs946528 | 1 | 46485562 | 0.69 | intronic | MAST2 | - | - | - | - | - | - | 6 | - | - | - | - |
| rs946527 | 1 | 46485970 | 0.71 | intronic | MAST2 | - | - | - | - | - | - | 1f | - | - | - | - |
| rs946525 | 1 | 46487277 | 0.72 | intronic | MAST2 | - | - | - | - | - | - | 2b | - | - | - | - |
| rs946524 | 1 | 46487560 | 0.72 | intronic | MAST2 | - | - | - | - | - | - | 6 | - | - | - | - |
| rs4660336 | 1 | 46487976 | 0.72 | intronic | MAST2 | - | - | - | - | - | - | 5 | - | - | - | - |
| rs785477 | 1 | 46490654 | 0.71 | intronic | MAST2 | - | - | - | - | - | - | 7 | - | - | - | - |
| rs785478 | 1 | 46491635 | 0.72 | intronic | MAST2 | - | - | - | - | - | - | 1f | - | - | - | - |
| rs785480 | 1 | 46492164 | 0.72 | intronic | MAST2 | - | - | - | - | - | - | 1f | - | - | - | - |
| rs925524 | 1 | 46496709 | 0.73 | exonic | MAST2 | - | - | - | - | - | - | 1f | - | - | - | - |
| rs1622208 | 1 | 46498375 | 0.73 | exonic | MAST2 | - | - | - | - | - | - | 1f | - | - | - | - |
| rs371512102 | 1 | 46498648 | 0.73 | intronic | MAST2 | - | - | - | - | - | - | 6 | - | - | - | - |
| rs2669156 | 1 | 46498831 | 0.73 | intronic | MAST2 | - | - | - | - | - | - | 6 | - | - | - | - |
| rs6675259 | 1 | 46502295 | 0.72 | downstream | MAST2 | - | Y | - | - | - | - | 1f | - | - | - | - |
| rs7538978 | 1 | 46505054 | 0.76 | downstream | PIK3R3 | - | - | - | - | - | - | 1f | - | - | - | - |
| rs1707322 | 1 | 46505147 | 0.77 | downstream | PIK3R3 | - | - | - | - | - | - | 1f | - | - | - | - |
| rs11444102 | 1 | 46505589 | 0.76 | downstream | PIK3R3 | - | - | - | - | - | - | 4 | - | - | - | - |
| rs71062735 | 1 | 46505785 | 0.76 | downstream | PIK3R3 | - | - | - | - | - | - | 3a | - | - | - | - |
| rs1707337 | 1 | 46508769 | 0.77 | UTR3 | PIK3R3(NM_001114172:c.*5760>0,NM_003629:c.*5760>0,NM_001303428:c.*5760>0,NM_001303427:c.*5760>0,NM_001303429:c.*5760>0) | - | - | - | - | - | - | 6 | - | - | - | - |
| rs1768807 | 1 | 46510570 | 0.77 | intronic | PIK3R3 | - | - | - | - | - | - | 1f | - | - | - | - |
| rs1707317 | 1 | 46510642 | 0.77 | intronic | PIK3R3 | - | - | - | - | - | - | 1f | - | - | - | - |
| rs2297883 | 1 | 46511487 | 0.77 | intronic | PIK3R3 | - | - | - | - | - | - | 7 | - | - | - | - |
| rs1707338 | 1 | 46511981 | 0.77 | intronic | PIK3R3 | - | - | - | - | - | - | 6 | - | - | - | - |
| rs1707339 | 1 | 46513179 | 0.77 | intronic | PIK3R3 | - | - | - | - | - | - | 6 | - | - | - | - |
| rs1768818 | 1 | 46514286 | 0.77 | intronic | PIK3R3 | - | - | - | - | - | - | 6 | - | - | - | - |
| rs1768817 | 1 | 46514534 | 0.77 | intronic | PIK3R3 | - | - | - | - | - | - | 6 | - | - | - | - |
| rs785470 | 1 | 46519622 | 0.77 | intronic | PIK3R3 | - | - | - | - | - | - | 1a | - | - | - | - |
| rs5773899 | 1 | 46520826 | 0.77 | intronic | PIK3R3 | - | - | - | - | - | - | 7 | - | - | - | - |
| rs785469 | 1 | 46521091 | 0.77 | intronic | PIK3R3 | - | - | - | - | - | - | 6 | - | - | - | - |
| rs785468 | 1 | 46521517 | 0.77 | exonic | PIK3R3 | - | - | - | - | - | synonymous | 6 | 0.051 | T | 0.986 | D |
| rs785467 | 1 | 46521559 | 0.77 | exonic | PIK3R3 | - | - | - | - | - | missense | 6 | 1.000 | T | 0.000 | B |
| rs785466 | 1 | 46521792 | 0.77 | intronic | PIK3R3 | - | - | - | - | - | - | 7 | - | - | - | - |
| rs785465 | 1 | 46522577 | 0.77 | intronic | PIK3R3 | - | - | - | - | - | - | 6 | - | - | - | - |
| rs1085240 | 1 | 46523978 | 0.77 | intronic | PIK3R3 | - | - | - | - | - | - | 7 | - | - | - | - |
| rs785463 | 1 | 46524234 | 0.77 | intronic | PIK3R3 | - | - | - | - | - | - | 6 | - | - | - | - |
| rs785462 | 1 | 46525751 | 0.77 | intronic | PIK3R3 | - | - | - | - | - | - | 6 | - | - | - | - |
| rs785508 | 1 | 46527159 | 0.69 | intronic | PIK3R3 | - | - | - | - | - | - | 6 | - | - | - | - |
| rs785509 | 1 | 46527300 | 0.69 | intronic | PIK3R3 | - | - | - | - | - | - | 1f | - | - | - | - |
| rs112919607 | 1 | 46528248 | 0.76 | intronic | PIK3R3 | - | - | Y | - | - | - | 5 | - | - | - | - |
| rs1768815 | 1 | 46528603 | 0.76 | intronic | PIK3R3 | - | Y | Y | - | - | - | 3a | - | - | - | - |
| rs785510 | 1 | 46528618 | 0.76 | intronic | PIK3R3 | - | Y | Y | - | - | - | 1f | - | - | - | - |
| rs2458400 | 1 | 46530798 | 0.77 | intronic | PIK3R3 | - | - | - | - | - | - | 6 | - | - | - | - |
| rs785513 | 1 | 46538320 | 0.69 | intronic | PIK3R3 | - | - | - | - | - | - | 6 | - | - | - | - |
| rs35378730 | 1 | 46539458 | 0.75 | intronic | PIK3R3 | - | - | - | - | - | - | 6 | - | - | - | - |
| rs9429186 | 1 | 46539687 | 0.77 | intronic | PIK3R3 | - | - | - | - | - | - | 7 | - | - | - | - |
| rs785506 | 1 | 46540652 | 0.77 | intronic | PIK3R3 | - | - | - | - | - | - | 1f | - | - | - | - |
| rs785507 | 1 | 46541558 | 0.77 | intronic | PIK3R3 | - | - | - | - | - | - | 6 | - | - | - | - |
| rs34234237 | 1 | 46544776 | 0.77 | intronic | PIK3R3 | - | - | - | - | - | - | 6 | - | - | - | - |
| rs1613296 | 1 | 46546852 | 0.77 | intronic | PIK3R3 | - | - | - | - | - | - | 6 | - | - | - | - |
| rs1612419 | 1 | 46546945 | 0.77 | intronic | PIK3R3 | - | - | - | - | - | - | 6 | - | - | - | - |
| rs1768802 | 1 | 46547868 | 0.77 | intronic | PIK3R3 | - | - | - | - | - | - | 7 | - | - | - | - |
| rs1588663 | 1 | 46548825 | 0.77 | intronic | PIK3R3 | - | - | - | - | - | - | 6 | - | - | - | - |
| rs1768801 | 1 | 46549137 | 0.77 | intronic | PIK3R3 | - | - | - | - | - | - | 6 | - | - | - | - |
| rs1768800 | 1 | 46549188 | 0.77 | intronic | PIK3R3 | - | - | - | - | - | - | 6 | - | - | - | - |
| rs143524711 | 1 | 46555984 | 0.69 | intronic | PIK3R3 | - | - | - | - | - | - | 6 | - | - | - | - |
| rs35808728 | 1 | 46558492 | 0.76 | intronic | PIK3R3 | - | - | - | - | - | - | 6 | - | - | - | - |
| rs785483 | 1 | 46559171 | 0.77 | intronic | PIK3R3 | - | - | - | - | - | - | 6 | - | - | - | - |
| rs809774 | 1 | 46560244 | 0.69 | intronic | PIK3R3 | - | - | - | - | - | - | 1f | - | - | - | - |
| rs796773 | 1 | 46564475 | 0.77 | intronic | PIK3R3 | - | - | - | - | - | - | 5 | - | - | - | - |
| rs59197560 | 1 | 46564539 | 0.69 | intronic | PIK3R3 | - | - | - | - | - | - | 6 | - | - | - | - |
| rs785516 | 1 | 46564758 | 0.77 | intronic | PIK3R3 | - | - | - | - | - | - | 5 | - | - | - | - |
| rs785517 | 1 | 46567535 | 0.77 | intronic | PIK3R3 | - | - | - | - | - | - | 6 | - | - | - | - |
| rs785518 | 1 | 46568422 | 0.77 | intronic | PIK3R3 | - | - | - | - | - | - | 6 | - | - | - | - |
| rs785519 | 1 | 46568562 | 0.77 | intronic | PIK3R3 | - | - | - | - | - | - | 7 | - | - | - | - |
| rs785484 | 1 | 46574015 | 0.77 | intronic | PIK3R3 | - | - | - | - | - | - | 6 | - | - | - | - |
| rs785486 | 1 | 46575148 | 0.77 | intronic | PIK3R3 | - | - | - | - | - | - | 6 | - | - | - | - |
| rs785490 | 1 | 46577124 | 0.77 | intronic | PIK3R3 | - | - | - | - | - | - | 6 | - | - | - | - |
| rs785501 | 1 | 46579611 | 0.77 | intronic | PIK3R3 | - | - | - | - | - | - | 5 | - | - | - | - |
| rs67716739 | 1 | 46583995 | 0.77 | intronic | PIK3R3 | - | - | - | - | - | - | 3a | - | - | - | - |
| rs785493 | 1 | 46584859 | 0.77 | intronic | PIK3R3 | - | - | - | - | - | - | 6 | - | - | - | - |
| rs785496 | 1 | 46588006 | 0.77 | intronic | PIK3R3 | - | - | - | - | - | - | 6 | - | - | - | - |
| rs386366888 | 1 | 46590468 | 0.77 | intronic | PIK3R3 | - | - | - | - | - | - | 6 | - | - | - | - |
| rs814168 | 1 | 46591623 | 0.77 | intronic | PIK3R3 | - | - | - | - | - | - | 6 | - | - | - | - |
| rs785497 | 1 | 46591903 | 0.77 | intronic | PIK3R3 | - | - | - | - | - | - | 6 | - | - | - | - |
| rs785498 | 1 | 46592414 | 0.69 | intronic | PIK3R3 | - | - | - | - | - | - | 6 | - | - | - | - |
| rs785499 | 1 | 46592420 | 0.77 | intronic | PIK3R3 | - | - | - | - | - | - | 6 | - | - | - | - |
| rs785500 | 1 | 46593044 | 0.77 | intronic | PIK3R3 | - | - | - | - | - | - | 6 | - | - | - | - |
| rs28482847 | 1 | 46595077 | 0.70 | intronic | PIK3R3 | - | - | - | - | - | - | 6 | - | - | - | - |
| rs4660342 | 1 | 46595082 | 0.70 | intronic | PIK3R3 | - | - | - | - | - | - | 6 | - | - | - | - |
| rs785504 | 1 | 46595696 | 0.77 | intronic | PIK3R3 | - | Y | Y | - | - | - | 6 | - | - | - | - |
| rs1707303 | 1 | 46598273 | 0.77 | UTR5 | PIK3R3(NM_003629:c.-6490>0,NM_001303429:c.-6490>0) | Y | - | Y | - | Y | - | 4 | - | - | - | - |
| **rs1707302** | 1 | 46600917 | 1.00 | ncRNA_intronic | LOC101929626 | - | Y | - | - | - | - | 1f | - | - | - | - |
| rs1473840 | 1 | 46601197 | 1.00 | ncRNA_intronic | LOC101929626 | - | Y | - | - | - | - | 2b | - | - | - | - |
| rs1416706 | 1 | 46602844 | 0.99 | ncRNA_intronic | LOC101929626 | - | - | - | - | - | - | 1f | - | - | - | - |
| rs9429095 | 1 | 46603348 | 0.99 | ncRNA_intronic | LOC101929626 | - | - | - | - | - | - | 6 | - | - | - | - |
| rs2523558 | 6 | 31331037 | 0.62 | intergenic | HLA-B(dist=6048),MICA(dist=36524) | - | - | - | - | - | - | 6 | - | - | - | - |
| rs2523554 | 6 | 31331829 | 0.62 | intergenic | HLA-B(dist=6840),MICA(dist=35732) | - | - | - | - | - | - | 1f | - | - | - | - |
| rs2853993 | 6 | 31333191 | 0.62 | intergenic | HLA-B(dist=8202),MICA(dist=34370) | - | - | - | - | - | - | 7 | - | - | - | - |
| rs578039100 | 6 | 31377719 | 0.66 | intronic | MICA | - | - | - | - | - | - | - | - | - | - | - |
| rs2853981 | 6 | 31378768 | 1.00 | intronic | MICA | - | - | - | - | - | - | 6 | - | - | - | - |
| rs2853977 | 6 | 31379304 | 1.00 | intronic | MICA | - | - | - | - | - | - | 5 | - | - | - | - |
| rs2256174 | 6 | 31380422 | 1.00 | intronic | MICA | - | - | - | - | - | - | 5 | - | - | - | - |
| rs2256183 | 6 | 31380529 | 1.00 | intronic | MICA | - | - | - | - | - | - | 6 | - | - | - | - |
| rs1882 | 6 | 31382911 | 0.93 | exonic | MICA | - | - | - | - | - | missense | 5 | - | - | - | - |
| rs2596528 | 6 | 31385485 | 1.00 | intergenic | MICA(dist=2393),HCP5(dist=45472) | - | - | - | - | - | - | 6 | - | - | - | - |
| rs2596530 | 6 | 31387373 | 1.00 | intergenic | MICA(dist=4281),HCP5(dist=43584) | - | - | - | - | - | - | 6 | - | - | - | - |
| rs2596531 | 6 | 31387557 | 1.00 | intergenic | MICA(dist=4465),HCP5(dist=43400) | - | - | - | - | - | - | 4 | - | - | - | - |
| rs559235119 | 6 | 31388175 | 1.00 | intergenic | MICA(dist=5083),HCP5(dist=42782) | - | - | - | - | - | - | - | - | - | - | - |
| rs2844511 | 6 | 31389784 | 1.00 | intergenic | MICA(dist=6692),HCP5(dist=41173) | - | - | - | - | - | - | 6 | - | - | - | - |
| **rs2516448** | 6 | 31390410 | 1.00 | intergenic | MICA(dist=7318),HCP5(dist=40547) | - | - | - | - | - | - | 5 | - | - | - | - |
| rs3998770 | 6 | 29738042 | 0.65 | intergenic | IFITM4P(dist=19117),HCG4(dist=20766) | - | - | - | - | - | - | 5 | - | - | - | - |
| rs3888722 | 6 | 29739000 | 0.66 | intergenic | IFITM4P(dist=20075),HCG4(dist=19808) | - | - | - | - | - | - | 7 | - | - | - | - |
| rs62391850 | 6 | 29740024 | 0.65 | intergenic | IFITM4P(dist=21099),HCG4(dist=18784) | - | - | - | - | - | - | 5 | - | - | - | - |
| rs62391851 | 6 | 29740548 | 0.65 | intergenic | IFITM4P(dist=21623),HCG4(dist=18260) | - | - | - | - | - | - | 5 | - | - | - | - |
| rs56392172 | 6 | 29741535 | 0.65 | intergenic | IFITM4P(dist=22610),HCG4(dist=17273) | - | - | - | - | - | - | 6 | - | - | - | - |
| rs62394668 | 6 | 29742842 | 0.66 | intergenic | IFITM4P(dist=23917),HCG4(dist=15966) | - | - | - | - | - | - | 6 | - | - | - | - |
| rs62394669 | 6 | 29744135 | 0.65 | intergenic | IFITM4P(dist=25210),HCG4(dist=14673) | - | - | - | - | - | - | 6 | - | - | - | - |
| rs199677824 | 6 | 29744210 | 0.65 | intergenic | IFITM4P(dist=25285),HCG4(dist=14598) | - | - | - | - | - | - | 6 | - | - | - | - |
| rs62394670 | 6 | 29745211 | 0.65 | intergenic | IFITM4P(dist=26286),HCG4(dist=13597) | - | - | - | - | - | - | 5 | - | - | - | - |
| rs139910383 | 6 | 29748509 | 0.66 | intergenic | IFITM4P(dist=29584),HCG4(dist=10299) | - | - | - | - | - | - | 7 | - | - | - | - |
| rs56290866 | 6 | 29753583 | 0.65 | intergenic | IFITM4P(dist=34658),HCG4(dist=5225) | - | - | - | - | - | - | 7 | - | - | - | - |
| rs67382831 | 6 | 29753831 | 0.66 | intergenic | IFITM4P(dist=34906),HCG4(dist=4977) | - | - | - | - | - | - | 5 | - | - | - | - |
| rs116487225 | 6 | 29754368 | 0.64 | intergenic | IFITM4P(dist=35443),HCG4(dist=4440) | - | - | - | - | - | - | 6 | - | - | - | - |
| rs2394172 | 6 | 29756800 | 0.63 | intergenic | IFITM4P(dist=37875),HCG4(dist=2008) | - | - | - | - | - | - | 5 | - | - | - | - |
| rs45479291 | 6 | 29760029 | 0.66 | ncRNA_exonic | HCG4,LOC554223 | - | Y | Y | - | - | missense | 4 | - | - | - | - |
| rs45458093 | 6 | 29760536 | 0.66 | ncRNA_exonic | HCG4 | - | - | Y | - | - | - | 4 | - | - | - | - |
| rs16895757 | 6 | 29762178 | 0.66 | ncRNA_intronic | LOC554223 | - | - | - | - | - | - | 5 | - | - | - | - |
| rs55791644 | 6 | 29762639 | 0.65 | ncRNA_intronic | LOC554223 | - | - | - | - | - | - | 5 | - | - | - | - |
| rs578105416 | 6 | 29763515 | 0.65 | ncRNA_intronic | LOC554223 | - | - | - | - | - | - | - | - | - | - | - |
| rs56104249 | 6 | 29766070 | 0.66 | downstream | LOC554223 | - | - | - | - | - | - | 5 | - | - | - | - |
| rs62394676 | 6 | 29766301 | 0.66 | downstream | LOC554223 | - | - | - | - | - | - | 6 | - | - | - | - |
| rs56353080 | 6 | 29781559 | 0.65 | intergenic | LOC554223(dist=15975),HLA-G(dist=13197) | - | - | - | - | - | - | 2b | - | - | - | - |
| rs62394678 | 6 | 29783419 | 0.66 | intergenic | LOC554223(dist=17835),HLA-G(dist=11337) | - | - | - | - | - | - | 3a | - | - | - | - |
| rs3890904 | 6 | 29783491 | 0.64 | intergenic | LOC554223(dist=17907),HLA-G(dist=11265) | - | - | - | - | - | - | 5 | - | - | - | - |
| rs58075773 | 6 | 29784310 | 0.64 | intergenic | LOC554223(dist=18726),HLA-G(dist=10446) | - | - | - | - | - | - | 6 | - | - | - | - |
| rs61609080 | 6 | 29784543 | 0.64 | intergenic | LOC554223(dist=18959),HLA-G(dist=10213) | - | - | - | - | - | - | 7 | - | - | - | - |
| rs115920041 | 6 | 29784769 | 0.64 | intergenic | LOC554223(dist=19185),HLA-G(dist=9987) | - | - | - | - | - | - | 6 | - | - | - | - |
| rs114181339 | 6 | 29784983 | 0.65 | intergenic | LOC554223(dist=19399),HLA-G(dist=9773) | - | - | - | - | - | - | 5 | - | - | - | - |
| rs62394681 | 6 | 29785070 | 0.66 | intergenic | LOC554223(dist=19486),HLA-G(dist=9686) | - | - | - | - | - | - | 5 | - | - | - | - |
| rs17179010 | 6 | 29785662 | 0.66 | intergenic | LOC554223(dist=20078),HLA-G(dist=9094) | - | - | - | - | - | - | 7 | - | - | - | - |
| rs17179017 | 6 | 29785671 | 0.64 | intergenic | LOC554223(dist=20087),HLA-G(dist=9085) | - | - | - | - | - | - | 6 | - | - | - | - |
| rs17185419 | 6 | 29785827 | 0.66 | intergenic | LOC554223(dist=20243),HLA-G(dist=8929) | - | - | - | - | - | - | 7 | - | - | - | - |
| rs17185426 | 6 | 29785828 | 0.66 | intergenic | LOC554223(dist=20244),HLA-G(dist=8928) | - | - | - | - | - | - | 7 | - | - | - | - |
| rs17179038 | 6 | 29785958 | 0.64 | intergenic | LOC554223(dist=20374),HLA-G(dist=8798) | - | - | - | - | - | - | 6 | - | - | - | - |
| rs17185440 | 6 | 29786057 | 0.64 | intergenic | LOC554223(dist=20473),HLA-G(dist=8699) | - | - | - | - | - | - | 6 | - | - | - | - |
| rs17185447 | 6 | 29786214 | 0.64 | intergenic | LOC554223(dist=20630),HLA-G(dist=8542) | - | - | - | - | - | - | 6 | - | - | - | - |
| rs17179066 | 6 | 29786265 | 0.65 | intergenic | LOC554223(dist=20681),HLA-G(dist=8491) | - | - | - | - | - | - | 7 | - | - | - | - |
| rs114532713 | 6 | 29786335 | 0.66 | intergenic | LOC554223(dist=20751),HLA-G(dist=8421) | - | - | - | - | - | - | 6 | - | - | - | - |
| rs113998437 | 6 | 29786346 | 0.64 | intergenic | LOC554223(dist=20762),HLA-G(dist=8410) | - | - | - | - | - | - | 7 | - | - | - | - |
| rs115128443 | 6 | 29786352 | 0.64 | intergenic | LOC554223(dist=20768),HLA-G(dist=8404) | - | - | - | - | - | - | 7 | - | - | - | - |
| rs62394682 | 6 | 29786409 | 0.64 | intergenic | LOC554223(dist=20825),HLA-G(dist=8347) | - | - | - | - | - | - | 6 | - | - | - | - |
| rs56067910 | 6 | 29786766 | 0.63 | intergenic | LOC554223(dist=21182),HLA-G(dist=7990) | - | - | - | - | - | - | 7 | - | - | - | - |
| rs56128518 | 6 | 29786873 | 0.66 | intergenic | LOC554223(dist=21289),HLA-G(dist=7883) | - | - | - | - | - | - | 6 | - | - | - | - |
| rs55729140 | 6 | 29787532 | 0.64 | intergenic | LOC554223(dist=21948),HLA-G(dist=7224) | - | - | - | - | - | - | 7 | - | - | - | - |
| rs55970611 | 6 | 29787649 | 0.64 | intergenic | LOC554223(dist=22065),HLA-G(dist=7107) | - | - | - | - | - | - | 6 | - | - | - | - |
| rs3052969 | 6 | 29788254 | 0.66 | intergenic | LOC554223(dist=22670),HLA-G(dist=6502) | - | - | - | - | - | - | 7 | - | - | - | - |
| rs3873245 | 6 | 29788537 | 0.64 | intergenic | LOC554223(dist=22953),HLA-G(dist=6219) | - | - | - | - | - | - | 7 | - | - | - | - |
| rs56324548 | 6 | 29788794 | 0.64 | intergenic | LOC554223(dist=23210),HLA-G(dist=5962) | - | - | - | - | - | - | 7 | - | - | - | - |
| rs56236016 | 6 | 29790563 | 0.66 | intergenic | LOC554223(dist=24979),HLA-G(dist=4193) | - | - | - | - | - | - | 5 | - | - | - | - |
| rs114588158 | 6 | 29791992 | 0.66 | intergenic | LOC554223(dist=26408),HLA-G(dist=2764) | - | - | - | - | - | - | 5 | - | - | - | - |
| rs62391962 | 6 | 29792442 | 0.66 | intergenic | LOC554223(dist=26858),HLA-G(dist=2314) | - | - | - | - | - | - | 7 | - | - | - | - |
| rs62391963 | 6 | 29792470 | 0.66 | intergenic | LOC554223(dist=26886),HLA-G(dist=2286) | - | - | - | - | - | - | 7 | - | - | - | - |
| rs3873252 | 6 | 29796369 | 0.66 | exonic | HLA-G | - | - | - | - | - | - | 3a | - | - | - | - |
| rs62391965 | 6 | 29796749 | 0.67 | intronic | HLA-G | - | - | - | - | - | - | 5 | - | - | - | - |
| rs114038308 | 6 | 29798083 | 0.66 | intronic | HLA-G | - | - | - | - | - | - | 5 | - | - | - | - |
| rs17179101 | 6 | 29798634 | 0.66 | UTR3 | HLA-G(NM_002127:c.*1180>0) | - | - | - | - | - | - | 6 | - | - | - | - |
| rs3888781 | 6 | 29799383 | 0.66 | downstream | HLA-G | - | - | - | - | - | - | 6 | - | - | - | - |
| rs3869053 | 6 | 29800067 | 0.69 | intergenic | HLA-G(dist=1168),HLA-H(dist=55470) | - | - | - | - | - | - | 5 | - | - | - | - |
| rs62391966 | 6 | 29801113 | 0.68 | intergenic | HLA-G(dist=2214),HLA-H(dist=54424) | - | - | - | - | - | - | 7 | - | - | - | - |
| rs56297686 | 6 | 29801652 | 0.68 | intergenic | HLA-G(dist=2753),HLA-H(dist=53885) | - | - | - | - | - | - | 6 | - | - | - | - |
| rs62391982 | 6 | 29802676 | 0.68 | intergenic | HLA-G(dist=3777),HLA-H(dist=52861) | - | - | - | - | - | - | 4 | - | - | - | - |
| rs62391984 | 6 | 29802893 | 0.68 | intergenic | HLA-G(dist=3994),HLA-H(dist=52644) | - | - | - | Y | Y | - | 4 | - | - | - | - |
| rs17179129 | 6 | 29802971 | 0.68 | intergenic | HLA-G(dist=4072),HLA-H(dist=52566) | - | - | - | - | - | - | 3a | - | - | - | - |
| rs17179136 | 6 | 29802978 | 0.67 | intergenic | HLA-G(dist=4079),HLA-H(dist=52559) | - | - | - | - | - | - | 4 | - | - | - | - |
| rs17185538 | 6 | 29803114 | 0.68 | intergenic | HLA-G(dist=4215),HLA-H(dist=52423) | - | - | - | - | - | - | 4 | - | - | - | - |
| rs17179157 | 6 | 29803341 | 0.68 | intergenic | HLA-G(dist=4442),HLA-H(dist=52196) | - | - | - | - | - | - | 7 | - | - | - | - |
| rs17179171 | 6 | 29803382 | 0.68 | intergenic | HLA-G(dist=4483),HLA-H(dist=52155) | - | - | - | - | - | - | 6 | - | - | - | - |
| rs17185566 | 6 | 29803523 | 0.68 | intergenic | HLA-G(dist=4624),HLA-H(dist=52014) | - | - | - | - | - | - | 6 | - | - | - | - |
| rs16896049 | 6 | 29803730 | 0.68 | intergenic | HLA-G(dist=4831),HLA-H(dist=51807) | - | - | - | - | - | - | 6 | - | - | - | - |
| rs16896052 | 6 | 29804176 | 0.68 | intergenic | HLA-G(dist=5277),HLA-H(dist=51361) | - | - | - | - | - | - | 7 | - | - | - | - |
| rs60690694 | 6 | 29804319 | 0.68 | intergenic | HLA-G(dist=5420),HLA-H(dist=51218) | - | - | - | - | - | - | 7 | - | - | - | - |
| rs62391986 | 6 | 29804454 | 0.68 | intergenic | HLA-G(dist=5555),HLA-H(dist=51083) | - | - | - | - | - | - | 6 | - | - | - | - |
| rs57066489 | 6 | 29805121 | 0.68 | intergenic | HLA-G(dist=6222),HLA-H(dist=50416) | - | - | - | - | - | - | 7 | - | - | - | - |
| rs57753710 | 6 | 29805276 | 0.68 | intergenic | HLA-G(dist=6377),HLA-H(dist=50261) | - | - | - | - | - | - | 7 | - | - | - | - |
| rs55936040 | 6 | 29805471 | 0.68 | intergenic | HLA-G(dist=6572),HLA-H(dist=50066) | - | - | - | - | - | - | 6 | - | - | - | - |
| rs200329695 | 6 | 29806460 | 0.63 | intergenic | HLA-G(dist=7561),HLA-H(dist=49077) | - | - | - | - | - | - | 7 | - | - | - | - |
| rs58571887 | 6 | 29807077 | 0.68 | intergenic | HLA-G(dist=8178),HLA-H(dist=48460) | - | - | - | - | - | - | 6 | - | - | - | - |
| rs55918403 | 6 | 29807591 | 0.68 | intergenic | HLA-G(dist=8692),HLA-H(dist=47946) | - | - | - | - | - | - | 7 | - | - | - | - |
| rs56061797 | 6 | 29807626 | 0.68 | intergenic | HLA-G(dist=8727),HLA-H(dist=47911) | - | - | - | - | - | - | 7 | - | - | - | - |
| rs55665721 | 6 | 29807775 | 0.68 | intergenic | HLA-G(dist=8876),HLA-H(dist=47762) | - | - | - | - | - | - | 5 | - | - | - | - |
| rs4314521 | 6 | 29809508 | 0.68 | intergenic | HLA-G(dist=10609),HLA-H(dist=46029) | - | - | - | - | - | - | 7 | - | - | - | - |
| rs3900927 | 6 | 29809579 | 0.68 | intergenic | HLA-G(dist=10680),HLA-H(dist=45958) | - | - | - | - | - | - | 5 | - | - | - | - |
| rs3900925 | 6 | 29809956 | 0.67 | intergenic | HLA-G(dist=11057),HLA-H(dist=45581) | - | - | - | - | - | - | 5 | - | - | - | - |
| rs56733837 | 6 | 29810447 | 0.67 | intergenic | HLA-G(dist=11548),HLA-H(dist=45090) | - | - | - | - | - | - | 6 | - | - | - | - |
| rs58414786 | 6 | 29810467 | 0.68 | intergenic | HLA-G(dist=11568),HLA-H(dist=45070) | - | - | - | - | - | - | 7 | - | - | - | - |
| rs58452318 | 6 | 29810496 | 0.67 | intergenic | HLA-G(dist=11597),HLA-H(dist=45041) | - | - | - | - | - | - | 6 | - | - | - | - |
| rs60167059 | 6 | 29810851 | 0.68 | intergenic | HLA-G(dist=11952),HLA-H(dist=44686) | - | - | - | - | - | - | 7 | - | - | - | - |
| rs62392488 | 6 | 29810877 | 0.68 | intergenic | HLA-G(dist=11978),HLA-H(dist=44660) | - | - | - | - | - | - | 6 | - | - | - | - |
| rs16896081 | 6 | 29811709 | 0.68 | intergenic | HLA-G(dist=12810),HLA-H(dist=43828) | - | - | - | - | - | - | 1c | - | - | - | - |
| rs4084097 | 6 | 29811750 | 0.68 | intergenic | HLA-G(dist=12851),HLA-H(dist=43787) | - | - | - | - | - | - | 1d | - | - | - | - |
| rs116773407 | 6 | 29811819 | 0.68 | intergenic | HLA-G(dist=12920),HLA-H(dist=43718) | - | - | - | - | - | - | 4 | - | - | - | - |
| rs36027729 | 6 | 29812255 | 0.68 | intergenic | HLA-G(dist=13356),HLA-H(dist=43282) | - | - | - | - | - | - | 6 | - | - | - | - |
| rs5013308 | 6 | 29812445 | 0.67 | intergenic | HLA-G(dist=13546),HLA-H(dist=43092) | - | Y | - | - | - | - | 2b | - | - | - | - |
| rs114853934 | 6 | 29812896 | 0.68 | intergenic | HLA-G(dist=13997),HLA-H(dist=42641) | - | Y | - | - | - | - | 7 | - | - | - | - |
| rs62392493 | 6 | 29812958 | 0.68 | intergenic | HLA-G(dist=14059),HLA-H(dist=42579) | - | Y | - | - | - | - | 5 | - | - | - | - |
| rs114583127 | 6 | 29813430 | 0.68 | intergenic | HLA-G(dist=14531),HLA-H(dist=42107) | - | Y | - | - | Y | - | 2b | - | - | - | - |
| rs115811566 | 6 | 29813489 | 0.68 | intergenic | HLA-G(dist=14590),HLA-H(dist=42048) | - | - | - | - | - | - | 4 | - | - | - | - |
| rs57595769 | 6 | 29813565 | 0.68 | intergenic | HLA-G(dist=14666),HLA-H(dist=41972) | - | - | - | - | - | - | 5 | - | - | - | - |
| rs528478845 | 6 | 29813788 | 0.68 | intergenic | HLA-G(dist=14889),HLA-H(dist=41749) | - | - | - | - | - | - | - | - | - | - | - |
| rs201505280 | 6 | 29813797 | 0.68 | intergenic | HLA-G(dist=14898),HLA-H(dist=41740) | - | - | - | - | - | - | 6 | - | - | - | - |
| rs531653219 | 6 | 29813798 | 0.68 | intergenic | HLA-G(dist=14899),HLA-H(dist=41739) | - | - | - | - | - | - | - | - | - | - | - |
| rs57581141 | 6 | 29813836 | 0.68 | intergenic | HLA-G(dist=14937),HLA-H(dist=41701) | - | - | - | - | - | - | 7 | - | - | - | - |
| rs58918579 | 6 | 29813855 | 0.68 | intergenic | HLA-G(dist=14956),HLA-H(dist=41682) | - | - | - | - | - | - | 6 | - | - | - | - |
| rs57803507 | 6 | 29813870 | 0.68 | intergenic | HLA-G(dist=14971),HLA-H(dist=41667) | - | - | - | - | - | - | 7 | - | - | - | - |
| rs4081563 | 6 | 29814364 | 0.68 | intergenic | HLA-G(dist=15465),HLA-H(dist=41173) | - | - | - | - | - | - | 7 | - | - | - | - |
| rs34663595 | 6 | 29814498 | 0.68 | intergenic | HLA-G(dist=15599),HLA-H(dist=41039) | - | - | - | - | - | - | 6 | - | - | - | - |
| rs3998738 | 6 | 29814553 | 0.68 | intergenic | HLA-G(dist=15654),HLA-H(dist=40984) | - | - | - | - | - | - | 6 | - | - | - | - |
| rs115460701 | 6 | 29814986 | 0.68 | intergenic | HLA-G(dist=16087),HLA-H(dist=40551) | - | - | - | - | - | - | 7 | - | - | - | - |
| rs548170078 | 6 | 29815057 | 0.68 | intergenic | HLA-G(dist=16158),HLA-H(dist=40480) | - | - | - | - | - | - | - | - | - | - | - |
| rs531065483 | 6 | 29815107 | 0.68 | intergenic | HLA-G(dist=16208),HLA-H(dist=40430) | - | - | - | - | - | - | - | - | - | - | - |
| rs28721753 | 6 | 29815694 | 0.68 | intergenic | HLA-G(dist=16795),HLA-H(dist=39843) | - | Y | - | - | - | - | 4 | - | - | - | - |
| rs28743486 | 6 | 29816041 | 0.68 | intergenic | HLA-G(dist=17142),HLA-H(dist=39496) | - | Y | Y | Y | Y | - | 4 | - | - | - | - |
| rs62392521 | 6 | 29816289 | 0.68 | intergenic | HLA-G(dist=17390),HLA-H(dist=39248) | - | Y | Y | - | - | - | 4 | - | - | - | - |
| rs56271566 | 6 | 29817086 | 0.68 | intergenic | HLA-G(dist=18187),HLA-H(dist=38451) | - | - | - | - | - | - | 6 | - | - | - | - |
| rs62392523 | 6 | 29817253 | 0.69 | intergenic | HLA-G(dist=18354),HLA-H(dist=38284) | - | - | - | - | - | - | 6 | - | - | - | - |
| rs62392524 | 6 | 29817288 | 0.68 | intergenic | HLA-G(dist=18389),HLA-H(dist=38249) | - | - | - | - | - | - | 7 | - | - | - | - |
| rs3886811 | 6 | 29817379 | 0.69 | intergenic | HLA-G(dist=18480),HLA-H(dist=38158) | - | - | - | - | - | - | 5 | - | - | - | - |
| rs141551310 | 6 | 29817656 | 0.69 | intergenic | HLA-G(dist=18757),HLA-H(dist=37881) | - | - | - | - | - | - | 2b | - | - | - | - |
| rs62392525 | 6 | 29819330 | 0.69 | intergenic | HLA-G(dist=20431),HLA-H(dist=36207) | - | - | - | - | - | - | 4 | - | - | - | - |
| rs17179199 | 6 | 29820977 | 0.65 | intergenic | HLA-G(dist=22078),HLA-H(dist=34560) | - | - | - | - | - | - | 7 | - | - | - | - |
| rs17185580 | 6 | 29821018 | 0.65 | intergenic | HLA-G(dist=22119),HLA-H(dist=34519) | - | - | - | - | - | - | 5 | - | - | - | - |
| rs17179213 | 6 | 29821221 | 0.65 | intergenic | HLA-G(dist=22322),HLA-H(dist=34316) | - | - | - | - | Y | - | 2c | - | - | - | - |
| rs17185636 | 6 | 29821439 | 0.65 | intergenic | HLA-G(dist=22540),HLA-H(dist=34098) | - | - | - | - | - | - | 3a | - | - | - | - |
| rs17179277 | 6 | 29821683 | 0.65 | intergenic | HLA-G(dist=22784),HLA-H(dist=33854) | - | - | - | - | - | - | 3a | - | - | - | - |
| rs77003338 | 6 | 29822200 | 0.65 | intergenic | HLA-G(dist=23301),HLA-H(dist=33337) | - | - | - | - | - | - | 6 | - | - | - | - |
| rs61162280 | 6 | 29822726 | 0.65 | intergenic | HLA-G(dist=23827),HLA-H(dist=32811) | - | - | - | - | - | - | 7 | - | - | - | - |
| rs16896139 | 6 | 29823191 | 0.69 | intergenic | HLA-G(dist=24292),HLA-H(dist=32346) | - | - | - | - | - | - | 5 | - | - | - | - |
| rs56068459 | 6 | 29826149 | 0.68 | intergenic | HLA-G(dist=27250),HLA-H(dist=29388) | - | - | - | - | - | - | 7 | - | - | - | - |
| rs55662140 | 6 | 29826549 | 0.68 | intergenic | HLA-G(dist=27650),HLA-H(dist=28988) | - | - | - | - | - | - | 6 | - | - | - | - |
| rs17185692 | 6 | 29828316 | 0.65 | intergenic | HLA-G(dist=29417),HLA-H(dist=27221) | - | - | - | - | - | - | 6 | - | - | - | - |
| rs16896166 | 6 | 29829450 | 0.68 | intergenic | HLA-G(dist=30551),HLA-H(dist=26087) | - | - | - | - | - | - | 4 | - | - | - | - |
| rs62391661 | 6 | 29829494 | 0.68 | intergenic | HLA-G(dist=30595),HLA-H(dist=26043) | - | - | - | - | - | - | 4 | - | - | - | - |
| rs146895680 | 6 | 29829853 | 0.66 | intergenic | HLA-G(dist=30954),HLA-H(dist=25684) | - | - | - | - | - | - | 5 | - | - | - | - |
| rs151188422 | 6 | 29830777 | 0.69 | intergenic | HLA-G(dist=31878),HLA-H(dist=24760) | - | - | - | - | - | - | 6 | - | - | - | - |
| rs62391663 | 6 | 29831657 | 0.66 | intergenic | HLA-G(dist=32758),HLA-H(dist=23880) | - | - | - | - | - | - | 5 | - | - | - | - |
| rs116742675 | 6 | 29832497 | 0.69 | intergenic | HLA-G(dist=33598),HLA-H(dist=23040) | - | - | - | - | - | - | 5 | - | - | - | - |
| rs147147213 | 6 | 29832652 | 0.64 | intergenic | HLA-G(dist=33753),HLA-H(dist=22885) | - | - | - | - | - | - | 6 | - | - | - | - |
| rs138395585 | 6 | 29832656 | 0.64 | intergenic | HLA-G(dist=33757),HLA-H(dist=22881) | - | - | - | - | - | - | 6 | - | - | - | - |
| rs534593564 | 6 | 29832659 | 0.63 | intergenic | HLA-G(dist=33760),HLA-H(dist=22878) | - | - | - | - | - | - | - | - | - | - | - |
| rs557912353 | 6 | 29832661 | 0.64 | intergenic | HLA-G(dist=33762),HLA-H(dist=22876) | - | - | - | - | - | - | - | - | - | - | - |
| rs571347799 | 6 | 29832662 | 0.63 | intergenic | HLA-G(dist=33763),HLA-H(dist=22875) | - | - | - | - | - | - | - | - | - | - | - |
| rs62391664 | 6 | 29833109 | 0.68 | intergenic | HLA-G(dist=34210),HLA-H(dist=22428) | - | - | - | - | - | - | 5 | - | - | - | - |
| rs55773721 | 6 | 29835346 | 0.69 | intergenic | HLA-G(dist=36447),HLA-H(dist=20191) | - | - | - | - | - | - | 5 | - | - | - | - |
| rs62391710 | 6 | 29836018 | 0.69 | intergenic | HLA-G(dist=37119),HLA-H(dist=19519) | - | - | - | - | - | - | 5 | - | - | - | - |
| rs56012230 | 6 | 29836270 | 0.69 | intergenic | HLA-G(dist=37371),HLA-H(dist=19267) | - | - | - | - | - | - | 6 | - | - | - | - |
| rs56358953 | 6 | 29836817 | 0.69 | intergenic | HLA-G(dist=37918),HLA-H(dist=18720) | - | - | - | - | - | - | 6 | - | - | - | - |
| rs56129366 | 6 | 29836825 | 0.69 | intergenic | HLA-G(dist=37926),HLA-H(dist=18712) | - | - | - | - | - | - | 7 | - | - | - | - |
| rs55764151 | 6 | 29836951 | 0.69 | intergenic | HLA-G(dist=38052),HLA-H(dist=18586) | - | - | - | - | - | - | 5 | - | - | - | - |
| rs3893468 | 6 | 29837882 | 0.69 | intergenic | HLA-G(dist=38983),HLA-H(dist=17655) | - | - | - | - | - | - | 7 | - | - | - | - |
| rs55944958 | 6 | 29837940 | 0.69 | intergenic | HLA-G(dist=39041),HLA-H(dist=17597) | - | - | - | - | - | - | 7 | - | - | - | - |
| rs62391716 | 6 | 29839097 | 0.69 | intergenic | HLA-G(dist=40198),HLA-H(dist=16440) | - | - | - | - | - | - | 7 | - | - | - | - |
| rs74961904 | 6 | 29839472 | 0.69 | intergenic | HLA-G(dist=40573),HLA-H(dist=16065) | - | - | - | - | - | - | 6 | - | - | - | - |
| rs77816199 | 6 | 29839473 | 0.69 | intergenic | HLA-G(dist=40574),HLA-H(dist=16064) | - | - | - | - | - | - | 6 | - | - | - | - |
| rs78304580 | 6 | 29839502 | 0.69 | intergenic | HLA-G(dist=40603),HLA-H(dist=16035) | - | - | - | - | - | - | 6 | - | - | - | - |
| rs62391717 | 6 | 29840298 | 0.69 | intergenic | HLA-G(dist=41399),HLA-H(dist=15239) | - | - | - | - | - | - | 6 | - | - | - | - |
| rs60859898 | 6 | 29840952 | 0.69 | intergenic | HLA-G(dist=42053),HLA-H(dist=14585) | - | - | - | - | - | - | 7 | - | - | - | - |
| rs55745323 | 6 | 29841510 | 0.69 | intergenic | HLA-G(dist=42611),HLA-H(dist=14027) | - | - | - | - | - | - | 7 | - | - | - | - |
| rs62391719 | 6 | 29841569 | 0.69 | intergenic | HLA-G(dist=42670),HLA-H(dist=13968) | - | - | - | - | - | - | 5 | - | - | - | - |
| rs62391720 | 6 | 29841590 | 0.69 | intergenic | HLA-G(dist=42691),HLA-H(dist=13947) | - | - | - | - | - | - | 5 | - | - | - | - |
| rs62391721 | 6 | 29841611 | 0.69 | intergenic | HLA-G(dist=42712),HLA-H(dist=13926) | - | - | - | - | - | - | 5 | - | - | - | - |
| rs55909010 | 6 | 29842577 | 0.69 | intergenic | HLA-G(dist=43678),HLA-H(dist=12960) | - | - | - | - | - | - | 7 | - | - | - | - |
| rs56212122 | 6 | 29843023 | 0.69 | intergenic | HLA-G(dist=44124),HLA-H(dist=12514) | - | - | - | - | - | - | 6 | - | - | - | - |
| rs56148555 | 6 | 29843092 | 0.69 | intergenic | HLA-G(dist=44193),HLA-H(dist=12445) | - | - | - | - | - | - | 6 | - | - | - | - |
| rs62389282 | 6 | 29843544 | 0.64 | intergenic | HLA-G(dist=44645),HLA-H(dist=11993) | - | - | - | - | - | - | 7 | - | - | - | - |
| rs80076845 | 6 | 29844629 | 0.64 | intergenic | HLA-G(dist=45730),HLA-H(dist=10908) | - | - | - | - | - | - | 7 | - | - | - | - |
| rs62389286 | 6 | 29844885 | 0.64 | intergenic | HLA-G(dist=45986),HLA-H(dist=10652) | - | - | - | - | - | - | 7 | - | - | - | - |
| rs59370562 | 6 | 29845006 | 0.64 | intergenic | HLA-G(dist=46107),HLA-H(dist=10531) | - | - | - | - | - | - | 6 | - | - | - | - |
| rs57079379 | 6 | 29845134 | 0.64 | intergenic | HLA-G(dist=46235),HLA-H(dist=10403) | - | - | - | - | - | - | 6 | - | - | - | - |
| rs62389313 | 6 | 29846315 | 0.64 | intergenic | HLA-G(dist=47416),HLA-H(dist=9222) | - | - | - | - | - | - | 7 | - | - | - | - |
| rs62389314 | 6 | 29846644 | 0.63 | intergenic | HLA-G(dist=47745),HLA-H(dist=8893) | - | - | - | - | - | - | 6 | - | - | - | - |
| rs62389315 | 6 | 29846751 | 0.64 | intergenic | HLA-G(dist=47852),HLA-H(dist=8786) | - | - | - | - | - | - | 7 | - | - | - | - |
| rs62389317 | 6 | 29846936 | 0.64 | intergenic | HLA-G(dist=48037),HLA-H(dist=8601) | - | - | - | - | - | - | 6 | - | - | - | - |
| rs62389318 | 6 | 29847042 | 0.64 | intergenic | HLA-G(dist=48143),HLA-H(dist=8495) | - | - | - | - | - | - | 6 | - | - | - | - |
| rs145068299 | 6 | 29847065 | 0.64 | intergenic | HLA-G(dist=48166),HLA-H(dist=8472) | - | - | - | - | - | - | 7 | - | - | - | - |
| rs192274703 | 6 | 29847068 | 0.64 | intergenic | HLA-G(dist=48169),HLA-H(dist=8469) | - | - | - | - | - | - | 7 | - | - | - | - |
| rs62389319 | 6 | 29847388 | 0.76 | intergenic | HLA-G(dist=48489),HLA-H(dist=8149) | - | - | - | - | - | - | 6 | - | - | - | - |
| rs56239938 | 6 | 29847440 | 0.64 | intergenic | HLA-G(dist=48541),HLA-H(dist=8097) | - | - | - | - | - | - | 6 | - | - | - | - |
| rs56097399 | 6 | 29847649 | 0.64 | intergenic | HLA-G(dist=48750),HLA-H(dist=7888) | - | - | - | - | - | - | 7 | - | - | - | - |
| rs202049838 | 6 | 29847915 | 0.76 | intergenic | HLA-G(dist=49016),HLA-H(dist=7622) | - | - | - | - | - | - | 7 | - | - | - | - |
| rs59828806 | 6 | 29848863 | 0.64 | intergenic | HLA-G(dist=49964),HLA-H(dist=6674) | - | - | - | - | - | - | 7 | - | - | - | - |
| rs57397323 | 6 | 29848868 | 0.64 | intergenic | HLA-G(dist=49969),HLA-H(dist=6669) | - | - | - | - | - | - | 7 | - | - | - | - |
| rs55869449 | 6 | 29849059 | 0.64 | intergenic | HLA-G(dist=50160),HLA-H(dist=6478) | - | - | - | - | - | - | 6 | - | - | - | - |
| rs540117519 | 6 | 29849532 | 0.68 | intergenic | HLA-G(dist=50633),HLA-H(dist=6005) | - | - | - | - | - | - | - | - | - | - | - |
| rs62389327 | 6 | 29850102 | 0.69 | intergenic | HLA-G(dist=51203),HLA-H(dist=5435) | - | - | - | - | - | - | 7 | - | - | - | - |
| rs62389328 | 6 | 29850111 | 0.69 | intergenic | HLA-G(dist=51212),HLA-H(dist=5426) | - | - | - | - | - | - | 7 | - | - | - | - |
| rs146235176 | 6 | 29850550 | 0.69 | intergenic | HLA-G(dist=51651),HLA-H(dist=4987) | - | - | - | - | - | - | 7 | - | - | - | - |
| rs62389329 | 6 | 29850849 | 0.69 | intergenic | HLA-G(dist=51950),HLA-H(dist=4688) | - | - | - | - | - | - | 7 | - | - | - | - |
| rs150486315 | 6 | 29851150 | 0.66 | intergenic | HLA-G(dist=52251),HLA-H(dist=4387) | - | - | - | - | - | - | 7 | - | - | - | - |
| rs146028785 | 6 | 29851248 | 0.69 | intergenic | HLA-G(dist=52349),HLA-H(dist=4289) | - | - | - | - | - | - | 6 | - | - | - | - |
| rs55882115 | 6 | 29851501 | 0.62 | intergenic | HLA-G(dist=52602),HLA-H(dist=4036) | - | - | - | - | - | - | 7 | - | - | - | - |
| rs56174454 | 6 | 29851740 | 0.62 | intergenic | HLA-G(dist=52841),HLA-H(dist=3797) | - | - | - | - | - | - | 5 | - | - | - | - |
| rs55947552 | 6 | 29851973 | 0.62 | intergenic | HLA-G(dist=53074),HLA-H(dist=3564) | - | - | - | - | - | - | 5 | - | - | - | - |
| rs62389331 | 6 | 29852362 | 0.62 | intergenic | HLA-G(dist=53463),HLA-H(dist=3175) | - | - | - | - | - | - | 5 | - | - | - | - |
| rs56088713 | 6 | 29853133 | 0.62 | intergenic | HLA-G(dist=54234),HLA-H(dist=2404) | - | - | - | - | - | - | 5 | - | - | - | - |
| rs56389301 | 6 | 29853434 | 0.62 | intergenic | HLA-G(dist=54535),HLA-H(dist=2103) | - | - | - | - | - | - | 7 | - | - | - | - |
| rs2893994 | 6 | 29853621 | 0.62 | intergenic | HLA-G(dist=54722),HLA-H(dist=1916) | - | - | - | - | - | - | 7 | - | - | - | - |
| rs2394232 | 6 | 29853682 | 0.62 | intergenic | HLA-G(dist=54783),HLA-H(dist=1855) | - | - | - | - | - | - | 5 | - | - | - | - |
| rs2394235 | 6 | 29854172 | 0.62 | intergenic | HLA-G(dist=55273),HLA-H(dist=1365) | - | - | - | - | - | - | 5 | - | - | - | - |
| rs5875214 | 6 | 29854457 | 0.62 | intergenic | HLA-G(dist=55558),HLA-H(dist=1080) | - | - | - | - | - | - | 6 | - | - | - | - |
| rs2394241 | 6 | 29854506 | 0.62 | intergenic | HLA-G(dist=55607),HLA-H(dist=1031) | - | - | - | - | - | - | 5 | - | - | - | - |
| rs2394242 | 6 | 29854516 | 0.62 | intergenic | HLA-G(dist=55617),HLA-H(dist=1021) | - | - | - | - | - | - | 5 | - | - | - | - |
| rs114222883 | 6 | 29854585 | 0.62 | upstream | HLA-H | - | - | - | - | - | - | 5 | - | - | - | - |
| rs62389356 | 6 | 29855733 | 0.64 | ncRNA_exonic | HLA-H | - | - | - | - | - | - | 4 | - | - | - | - |
| rs62389357 | 6 | 29855902 | 0.63 | ncRNA_exonic | HLA-H | - | - | Y | - | - | - | 4 | - | - | - | - |
| rs4412230 | 6 | 29856109 | 0.62 | ncRNA_intronic | HLA-H | - | - | - | - | - | - | 2b | - | - | - | - |
| rs5012016 | 6 | 29856199 | 0.62 | ncRNA_intronic | HLA-H | - | - | - | - | - | - | 2b | - | - | - | - |
| rs5012015 | 6 | 29856204 | 0.61 | ncRNA_intronic | HLA-H | - | - | - | - | - | - | 2b | - | - | - | - |
| rs55682221 | 6 | 29856546 | 0.62 | ncRNA_intronic | HLA-H | - | - | - | - | - | - | 4 | - | - | - | - |
| rs114986421 | 6 | 29856965 | 0.62 | ncRNA_intronic | HLA-H | - | - | - | - | - | - | 5 | - | - | - | - |
| rs62389359 | 6 | 29857021 | 0.61 | ncRNA_intronic | HLA-H | - | - | - | - | - | - | 5 | - | - | - | - |
| rs144383179 | 6 | 29858712 | 0.62 | ncRNA_exonic | HLA-H | - | - | - | - | - | - | 5 | - | - | - | - |
| rs62390860 | 6 | 29858903 | 0.62 | downstream | HLA-H | - | - | - | - | - | - | 5 | - | - | - | - |
| rs115064987 | 6 | 29859055 | 0.61 | downstream | HLA-H | - | - | - | - | - | - | 5 | - | - | - | - |
| rs116750684 | 6 | 29859083 | 0.61 | downstream | HLA-H | - | - | - | - | - | - | 7 | - | - | - | - |
| rs62390861 | 6 | 29859954 | 0.61 | intergenic | HLA-H(dist=1097),HCG4B(dist=32415) | - | - | - | - | - | - | 5 | - | - | - | - |
| rs62390862 | 6 | 29860039 | 0.61 | intergenic | HLA-H(dist=1182),HCG4B(dist=32330) | - | - | - | - | - | - | 5 | - | - | - | - |
| rs62390893 | 6 | 29860250 | 0.61 | intergenic | HLA-H(dist=1393),HCG4B(dist=32119) | - | - | - | - | - | - | 5 | - | - | - | - |
| rs62390895 | 6 | 29860868 | 0.61 | intergenic | HLA-H(dist=2011),HCG4B(dist=31501) | - | - | - | - | - | - | 5 | - | - | - | - |
| rs62390897 | 6 | 29861930 | 0.61 | intergenic | HLA-H(dist=3073),HCG4B(dist=30439) | - | - | - | - | - | - | 6 | - | - | - | - |
| rs62390898 | 6 | 29861983 | 0.61 | intergenic | HLA-H(dist=3126),HCG4B(dist=30386) | - | - | - | - | - | - | 6 | - | - | - | - |
| rs59500422 | 6 | 29862465 | 0.61 | intergenic | HLA-H(dist=3608),HCG4B(dist=29904) | - | - | - | - | - | - | 6 | - | - | - | - |
| rs56110645 | 6 | 29863017 | 0.61 | intergenic | HLA-H(dist=4160),HCG4B(dist=29352) | - | - | - | - | - | - | 6 | - | - | - | - |
| rs56148889 | 6 | 29863414 | 0.61 | intergenic | HLA-H(dist=4557),HCG4B(dist=28955) | - | - | - | - | - | - | 7 | - | - | - | - |
| rs192301647 | 6 | 29863729 | 0.61 | intergenic | HLA-H(dist=4872),HCG4B(dist=28640) | - | - | - | - | - | - | 7 | - | - | - | - |
| rs190636413 | 6 | 29863777 | 0.61 | intergenic | HLA-H(dist=4920),HCG4B(dist=28592) | - | - | - | - | - | - | 7 | - | - | - | - |
| rs62390901 | 6 | 29864022 | 0.62 | intergenic | HLA-H(dist=5165),HCG4B(dist=28347) | - | - | - | - | - | - | 6 | - | - | - | - |
| rs147012787 | 6 | 29864145 | 0.61 | intergenic | HLA-H(dist=5288),HCG4B(dist=28224) | - | - | - | - | - | - | 6 | - | - | - | - |
| rs114298287 | 6 | 29865311 | 0.61 | intergenic | HLA-H(dist=6454),HCG4B(dist=27058) | - | - | - | - | - | - | 7 | - | - | - | - |
| rs62390903 | 6 | 29866338 | 0.61 | intergenic | HLA-H(dist=7481),HCG4B(dist=26031) | - | - | - | - | - | - | 5 | - | - | - | - |
| rs62390904 | 6 | 29866388 | 0.61 | intergenic | HLA-H(dist=7531),HCG4B(dist=25981) | - | - | - | - | - | - | 5 | - | - | - | - |
| rs56054591 | 6 | 29867051 | 0.61 | intergenic | HLA-H(dist=8194),HCG4B(dist=25318) | - | - | - | - | - | - | 7 | - | - | - | - |
| rs55725536 | 6 | 29867181 | 0.61 | intergenic | HLA-H(dist=8324),HCG4B(dist=25188) | - | - | - | - | - | - | 5 | - | - | - | - |
| rs116514561 | 6 | 29867616 | 0.61 | intergenic | HLA-H(dist=8759),HCG4B(dist=24753) | - | - | - | - | - | - | 6 | - | - | - | - |
| rs567365886 | 6 | 29867895 | 0.69 | intergenic | HLA-H(dist=9038),HCG4B(dist=24474) | - | - | - | - | - | - | - | - | - | - | - |
| rs9259514 | 6 | 29868208 | 0.61 | intergenic | HLA-H(dist=9351),HCG4B(dist=24161) | - | - | - | - | - | - | 6 | - | - | - | - |
| rs114238601 | 6 | 29868392 | 0.61 | intergenic | HLA-H(dist=9535),HCG4B(dist=23977) | - | - | - | - | - | - | 6 | - | - | - | - |
| rs62390911 | 6 | 29868633 | 0.61 | intergenic | HLA-H(dist=9776),HCG4B(dist=23736) | - | - | - | - | - | - | 5 | - | - | - | - |
| rs56169793 | 6 | 29870100 | 0.61 | intergenic | HLA-H(dist=11243),HCG4B(dist=22269) | - | - | - | - | - | - | 7 | - | - | - | - |
| rs2524034 | 6 | 29876639 | 0.67 | intergenic | HLA-H(dist=17782),HCG4B(dist=15730) | - | - | - | - | - | - | 5 | - | - | - | - |
| rs4599690 | 6 | 29880640 | 0.79 | intergenic | HLA-H(dist=21783),HCG4B(dist=11729) | - | - | - | - | - | - | 5 | - | - | - | - |
| rs55748016 | 6 | 29881424 | 0.79 | intergenic | HLA-H(dist=22567),HCG4B(dist=10945) | - | - | - | - | - | - | 5 | - | - | - | - |
| rs1991956 | 6 | 29884001 | 0.79 | intergenic | HLA-H(dist=25144),HCG4B(dist=8368) | - | - | - | - | - | - | 6 | - | - | - | - |
| rs1991955 | 6 | 29884066 | 0.79 | intergenic | HLA-H(dist=25209),HCG4B(dist=8303) | - | - | - | - | - | - | 6 | - | - | - | - |
| rs55676227 | 6 | 29892163 | 0.79 | downstream | HCG4B | - | - | - | - | - | - | 5 | - | - | - | - |
| rs1056243 | 6 | 29893394 | 0.79 | ncRNA_exonic | HCG4B | - | - | - | - | - | - | 4 | - | - | - | - |
| rs114248517 | 6 | 29893512 | 0.79 | ncRNA_exonic | HCG4B | - | - | - | - | - | - | 5 | - | - | - | - |
| rs114908267 | 6 | 29893586 | 0.79 | ncRNA_exonic | HCG4B | - | - | - | - | - | - | 4 | - | - | - | - |
| rs116500576 | 6 | 29893606 | 0.79 | ncRNA_exonic | HCG4B | - | - | - | - | - | - | 2b | - | - | - | - |
| rs533742533 | 6 | 29893624 | 0.89 | ncRNA_exonic | HCG4B | - | - | - | - | - | - | - | - | - | - | - |
| rs142964655 | 6 | 29893679 | 0.79 | ncRNA_exonic | HCG4B | - | - | - | - | - | - | 4 | - | - | - | - |
| rs62389590 | 6 | 29894017 | 0.80 | ncRNA_exonic | HCG4B | - | - | Y | - | - | - | 2b | - | - | - | - |
| rs62389591 | 6 | 29894030 | 0.79 | ncRNA_exonic | HCG4B | - | - | - | - | - | - | 2a | - | - | - | - |
| rs62389592 | 6 | 29894139 | 0.80 | ncRNA_exonic | HCG4B | - | - | - | - | - | - | 4 | - | - | - | - |
| rs2240073 | 6 | 29894250 | 0.79 | ncRNA_exonic | HCG4B | - | - | - | - | - | - | 2b | - | - | - | - |
| rs2074485 | 6 | 29894269 | 0.79 | ncRNA_exonic | HCG4B | - | - | - | - | - | - | 4 | - | - | - | - |
| rs191134254 | 6 | 29894332 | 0.79 | ncRNA_exonic | HCG4B | - | - | Y | - | - | - | 2b | - | - | - | - |
| rs2240072 | 6 | 29894334 | 0.79 | ncRNA_exonic | HCG4B | - | - | Y | - | - | - | 2b | - | - | - | - |
| rs201884321 | 6 | 29894349 | 0.80 | ncRNA_exonic | HCG4B | - | - | Y | - | - | - | 2b | - | - | - | - |
| rs114028743 | 6 | 29894406 | 0.79 | ncRNA_exonic | HCG4B | - | - | Y | - | - | - | 4 | - | - | - | - |
| rs114779824 | 6 | 29894407 | 0.79 | ncRNA_exonic | HCG4B | - | - | Y | - | - | - | 4 | - | - | - | - |
| rs62390374 | 6 | 29894523 | 0.78 | ncRNA_exonic | HCG4B | - | - | Y | - | - | - | 4 | - | - | - | - |
| rs116296604 | 6 | 29894567 | 0.78 | ncRNA_exonic | HCG4B | - | - | Y | - | - | - | 4 | - | - | - | - |
| rs116078621 | 6 | 29894603 | 0.78 | ncRNA_exonic | HCG4B | - | - | Y | - | - | - | 4 | - | - | - | - |
| rs139386564 | 6 | 29894636 | 0.79 | ncRNA_exonic | HCG4B | - | - | Y | - | - | - | 4 | - | - | - | - |
| rs117527351 | 6 | 29894756 | 0.79 | ncRNA_exonic | HCG4B | - | - | Y | - | - | - | 4 | - | - | - | - |
| rs188560568 | 6 | 29894914 | 0.79 | ncRNA_exonic | HCG4B | - | - | Y | Y | - | - | 2a | - | - | - | - |
| rs62390375 | 6 | 29894966 | 0.79 | ncRNA_exonic | HCG4B | - | - | Y | Y | - | - | 4 | - | - | - | - |
| rs62390376 | 6 | 29895005 | 0.79 | upstream | HCG4B | - | - | Y | - | - | - | 4 | - | - | - | - |
| rs62390377 | 6 | 29895039 | 0.79 | upstream | HCG4B | - | - | Y | - | - | - | 4 | - | - | - | - |
| rs62390378 | 6 | 29895334 | 0.79 | upstream | HCG4B | - | - | Y | - | - | - | 2b | - | - | - | - |
| rs62390379 | 6 | 29895472 | 0.79 | upstream | HCG4B | - | - | Y | - | - | - | 4 | - | - | - | - |
| rs144932908 | 6 | 29895536 | 0.79 | upstream | HCG4B | - | - | - | - | - | - | 5 | - | - | - | - |
| rs116377575 | 6 | 29895551 | 0.79 | upstream | HCG4B | - | - | - | - | - | - | 5 | - | - | - | - |
| rs368444991 | 6 | 29895566 | 0.79 | upstream | HCG4B | - | - | - | - | - | - | 5 | - | - | - | - |
| rs143066895 | 6 | 29895628 | 0.78 | upstream | HCG4B | - | - | - | - | - | - | 5 | - | - | - | - |
| rs148229033 | 6 | 29895684 | 0.78 | upstream | HCG4B | - | - | - | - | - | - | 5 | - | - | - | - |
| rs140219525 | 6 | 29895700 | 0.78 | upstream | HCG4B | - | - | - | - | - | - | 5 | - | - | - | - |
| rs138034172 | 6 | 29895772 | 0.78 | upstream | HCG4B | - | - | - | - | - | - | 7 | - | - | - | - |
| rs142655476 | 6 | 29895782 | 0.78 | upstream | HCG4B | - | - | - | - | - | - | 7 | - | - | - | - |
| rs144933869 | 6 | 29895788 | 0.78 | upstream | HCG4B | - | - | - | - | - | - | 7 | - | - | - | - |
| rs138703942 | 6 | 29895798 | 0.78 | upstream | HCG4B | - | - | - | - | - | - | 7 | - | - | - | - |
| rs568936455 | 6 | 29895823 | 0.78 | upstream | HCG4B | - | - | - | - | - | - | - | - | - | - | - |
| rs372834482 | 6 | 29895826 | 0.81 | upstream | HCG4B | - | - | - | - | - | - | 5 | - | - | - | - |
| rs56263834 | 6 | 29896125 | 0.78 | intergenic | HCG4B(dist=1133),HLA-A(dist=14122) | - | - | - | - | - | - | 6 | - | - | - | - |
| rs55803658 | 6 | 29896128 | 0.79 | intergenic | HCG4B(dist=1136),HLA-A(dist=14119) | - | - | - | - | - | - | 6 | - | - | - | - |
| rs143421979 | 6 | 29896210 | 0.79 | intergenic | HCG4B(dist=1218),HLA-A(dist=14037) | - | - | - | - | - | - | 6 | - | - | - | - |
| rs55795233 | 6 | 29896257 | 0.80 | intergenic | HCG4B(dist=1265),HLA-A(dist=13990) | - | - | - | - | - | - | 7 | - | - | - | - |
| rs56285764 | 6 | 29896291 | 0.80 | intergenic | HCG4B(dist=1299),HLA-A(dist=13956) | - | - | - | - | - | - | 5 | - | - | - | - |
| rs56201330 | 6 | 29897030 | 0.79 | intergenic | HCG4B(dist=2038),HLA-A(dist=13217) | - | - | - | - | - | - | 6 | - | - | - | - |
| rs116758941 | 6 | 29897301 | 0.80 | intergenic | HCG4B(dist=2309),HLA-A(dist=12946) | - | - | - | - | - | - | 5 | - | - | - | - |
| rs114934293 | 6 | 29897322 | 0.80 | intergenic | HCG4B(dist=2330),HLA-A(dist=12925) | - | - | - | - | - | - | 5 | - | - | - | - |
| rs201955922 | 6 | 29897416 | 0.79 | intergenic | HCG4B(dist=2424),HLA-A(dist=12831) | - | - | - | - | - | - | 5 | - | - | - | - |
| rs200431678 | 6 | 29897417 | 0.79 | intergenic | HCG4B(dist=2425),HLA-A(dist=12830) | - | - | - | - | - | - | 5 | - | - | - | - |
| rs117064855 | 6 | 29897422 | 0.79 | intergenic | HCG4B(dist=2430),HLA-A(dist=12825) | - | - | - | - | - | - | 5 | - | - | - | - |
| rs115901917 | 6 | 29897693 | 0.90 | intergenic | HCG4B(dist=2701),HLA-A(dist=12554) | - | - | - | - | - | - | 5 | - | - | - | - |
| rs62390400 | 6 | 29897790 | 0.78 | intergenic | HCG4B(dist=2798),HLA-A(dist=12457) | - | - | - | - | - | - | 5 | - | - | - | - |
| rs139065131 | 6 | 29897868 | 0.78 | intergenic | HCG4B(dist=2876),HLA-A(dist=12379) | - | - | - | - | - | - | 5 | - | - | - | - |
| rs62390401 | 6 | 29898219 | 0.78 | intergenic | HCG4B(dist=3227),HLA-A(dist=12028) | - | - | - | - | - | - | 5 | - | - | - | - |
| rs80289233 | 6 | 29898293 | 0.78 | intergenic | HCG4B(dist=3301),HLA-A(dist=11954) | - | - | - | - | - | - | 5 | - | - | - | - |
| rs76185263 | 6 | 29898389 | 0.78 | intergenic | HCG4B(dist=3397),HLA-A(dist=11858) | - | - | - | - | - | - | 6 | - | - | - | - |
| rs140343967 | 6 | 29898436 | 0.78 | intergenic | HCG4B(dist=3444),HLA-A(dist=11811) | - | - | - | - | - | - | 7 | - | - | - | - |
| rs62390402 | 6 | 29898499 | 0.78 | intergenic | HCG4B(dist=3507),HLA-A(dist=11748) | - | - | - | - | - | - | 7 | - | - | - | - |
| rs62390403 | 6 | 29898704 | 0.72 | intergenic | HCG4B(dist=3712),HLA-A(dist=11543) | - | - | - | - | - | - | 6 | - | - | - | - |
| rs114499319 | 6 | 29898759 | 0.78 | intergenic | HCG4B(dist=3767),HLA-A(dist=11488) | - | - | - | - | - | - | 6 | - | - | - | - |
| rs115998337 | 6 | 29898818 | 0.78 | intergenic | HCG4B(dist=3826),HLA-A(dist=11429) | - | - | - | - | - | - | 7 | - | - | - | - |
| rs4087726 | 6 | 29898897 | 0.78 | intergenic | HCG4B(dist=3905),HLA-A(dist=11350) | - | - | - | - | - | - | 6 | - | - | - | - |
| rs4087725 | 6 | 29898962 | 0.78 | intergenic | HCG4B(dist=3970),HLA-A(dist=11285) | - | - | - | - | - | - | 7 | - | - | - | - |
| rs4087724 | 6 | 29898991 | 0.78 | intergenic | HCG4B(dist=3999),HLA-A(dist=11256) | - | - | - | - | - | - | 5 | - | - | - | - |
| rs4087723 | 6 | 29899076 | 0.72 | intergenic | HCG4B(dist=4084),HLA-A(dist=11171) | - | - | - | - | - | - | 5 | - | - | - | - |
| rs200168988 | 6 | 29899871 | 0.69 | intergenic | HCG4B(dist=4879),HLA-A(dist=10376) | - | - | - | - | - | - | 7 | - | - | - | - |
| rs62388760 | 6 | 29900075 | 0.70 | intergenic | HCG4B(dist=5083),HLA-A(dist=10172) | - | - | - | - | - | - | 7 | - | - | - | - |
| rs62388761 | 6 | 29900094 | 0.70 | intergenic | HCG4B(dist=5102),HLA-A(dist=10153) | - | - | - | - | - | - | 6 | - | - | - | - |
| rs569707517 | 6 | 29900554 | 0.89 | intergenic | HCG4B(dist=5562),HLA-A(dist=9693) | - | - | - | - | - | - | - | - | - | - | - |
| rs535601917 | 6 | 29900555 | 0.89 | intergenic | HCG4B(dist=5563),HLA-A(dist=9692) | - | - | - | - | - | - | - | - | - | - | - |
| rs62388764 | 6 | 29901604 | 0.79 | intergenic | HCG4B(dist=6612),HLA-A(dist=8643) | - | - | - | - | - | - | 5 | - | - | - | - |
| rs61180635 | 6 | 29902168 | 0.79 | intergenic | HCG4B(dist=7176),HLA-A(dist=8079) | - | - | - | - | - | - | 5 | - | - | - | - |
| rs58309428 | 6 | 29902293 | 0.79 | intergenic | HCG4B(dist=7301),HLA-A(dist=7954) | - | - | - | - | - | - | 5 | - | - | - | - |
| rs200767773 | 6 | 29902489 | 0.80 | intergenic | HCG4B(dist=7497),HLA-A(dist=7758) | - | - | - | - | - | - | 6 | - | - | - | - |
| rs200273569 | 6 | 29903739 | 0.84 | intergenic | HCG4B(dist=8747),HLA-A(dist=6508) | - | - | - | - | - | - | 6 | - | - | - | - |
| rs55901207 | 6 | 29905557 | 0.77 | intergenic | HCG4B(dist=10565),HLA-A(dist=4690) | - | - | - | - | - | - | 6 | - | - | - | - |
| rs62388798 | 6 | 29905957 | 0.87 | intergenic | HCG4B(dist=10965),HLA-A(dist=4290) | - | - | - | - | - | - | 7 | - | - | - | - |
| rs62388799 | 6 | 29906026 | 0.90 | intergenic | HCG4B(dist=11034),HLA-A(dist=4221) | - | - | - | - | - | - | 6 | - | - | - | - |
| rs56147022 | 6 | 29906533 | 0.90 | intergenic | HCG4B(dist=11541),HLA-A(dist=3714) | - | - | - | - | - | - | 5 | - | - | - | - |
| rs62388801 | 6 | 29906557 | 0.89 | intergenic | HCG4B(dist=11565),HLA-A(dist=3690) | - | - | - | - | - | - | 5 | - | - | - | - |
| rs3916500 | 6 | 29906776 | 0.90 | intergenic | HCG4B(dist=11784),HLA-A(dist=3471) | - | - | - | - | - | - | 3a | - | - | - | - |
| rs116395083 | 6 | 29907486 | 0.90 | intergenic | HCG4B(dist=12494),HLA-A(dist=2761) | - | - | - | - | - | - | 5 | - | - | - | - |
| rs62388802 | 6 | 29907824 | 0.90 | intergenic | HCG4B(dist=12832),HLA-A(dist=2423) | - | - | - | - | - | - | 5 | - | - | - | - |
| rs41541221 | 6 | 29910033 | 0.90 | upstream | HLA-A | Y | Y | Y | - | - | - | 4 | - | - | - | - |
| rs41545520 | 6 | 29910265 | 0.90 | UTR5 | HLA-A(NM_001242758:c.-660>0,NM_002116:c.-660>0) | Y | - | Y | Y | Y | - | 3a | - | - | - | - |
| rs114945359 | 6 | 29910324 | 0.90 | UTR5 | HLA-A(NM_001242758:c.-70>0,NM_002116:c.-70>0) | Y | - | Y | - | Y | - | 2b | - | - | - | - |
| rs41540614 | 6 | 29910865 | 0.89 | intronic | HLA-A | Y | - | Y | - | - | - | 4 | - | - | - | - |
| rs41547618 | 6 | 29911005 | 0.87 | intronic | HLA-A | Y | - | Y | - | - | - | 2b | - | - | - | - |
| rs41562213 | 6 | 29911011 | 0.87 | intronic | HLA-A | Y | - | Y | - | - | - | 4 | - | - | - | - |
| rs3132687 | 6 | 29911356 | 0.62 | intronic | HLA-A | Y | - | Y | - | - | - | 4 | - | - | - | - |
| rs41551619 | 6 | 29912410 | 0.90 | intronic | HLA-A | - | - | - | - | - | - | 3a | - | - | - | - |
| rs41545117 | 6 | 29912614 | 0.90 | intronic | HLA-A | - | - | - | - | - | - | 4 | - | - | - | - |
| rs41545022 | 6 | 29913386 | 0.90 | UTR3 | HLA-A(NM_001242758:c.*1540>0,NM_002116:c.*1540>0) | - | - | - | - | - | - | 2b | - | - | - | - |
| rs62390510 | 6 | 29913708 | 0.90 | downstream | HLA-A | - | - | - | - | - | - | 4 | - | - | - | - |
| rs56171155 | 6 | 29915117 | 0.90 | intergenic | HLA-A(dist=1456),HCG9(dist=27775) | - | - | - | - | - | - | 4 | - | - | - | - |
| rs62390518 | 6 | 29916351 | 0.82 | intergenic | HLA-A(dist=2690),HCG9(dist=26541) | - | - | - | - | - | - | 4 | - | - | - | - |
| rs62390553 | 6 | 29916490 | 0.90 | intergenic | HLA-A(dist=2829),HCG9(dist=26402) | - | - | - | - | - | - | 4 | - | - | - | - |
| rs56232426 | 6 | 29917505 | 0.90 | intergenic | HLA-A(dist=3844),HCG9(dist=25387) | - | - | - | - | - | - | 6 | - | - | - | - |
| rs62390555 | 6 | 29919220 | 0.90 | intergenic | HLA-A(dist=5559),HCG9(dist=23672) | - | - | - | - | - | - | 5 | - | - | - | - |
| rs114703418 | 6 | 29919670 | 0.89 | intergenic | HLA-A(dist=6009),HCG9(dist=23222) | - | - | - | - | - | - | 6 | - | - | - | - |
| rs55975201 | 6 | 29921802 | 0.85 | intergenic | HLA-A(dist=8141),HCG9(dist=21090) | - | Y | - | - | - | - | 5 | - | - | - | - |
| rs62392214 | 6 | 29923489 | 0.90 | intergenic | HLA-A(dist=9828),HCG9(dist=19403) | - | - | - | - | - | - | 7 | - | - | - | - |
| rs436003 | 6 | 29923941 | 0.63 | intergenic | HLA-A(dist=10280),HCG9(dist=18951) | - | - | - | - | - | - | 5 | - | - | - | - |
| rs117485380 | 6 | 29925281 | 1.00 | intergenic | HLA-A(dist=11620),HCG9(dist=17611) | - | - | - | - | - | - | 7 | - | - | - | - |
| rs146567523 | 6 | 29925558 | 1.00 | intergenic | HLA-A(dist=11897),HCG9(dist=17334) | - | - | - | - | - | - | 6 | - | - | - | - |
| rs62392216 | 6 | 29925877 | 1.00 | intergenic | HLA-A(dist=12216),HCG9(dist=17015) | - | - | - | - | - | - | 5 | - | - | - | - |
| rs115200927 | 6 | 29926389 | 1.00 | intergenic | HLA-A(dist=12728),HCG9(dist=16503) | - | - | - | - | - | - | 6 | - | - | - | - |
| rs115280716 | 6 | 29926415 | 1.00 | intergenic | HLA-A(dist=12754),HCG9(dist=16477) | - | - | - | - | - | - | 7 | - | - | - | - |
| rs114905642 | 6 | 29927014 | 1.00 | intergenic | HLA-A(dist=13353),HCG9(dist=15878) | - | - | - | - | - | - | 7 | - | - | - | - |
| rs55897388 | 6 | 29928188 | 1.00 | intergenic | HLA-A(dist=14527),HCG9(dist=14704) | - | - | - | - | - | - | 7 | - | - | - | - |
| rs116174003 | 6 | 29928557 | 1.00 | intergenic | HLA-A(dist=14896),HCG9(dist=14335) | - | - | - | - | - | - | 5 | - | - | - | - |
| rs17179992 | 6 | 29929324 | 1.00 | intergenic | HLA-A(dist=15663),HCG9(dist=13568) | - | - | - | - | - | - | 7 | - | - | - | - |
| rs62388660 | 6 | 29930402 | 1.00 | intergenic | HLA-A(dist=16741),HCG9(dist=12490) | - | - | - | - | - | - | 5 | - | - | - | - |
| rs56262516 | 6 | 29930939 | 0.93 | intergenic | HLA-A(dist=17278),HCG9(dist=11953) | - | - | - | - | - | - | 7 | - | - | - | - |
| rs56052321 | 6 | 29931646 | 1.00 | intergenic | HLA-A(dist=17985),HCG9(dist=11246) | Y | Y | Y | - | - | - | 7 | - | - | - | - |
| rs9260734 | 6 | 29932666 | 0.64 | intergenic | HLA-A(dist=19005),HCG9(dist=10226) | Y | Y | Y | - | Y | - | 4 | - | - | - | - |
| rs9260735 | 6 | 29932685 | 0.63 | intergenic | HLA-A(dist=19024),HCG9(dist=10207) | Y | Y | Y | Y | Y | - | 4 | - | - | - | - |
| rs3873283 | 6 | 29933000 | 0.64 | intergenic | HLA-A(dist=19339),HCG9(dist=9892) | Y | Y | Y | - | Y | - | 4 | - | - | - | - |
| **rs3869062** | 6 | 29934891 | 1.00 | intergenic | HLA-A(dist=21230),HCG9(dist=8001) | Y | Y | Y | - | - | - | 1f | - | - | - | - |
| rs17180093 | 6 | 29935997 | 1.00 | intergenic | HLA-A(dist=22336),HCG9(dist=6895) | Y | Y | Y | - | - | - | 3a | - | - | - | - |
| rs17180107 | 6 | 29936106 | 1.00 | intergenic | HLA-A(dist=22445),HCG9(dist=6786) | Y | Y | Y | - | - | - | 4 | - | - | - | - |
| rs138973185 | 6 | 29937222 | 1.00 | intergenic | HLA-A(dist=23561),HCG9(dist=5670) | - | Y | - | - | - | - | 7 | - | - | - | - |
| rs6939037 | 6 | 29937971 | 0.79 | intergenic | HLA-A(dist=24310),HCG9(dist=4921) | - | - | - | - | - | - | 5 | - | - | - | - |
| rs17186566 | 6 | 29938293 | 1.00 | intergenic | HLA-A(dist=24632),HCG9(dist=4599) | - | - | - | - | - | - | 7 | - | - | - | - |
| rs17180227 | 6 | 29938736 | 1.00 | intergenic | HLA-A(dist=25075),HCG9(dist=4156) | - | - | - | - | - | - | 5 | - | - | - | - |
| rs9260794 | 6 | 29939677 | 0.61 | intergenic | HLA-A(dist=26016),HCG9(dist=3215) | - | - | - | - | - | - | 1f | - | - | - | - |
| rs2394249 | 6 | 29940786 | 0.98 | intergenic | HLA-A(dist=27125),HCG9(dist=2106) | - | - | - | - | - | - | 5 | - | - | - | - |
| rs56289102 | 6 | 29941011 | 0.99 | intergenic | HLA-A(dist=27350),HCG9(dist=1881) | - | - | - | - | - | - | 6 | - | - | - | - |
| rs9260816 | 6 | 29941811 | 0.61 | intergenic | HLA-A(dist=28150),HCG9(dist=1081) | - | - | - | - | - | - | 5 | - | - | - | - |
| rs62388723 | 6 | 29942489 | 0.99 | upstream | HCG9 | - | - | - | - | - | - | 5 | - | - | - | - |
| rs2893999 | 6 | 29943832 | 0.99 | ncRNA_intronic | HCG9 | Y | Y | Y | - | - | - | 1b | - | - | - | - |
| rs3823368 | 6 | 29943906 | 0.99 | ncRNA_intronic | HCG9 | Y | Y | Y | - | - | - | 2b | - | - | - | - |
| rs3873291 | 6 | 29948941 | 0.82 | intergenic | HCG9(dist=2764),ZNRD1-AS1(dist=19847) | - | Y | - | - | - | - | 7 | - | - | - | - |
| rs139339676 | 6 | 29949224 | 0.83 | intergenic | HCG9(dist=3047),ZNRD1-AS1(dist=19564) | - | Y | - | - | - | - | 4 | - | - | - | - |
| rs56149225 | 6 | 29949843 | 0.83 | intergenic | HCG9(dist=3666),ZNRD1-AS1(dist=18945) | - | - | - | - | - | - | 7 | - | - | - | - |
| rs16896851 | 6 | 29959640 | 0.82 | intergenic | HCG9(dist=13463),ZNRD1-AS1(dist=9148) | - | - | - | - | - | - | 7 | - | - | - | - |
| rs17180836 | 6 | 29961566 | 0.83 | intergenic | HCG9(dist=15389),ZNRD1-AS1(dist=7222) | - | - | - | - | - | - | 4 | - | - | - | - |
| rs574372061 | 6 | 29969135 | 0.82 | ncRNA_exonic | ZNRD1-AS1 | - | - | - | - | - | - | - | - | - | - | - |
| rs58161072 | 6 | 29971574 | 0.82 | ncRNA_intronic | ZNRD1-AS1 | - | - | - | - | - | - | 6 | - | - | - | - |
| rs17193739 | 6 | 29972824 | 0.82 | ncRNA_intronic | ZNRD1-AS1 | - | - | - | - | - | - | 5 | - | - | - | - |
| rs2286404 | 6 | 29973620 | 0.82 | ncRNA_intronic | ZNRD1-AS1 | - | - | - | - | - | - | 5 | - | - | - | - |
| rs16896044 | 6 | 29981750 | 0.82 | ncRNA_intronic | ZNRD1-AS1 | - | - | - | - | - | - | 5 | - | - | - | - |
| rs62388749 | 6 | 29982041 | 0.81 | ncRNA_intronic | ZNRD1-AS1 | - | - | - | - | - | - | 6 | - | - | - | - |
| rs141548494 | 6 | 29983301 | 0.82 | ncRNA_intronic | ZNRD1-AS1 | - | - | - | - | - | - | 7 | - | - | - | - |
| rs56073777 | 6 | 29985492 | 0.82 | ncRNA_intronic | ZNRD1-AS1 | - | - | - | - | - | - | 7 | - | - | - | - |
| rs62388750 | 6 | 29993421 | 0.82 | ncRNA_intronic | ZNRD1-AS1 | - | - | - | - | - | - | 6 | - | - | - | - |
| rs62388751 | 6 | 29995704 | 0.82 | ncRNA_intronic | ZNRD1-AS1 | - | - | - | - | - | - | 6 | - | - | - | - |
| rs62388752 | 6 | 29997976 | 0.82 | ncRNA_intronic | ZNRD1-AS1 | - | - | - | - | - | - | 6 | - | - | - | - |
| rs16896923 | 6 | 30000687 | 0.82 | ncRNA_intronic | ZNRD1-AS1 | - | - | - | - | - | - | 6 | - | - | - | - |
| rs62388754 | 6 | 30001703 | 0.82 | ncRNA_intronic | ZNRD1-AS1 | - | - | - | - | - | - | 6 | - | - | - | - |
| rs62388755 | 6 | 30006805 | 0.82 | ncRNA_intronic | ZNRD1-AS1 | - | - | - | - | - | - | 6 | - | - | - | - |
| rs114315683 | 6 | 30012624 | 0.82 | ncRNA_intronic | ZNRD1-AS1 | - | - | - | - | - | - | 2b | - | - | - | - |
| rs62388756 | 6 | 30012760 | 0.82 | ncRNA_intronic | ZNRD1-AS1 | - | - | - | - | - | - | 5 | - | - | - | - |
| rs62388757 | 6 | 30015254 | 0.82 | ncRNA_intronic | ZNRD1-AS1 | - | - | - | - | - | - | 4 | - | - | - | - |
| rs56185319 | 6 | 30016074 | 0.82 | ncRNA_intronic | ZNRD1-AS1 | - | - | - | - | - | - | 6 | - | - | - | - |
| rs55664658 | 6 | 30018349 | 0.82 | ncRNA_intronic | ZNRD1-AS1 | - | - | - | - | - | - | 6 | - | - | - | - |
| rs56378970 | 6 | 30020674 | 0.82 | ncRNA_intronic | ZNRD1-AS1 | - | Y | - | - | - | - | 7 | - | - | - | - |
| rs56181708 | 6 | 30020826 | 0.82 | ncRNA_intronic | ZNRD1-AS1 | - | Y | - | - | - | - | 6 | - | - | - | - |
| rs62390160 | 6 | 30022598 | 0.82 | ncRNA_intronic | ZNRD1-AS1 | - | Y | - | - | - | - | 6 | - | - | - | - |
| rs55808509 | 6 | 30023771 | 0.82 | ncRNA_intronic | ZNRD1-AS1 | - | Y | - | - | - | - | 5 | - | - | - | - |
| rs10484549 | 6 | 30026290 | 0.82 | ncRNA_intronic | ZNRD1-AS1 | - | Y | - | - | - | - | 6 | - | - | - | - |
| rs62390162 | 6 | 30026768 | 0.82 | ncRNA_intronic | ZNRD1-AS1 | - | Y | - | - | - | - | 5 | - | - | - | - |
| rs56270681 | 6 | 30031456 | 0.82 | intronic | ZNRD1 | - | Y | - | - | - | - | 7 | - | - | - | - |
| rs16896970 | 6 | 30032917 | 0.82 | downstream | ZNRD1 | - | Y | Y | - | - | - | 7 | - | - | - | - |
| rs17187693 | 6 | 30036333 | 0.82 | intronic | PPP1R11 | Y | Y | Y | - | - | - | 5 | - | - | - | - |
| rs62390181 | 6 | 30041036 | 0.82 | intronic | RNF39 | - | Y | - | - | - | - | 5 | - | - | - | - |
| rs117034491 | 6 | 30048227 | 0.61 | intergenic | RNF39(dist=4599),TRIM31(dist=22447) | - | - | - | - | - | - | 5 | - | - | - | - |
| rs371857339 | 6 | 30048623 | 0.61 | intergenic | RNF39(dist=4995),TRIM31(dist=22051) | - | - | - | - | - | - | 7 | - | - | - | - |
| rs57969034 | 6 | 30049801 | 0.61 | intergenic | RNF39(dist=6173),TRIM31(dist=20873) | - | - | - | - | - | - | 7 | - | - | - | - |
| rs141224260 | 6 | 30057883 | 0.61 | intergenic | RNF39(dist=14255),TRIM31(dist=12791) | Y | Y | - | - | - | - | 7 | - | - | - | - |
| rs60774507 | 6 | 30060409 | 0.61 | intergenic | RNF39(dist=16781),TRIM31(dist=10265) | Y | Y | Y | - | - | - | 6 | - | - | - | - |
| rs117773791 | 6 | 30063301 | 0.61 | intergenic | RNF39(dist=19673),TRIM31(dist=7373) | Y | Y | Y | - | Y | - | 3a | - | - | - | - |
| rs7185039 | 16 | 74588044 | 0.62 | intronic | GLG1 | - | Y | - | - | - | - | 3a | - | - | - | - |
| rs34343506 | 16 | 74631873 | 0.72 | intronic | GLG1 | - | - | - | - | - | - | 6 | - | - | - | - |
| rs8051148 | 16 | 74647205 | 0.89 | intergenic | GLG1(dist=6163),RFWD3(dist=8092) | - | - | - | - | - | - | 7 | - | - | - | - |
| rs11377830 | 16 | 74650503 | 0.79 | intergenic | GLG1(dist=9461),RFWD3(dist=4794) | - | - | - | - | - | - | 6 | - | - | - | - |
| rs12933037 | 16 | 74660174 | 0.97 | intronic | RFWD3 | - | Y | - | - | - | - | 5 | - | - | - | - |
| rs8050262 | 16 | 74661159 | 0.97 | intronic | RFWD3 | - | Y | - | - | - | - | 5 | - | - | - | - |
| **rs7193541** | 16 | 74664743 | 1.00 | exonic | RFWD3 | - | - | - | - | - | missense | 6 | 0.472 | T | 0.008 | B |
| rs28616016 | 16 | 74676964 | 0.93 | intronic | RFWD3 | - | - | - | - | - | - | 7 | - | - | - | - |
| rs56143602 | 16 | 74694692 | 0.80 | intronic | RFWD3 | - | Y | Y | - | - | - | 5 | - | - | - | - |
| rs7199068 | 16 | 74698263 | 0.79 | intronic | RFWD3 | - | Y | Y | - | - | - | 5 | - | - | - | - |
| rs62053585 | 16 | 74700114 | 0.69 | intronic | RFWD3 | Y | Y | Y | - | - | - | 2b | - | - | - | - |
| rs4402594 | 16 | 74701360 | 0.79 | upstream | RFWD3 | Y | Y | Y | - | - | - | 1f | - | - | - | - |
| rs7184423 | 16 | 74709737 | 0.76 | intronic | MLKL | - | - | - | - | - | - | 7 | - | - | - | - |
| rs7405776 | 17 | 36093022 | 0.60 | intronic | HNF1B | Y | - | Y | - | - | - | 5 | - | - | - | - |
| rs2005705 | 17 | 36096300 | 0.74 | intronic | HNF1B | Y | Y | Y | - | - | - | 6 | - | - | - | - |
| rs538954447 | 17 | 36097661 | 0.61 | intronic | HNF1B | Y | Y | Y | - | - | - | - | - | - | - | - |
| rs11263761 | 17 | 36097775 | 0.77 | intronic | HNF1B | Y | Y | Y | - | - | - | 7 | - | - | - | - |
| rs4430796 | 17 | 36098040 | 0.85 | intronic | HNF1B | Y | Y | Y | - | Y | - | 4 | - | - | - | - |
| rs4239217 | 17 | 36098987 | 0.69 | intronic | HNF1B | Y | Y | Y | - | Y | - | 4 | - | - | - | - |
| rs11651755 | 17 | 36099840 | 0.95 | intronic | HNF1B | Y | Y | Y | - | - | - | 5 | - | - | - | - |
| rs10908278 | 17 | 36099952 | 0.93 | intronic | HNF1B | Y | Y | Y | - | - | - | 5 | - | - | - | - |
| rs11657964 | 17 | 36100767 | 0.81 | intronic | HNF1B | Y | Y | Y | - | - | - | 5 | - | - | - | - |
| rs7501939 | 17 | 36101156 | 0.81 | intronic | HNF1B | Y | Y | Y | - | Y | - | 4 | - | - | - | - |
| **rs8064454** | 17 | 36101586 | 1.00 | intronic | HNF1B | Y | Y | Y | - | - | - | 2b | - | - | - | - |
| rs12601991 | 17 | 36101633 | 0.64 | intronic | HNF1B | Y | Y | Y | - | - | - | 3a | - | - | - | - |
| rs11263762 | 17 | 36101926 | 0.64 | intronic | HNF1B | Y | Y | Y | Y | Y | - | 2b | - | - | - | - |
| rs7405696 | 17 | 36102035 | 0.63 | intronic | HNF1B | Y | Y | Y | - | Y | - | 4 | - | - | - | - |
| rs11651052 | 17 | 36102381 | 0.99 | intronic | HNF1B | Y | - | Y | - | - | - | 3a | - | - | - | - |
| rs11263763 | 17 | 36103565 | 0.96 | intronic | HNF1B | Y | - | Y | - | Y | - | 3a | - | - | - | - |
| rs11658063 | 17 | 36103872 | 0.78 | intronic | HNF1B | Y | - | Y | - | Y | - | 4 | - | - | - | - |

BP: base position.

^a^ Based on the combined European and Asian populations in 1000 Genome.

^b^ Histone modifications were derived from ENCODE A549 cell line (Broad Institute).

**Supplementary Table 9. The associations of identified related genes with lung cancer risk from gene-based analysis.**

| **Gene** | **Chr** | **Start** | **Stop** | **NSNPS^a^** | **NPARAM^b^** | **Z value** | **P value** |
| --- | --- | --- | --- | --- | --- | --- | --- |
| MAST2 | 1 | 46259269 | 46511796 | 400 | 8 | 2.91 | 1.79E-03 |
| MICA | 6 | 31357561 | 31393090 | 306 | 18 | 2.78 | 2.75E-03 |
| HLA-G | 6 | 29784756 | 29808899 | 315 | 16 | 1.92 | 2.72E-02 |
| FADS1 | 11 | 61557097 | 61594529 | 32 | 2 | 4.98 | 3.12E-07 |
| RFWD3 | 16 | 74645297 | 74710779 | 160 | 11 | 2.90 | 1.88E-03 |
| HNF1B | 17 | 36036434 | 36115096 | 118 | 29 | 2.20 | 1.38E-02 |

^a^ The number of SNPs annotated to gene.

^b^ The number of relevant parameters used in the model.

**Supplementary Table 10. KEGG pathway enrichment results of co-expressed genes with identified related genes in lung tissue.**

| **Gene** | **ID** | **Description** | **GeneRatio** | **BgRatio** | **pvalue** | **p.adjust** |
| --- | --- | --- | --- | --- | --- | --- |
| MAST2 | hsa04142 | Lysosome | 77/3064 | 123/7470 | 9.30E-07 | 2.90E-04 |
|  | hsa00640 | Propanoate metabolism | 26/3064 | 32/7470 | 3.70E-06 | 5.80E-04 |
|  | hsa00280 | Valine, leucine and isoleucine degradation | 34/3064 | 48/7470 | 2.70E-05 | 2.10E-03 |
|  | hsa01212 | Fatty acid metabolism | 34/3064 | 48/7470 | 2.70E-05 | 2.10E-03 |
|  | hsa00563 | Glycosylphosphatidylinositol (GPI)-anchor biosynthesis | 20/3064 | 25/7470 | 8.00E-05 | 5.00E-03 |
|  | hsa04146 | Peroxisome | 50/3064 | 83/7470 | 3.00E-04 | 1.60E-02 |
|  | hsa00562 | Inositol phosphate metabolism | 45/3064 | 74/7470 | 4.40E-04 | 2.00E-02 |
|  | hsa05225 | **Hepatocellular carcinoma** | 90/3064 | 168/7470 | 6.00E-04 | 2.40E-02 |
|  | hsa01200 | Carbon metabolism | 65/3064 | 116/7470 | 7.10E-04 | 2.50E-02 |
|  | hsa00062 | Fatty acid elongation | 21/3064 | 30/7470 | 1.20E-03 | 3.80E-02 |
|  | hsa05144 | Malaria | 31/3064 | 49/7470 | 1.30E-03 | 3.80E-02 |
|  | hsa00230 | Purine metabolism | 91/3064 | 174/7470 | 1.50E-03 | 3.90E-02 |
|  | hsa03030 | **DNA replication** | 24/3064 | 36/7470 | 1.60E-03 | 3.90E-02 |
|  | hsa04714 | Thermogenesis | 116/3064 | 229/7470 | 1.70E-03 | 3.90E-02 |
|  | hsa03020 | **RNA polymerase** | 21/3064 | 31/7470 | 2.40E-03 | 5.00E-02 |
| MICA | hsa04142 | Lysosome | 78/2919 | 123/7441 | 4.00E-08 | 1.30E-05 |
|  | hsa00640 | Propanoate metabolism | 25/2919 | 32/7441 | 8.20E-06 | 9.00E-04 |
|  | hsa00280 | Valine, leucine and isoleucine degradation | 34/2919 | 48/7441 | 8.60E-06 | 9.00E-04 |
|  | hsa04068 | FoxO signaling pathway | 76/2919 | 132/7441 | 1.30E-05 | 1.00E-03 |
|  | hsa04668 | **TNF signaling pathway** | 65/2919 | 110/7441 | 1.70E-05 | 1.10E-03 |
|  | hsa04010 | **MAPK signaling pathway** | 150/2919 | 295/7441 | 2.40E-05 | 1.30E-03 |
|  | hsa05144 | Malaria | 33/2919 | 49/7441 | 6.00E-05 | 2.70E-03 |
|  | hsa01200 | Carbon metabolism | 66/2919 | 116/7441 | 7.90E-05 | 2.80E-03 |
|  | hsa04146 | Peroxisome | 50/2919 | 83/7441 | 8.00E-05 | 2.80E-03 |
|  | hsa00240 | Pyrimidine metabolism | 58/2919 | 101/7441 | 1.50E-04 | 4.70E-03 |
|  | hsa05142 | Chagas disease (American trypanosomiasis) | 58/2919 | 102/7441 | 2.10E-04 | 6.10E-03 |
|  | hsa05169 | Epstein-Barr virus infection | 103/2919 | 201/7441 | 3.10E-04 | 8.10E-03 |
|  | hsa03460 | Fanconi anemia pathway | 34/2919 | 54/7441 | 3.40E-04 | 8.30E-03 |
|  | hsa04012 | ErbB signaling pathway | 49/2919 | 85/7441 | 4.20E-04 | 9.40E-03 |
|  | hsa03440 | Homologous recombination | 27/2919 | 41/7441 | 4.90E-04 | 9.80E-03 |
|  | hsa05166 | Human T-cell leukemia virus 1 infection | 126/2919 | 255/7441 | 5.00E-04 | 9.80E-03 |
|  | hsa05133 | Pertussis | 44/2919 | 76/7441 | 7.20E-04 | 1.20E-02 |
|  | hsa00562 | Inositol phosphate metabolism | 43/2919 | 74/7441 | 7.40E-04 | 1.20E-02 |
|  | hsa04210 | Apoptosis | 72/2919 | 136/7441 | 7.40E-04 | 1.20E-02 |
|  | hsa03030 | **DNA replication** | 24/2919 | 36/7441 | 7.70E-04 | 1.20E-02 |
|  | hsa00563 | Glycosylphosphatidylinositol (GPI)-anchor biosynthesis | 18/2919 | 25/7441 | 9.00E-04 | 1.40E-02 |
|  | hsa01521 | **EGFR tyrosine kinase inhibitor resistance** | 45/2919 | 79/7441 | 1.00E-03 | 1.40E-02 |
|  | hsa04150 | **mTOR signaling pathway** | 78/2919 | 151/7441 | 1.20E-03 | 1.60E-02 |
|  | hsa05143 | African trypanosomiasis | 23/2919 | 35/7441 | 1.30E-03 | 1.80E-02 |
|  | hsa05225 | **Hepatocellular carcinoma** | 85/2919 | 168/7441 | 1.60E-03 | 2.10E-02 |
|  | hsa05223 | **Non-small cell lung cancer** | 38/2919 | 66/7441 | 1.90E-03 | 2.30E-02 |
|  | hsa00020 | Citrate cycle (TCA cycle) | 20/2919 | 30/7441 | 2.10E-03 | 2.50E-02 |
|  | hsa00230 | Purine metabolism | 87/2919 | 174/7441 | 2.30E-03 | 2.50E-02 |
|  | hsa01212 | Fatty acid metabolism | 29/2919 | 48/7441 | 2.30E-03 | 2.50E-02 |
|  | hsa04120 | Ubiquitin mediated proteolysis | 70/2919 | 137/7441 | 2.90E-03 | 3.10E-02 |
|  | hsa04370 | VEGF signaling pathway | 34/2919 | 59/7441 | 3.10E-03 | 3.20E-02 |
|  | hsa03020 | **RNA polymerase** | 20/2919 | 31/7441 | 3.80E-03 | 3.70E-02 |
|  | hsa04014 | **Ras signaling pathway** | 111/2919 | 232/7441 | 4.10E-03 | 4.00E-02 |
|  | hsa00511 | Other glycan degradation | 13/2919 | 18/7441 | 4.70E-03 | 4.30E-02 |
|  | hsa04310 | **Wnt signaling pathway** | 73/2919 | 146/7441 | 4.90E-03 | 4.40E-02 |
|  | hsa05215 | **Prostate cancer** | 51/2919 | 97/7441 | 5.00E-03 | 4.40E-02 |
|  | hsa00520 | Amino sugar and nucleotide sugar metabolism | 28/2919 | 48/7441 | 5.60E-03 | 4.70E-02 |
| HLA-G | hsa03050 | Proteasome | 27/504 | 45/7469 | 6.90E-21 | 2.00E-18 |
|  | hsa00190 | Oxidative phosphorylation | 33/504 | 133/7469 | 2.70E-11 | 3.90E-09 |
|  | hsa05012 | Parkinson disease | 31/504 | 142/7469 | 3.30E-09 | 3.20E-07 |
|  | hsa05016 | Huntington disease | 37/504 | 193/7469 | 4.50E-09 | 3.30E-07 |
|  | hsa04714 | Thermogenesis | 40/504 | 229/7469 | 1.70E-08 | 1.00E-06 |
|  | hsa04932 | Non-alcoholic fatty liver disease (NAFLD) | 30/504 | 149/7469 | 4.30E-08 | 2.10E-06 |
|  | hsa05010 | Alzheimer disease | 32/504 | 171/7469 | 9.30E-08 | 3.90E-06 |
|  | hsa05169 | Epstein-Barr virus infection | 35/504 | 201/7469 | 1.50E-07 | 5.50E-06 |
|  | hsa04145 | Phagosome | 28/504 | 152/7469 | 8.40E-07 | 2.70E-05 |
|  | hsa01200 | Carbon metabolism | 23/504 | 116/7469 | 2.20E-06 | 6.40E-05 |
|  | hsa04142 | Lysosome | 23/504 | 123/7469 | 6.20E-06 | 1.70E-04 |
|  | hsa04612 | **Antigen processing and presentation** | 17/504 | 77/7469 | 1.10E-05 | 2.60E-04 |
|  | hsa00020 | Citrate cycle (TCA cycle) | 10/504 | 30/7469 | 1.60E-05 | 3.60E-04 |
|  | hsa04141 | Protein processing in endoplasmic reticulum | 26/504 | 165/7469 | 3.80E-05 | 8.00E-04 |
|  | hsa05130 | Pathogenic Escherichia coli infection | 13/504 | 55/7469 | 5.40E-05 | 1.10E-03 |
|  | hsa04260 | Cardiac muscle contraction | 15/504 | 78/7469 | 1.80E-04 | 3.40E-03 |
|  | hsa01230 | Biosynthesis of amino acids | 14/504 | 75/7469 | 4.10E-04 | 7.20E-03 |
|  | hsa04144 | Endocytosis | 31/504 | 244/7469 | 4.40E-04 | 7.20E-03 |
|  | hsa05416 | Viral myocarditis | 12/504 | 59/7469 | 4.70E-04 | 7.30E-03 |
|  | hsa05131 | Shigellosis | 12/504 | 65/7469 | 1.20E-03 | 1.70E-02 |
|  | hsa05145 | Toxoplasmosis | 17/504 | 113/7469 | 1.40E-03 | 2.00E-02 |
|  | hsa04514 | **Cell adhesion molecules (CAMs)** | 19/504 | 144/7469 | 3.60E-03 | 4.80E-02 |
| RFWD3 | hsa04110 | **Cell cycle** | 53/1038 | 124/7441 | 2.70E-15 | 8.50E-13 |
|  | hsa03050 | Proteasome | 23/1038 | 45/7441 | 3.20E-09 | 5.00E-07 |
|  | hsa03008 | Ribosome biogenesis in eukaryotes | 37/1038 | 105/7441 | 2.70E-08 | 2.80E-06 |
|  | hsa03013 | RNA transport | 51/1038 | 171/7441 | 4.30E-08 | 3.40E-06 |
|  | hsa03030 | **DNA replication** | 18/1038 | 36/7441 | 2.60E-07 | 1.60E-05 |
|  | hsa03460 | Fanconi anemia pathway | 20/1038 | 54/7441 | 1.90E-05 | 9.70E-04 |
|  | hsa04141 | Protein processing in endoplasmic reticulum | 43/1038 | 165/7441 | 2.30E-05 | 1.00E-03 |
|  | hsa03410 | Base excision repair | 14/1038 | 33/7441 | 5.90E-05 | 2.10E-03 |
|  | hsa03440 | Homologous recombination | 16/1038 | 41/7441 | 6.10E-05 | 2.10E-03 |
|  | hsa00240 | Pyrimidine metabolism | 29/1038 | 101/7441 | 7.70E-05 | 2.40E-03 |
|  | hsa00480 | Glutathione metabolism | 18/1038 | 56/7441 | 3.90E-04 | 1.00E-02 |
|  | hsa00230 | Purine metabolism | 41/1038 | 174/7441 | 3.90E-04 | 1.00E-02 |
|  | hsa01200 | Carbon metabolism | 30/1038 | 116/7441 | 4.50E-04 | 1.10E-02 |
|  | hsa03060 | Protein export | 10/1038 | 23/7441 | 5.40E-04 | 1.20E-02 |
|  | hsa04114 | Oocyte meiosis | 31/1038 | 124/7441 | 6.80E-04 | 1.40E-02 |
|  | hsa04115 | **p53 signaling pathway** | 20/1038 | 72/7441 | 1.50E-03 | 2.90E-02 |
|  | hsa03430 | Mismatch repair | 9/1038 | 23/7441 | 2.50E-03 | 4.50E-02 |
|  | hsa03040 | Spliceosome | 31/1038 | 134/7441 | 2.60E-03 | 4.50E-02 |
| HNF1B | hsa04142 | Lysosome | 74/2535 | 123/7441 | 2.20E-09 | 7.10E-07 |
|  | hsa04070 | Phosphatidylinositol signaling system | 58/2535 | 99/7441 | 4.40E-07 | 6.90E-05 |
|  | hsa01212 | Fatty acid metabolism | 33/2535 | 48/7441 | 9.30E-07 | 9.70E-05 |
|  | hsa00280 | Valine, leucine and isoleucine degradation | 32/2535 | 48/7441 | 3.80E-06 | 3.00E-04 |
|  | hsa00562 | Inositol phosphate metabolism | 44/2535 | 74/7441 | 6.30E-06 | 4.00E-04 |
|  | hsa00020 | Citrate cycle (TCA cycle) | 22/2535 | 30/7441 | 1.30E-05 | 6.60E-04 |
|  | hsa05165 | Human papillomavirus infection | 152/2535 | 339/7441 | 1.70E-05 | 7.50E-04 |
|  | hsa00640 | Propanoate metabolism | 22/2535 | 32/7441 | 6.30E-05 | 2.50E-03 |
|  | hsa04150 | **mTOR signaling pathway** | 74/2535 | 151/7441 | 9.20E-05 | 3.20E-03 |
|  | hsa05143 | African trypanosomiasis | 23/2535 | 35/7441 | 1.30E-04 | 4.00E-03 |
|  | hsa04310 | **Wnt signaling pathway** | 71/2535 | 146/7441 | 1.70E-04 | 4.90E-03 |
|  | hsa01210 | 2-Oxocarboxylic acid metabolism | 14/2535 | 18/7441 | 1.90E-04 | 4.90E-03 |
|  | hsa00062 | Fatty acid elongation | 20/2535 | 30/7441 | 2.60E-04 | 5.90E-03 |
|  | hsa01200 | Carbon metabolism | 58/2535 | 116/7441 | 2.60E-04 | 5.90E-03 |
|  | hsa04390 | Hippo signaling pathway | 73/2535 | 154/7441 | 3.70E-04 | 7.70E-03 |
|  | hsa04068 | FoxO signaling pathway | 64/2535 | 132/7441 | 3.90E-04 | 7.70E-03 |
|  | hsa04750 | Inflammatory mediator regulation of TRP channels | 50/2535 | 99/7441 | 5.10E-04 | 8.60E-03 |
|  | hsa05225 | **Hepatocellular carcinoma** | 78/2535 | 168/7441 | 5.30E-04 | 8.60E-03 |
|  | hsa01040 | Biosynthesis of unsaturated fatty acids | 16/2535 | 23/7441 | 5.40E-04 | 8.60E-03 |
|  | hsa04012 | **ErbB signaling pathway** | 44/2535 | 85/7441 | 5.50E-04 | 8.60E-03 |
|  | hsa00071 | Fatty acid degradation | 26/2535 | 44/7441 | 5.70E-04 | 8.60E-03 |
|  | hsa04360 | Axon guidance | 80/2535 | 175/7441 | 8.20E-04 | 1.20E-02 |
|  | hsa04071 | Sphingolipid signaling pathway | 57/2535 | 118/7441 | 8.90E-04 | 1.20E-02 |
|  | hsa00620 | Pyruvate metabolism | 23/2535 | 39/7441 | 1.20E-03 | 1.60E-02 |
|  | hsa04960 | Aldosterone-regulated sodium reabsorption | 22/2535 | 37/7441 | 1.30E-03 | 1.70E-02 |
|  | hsa04014 | **Ras signaling pathway** | 101/2535 | 232/7441 | 1.50E-03 | 1.80E-02 |
|  | hsa01521 | **EGFR tyrosine kinase inhibitor resistance** | 40/2535 | 79/7441 | 1.70E-03 | 2.00E-02 |
|  | hsa04916 | Melanogenesis | 49/2535 | 101/7441 | 1.80E-03 | 2.00E-02 |
|  | hsa05144 | Malaria | 27/2535 | 49/7441 | 1.90E-03 | 2.00E-02 |
|  | hsa04966 | Collecting duct acid secretion | 17/2535 | 27/7441 | 2.00E-03 | 2.00E-02 |
|  | hsa05217 | **Basal cell carcinoma** | 33/2535 | 63/7441 | 2.00E-03 | 2.00E-02 |
|  | hsa05226 | **Gastric cancer** | 68/2535 | 149/7441 | 2.10E-03 | 2.00E-02 |
|  | hsa00100 | Steroid biosynthesis | 13/2535 | 19/7441 | 2.30E-03 | 2.20E-02 |
|  | hsa04270 | Vascular smooth muscle contraction | 56/2535 | 121/7441 | 3.40E-03 | 3.10E-02 |
|  | hsa05205 | **Proteoglycans in cancer** | 87/2535 | 201/7441 | 3.70E-03 | 3.30E-02 |
|  | hsa04933 | AGE-RAGE signaling pathway in diabetic complications | 47/2535 | 99/7441 | 3.80E-03 | 3.30E-02 |
|  | hsa04721 | Synaptic vesicle cycle | 32/2535 | 63/7441 | 4.40E-03 | 3.80E-02 |

Note: Bold fonts represent pathways involved in cancer development.
