# Supplementary material for: Cross-Cancer Pleiotropic Analysis Reveals Novel Susceptibility Loci for Lung Cancer
Source: Front Oncol. 2020 Jan 15;9:1492. doi: 10.3389/fonc.2019.01492 (PMC6974684; doi:10.3389/fonc.2019.01492)
Supplement: Supplementary file 2 [file Image_1.pdf]

Cross-cancer pleiotropic analysis reveals novel susceptibility loci for lung cancer

Supplementary Figures

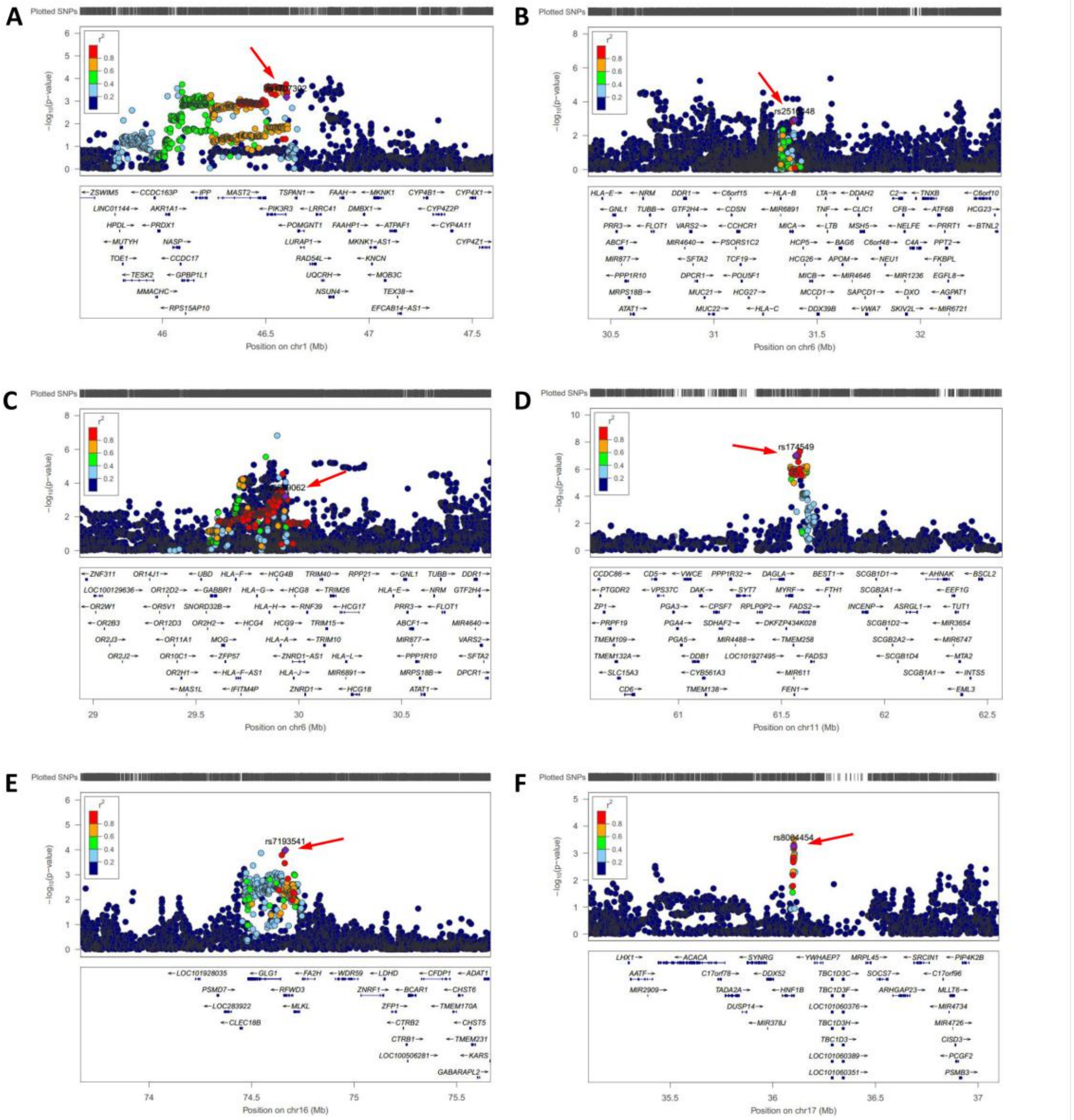

Supplementary Figure 1. The regional plot of 6 significant SNPs. (A) The regional plot of rs1707302. (B) The regional plot of rs2516448. (C) The regional plot of rs3869062. (D) The regional plot of rs174549. (E) The regional plot of rs7193541. (F) The regional plot of rs8064454.

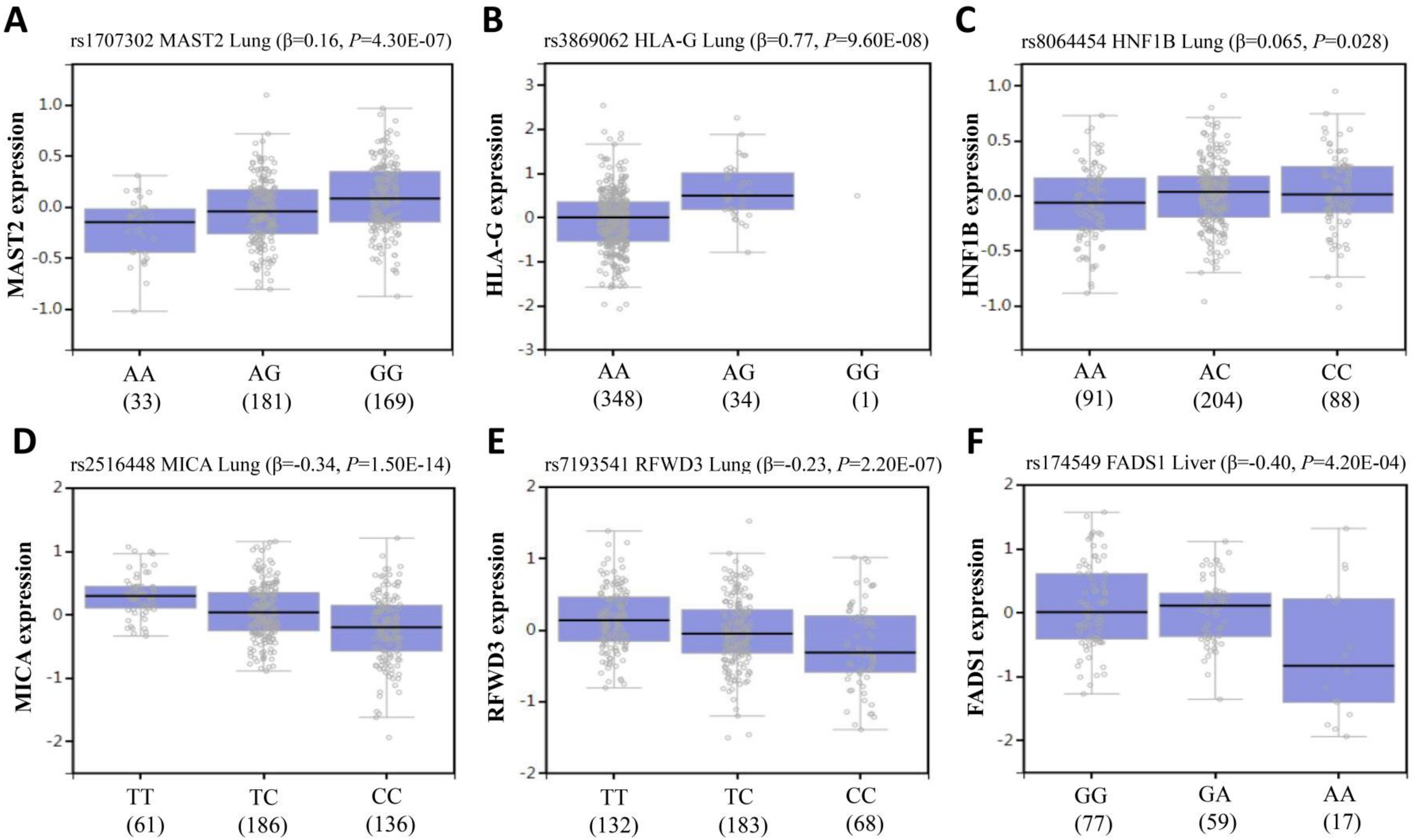
A
rs1707302 MAST2 Lung (β=0.16, P=4.30E-07)
MAST2 expression
AA (33)
AG (181)
GG (169)
B
rs3869062 HLA-G Lung (β=0.77, P=9.60E-08)
HLA-G expression
AA (348)
AG (34)
GG (1)
C
rs8064454 HNF1B Lung (β=0.065, P=0.028)
HNF1B expression
AA (91)
AC (204)
CC (88)
D
rs2516448 MICA Lung (β=-0.34, P=1.50E-14)
MICA expression
TT (61)
TC (186)
CC (136)
E
rs7193541 RFWD3 Lung (β=-0.23, P=2.20E-07)
RFWD3 expression
TT (132)
TC (183)
CC (68)
F
rs174549 FADS1 Liver (β=-0.40, P=4.20E-04)
FADS1 expression
GG (77)
GA (59)
AA (17)

Supplementary Figure 2. Box plot displaying the association of SNPs with gene expression based on GTEx dataset v7 release. (A) The association between rs1707302 genotypes and MAST2 expression in lung tissues. (B) The association between rs3869062 genotypes and HLA-G expression in lung tissues. (C) The association between rs8064454 genotypes and HNF1B expression in lung tissues. (D) The association between rs2516448 genotypes and MICA expression in lung tissues. (E) The association between rs7193541 genotypes and RFWD3 expression in lung tissues. (F) The association between rs174549 genotypes and FADS1 expression in liver tissues.
